# A sector fast encryption algorithm for color images based on one-dimensional composite sinusoidal chaos map

**DOI:** 10.1371/journal.pone.0310279

**Published:** 2025-01-24

**Authors:** Ye Tao, Wenhua Cui, Shanshan Wang, Yayun Wang

**Affiliations:** 1 School of Electronic and Information Engineering, University of Science and Technology Liaoning, Anshan, China; 2 School of Computer Science and Software Engineering, University of Science and Technology Liaoning, Anshan, China; 3 Key Laboratory of Intelligent Constriguction and Internet of Thinfigs Application Technology, Anshan, China; 4 China MCC22 Group Co., Ltd., Tangshan, China; 5 Anshan Normal University, Anshan, China; 6 School of Materials and Metallurgy, University of Science and Technology Liaoning, Anshan, China; Lanzhou University of Technology, CHINA

## Abstract

Images are important information carriers in our lives, and images should be secure when transmitted and stored. Image encryption algorithms based on chaos theory emerge in endlessly. Based on previous various chaotic image fast encryption algorithms, this paper proposes a color image sector fast encryption algorithm based on one-dimensional composite sinusoidal chaotic mapping. The main purpose of this algorithm is to improve the encryption and decryption speed of color images and improve the efficiency of image encryption in the big data era. First, four basic chaos maps are combined in pairs and added with sine operations. Six one-dimensional composite sinusoidal chaos maps (CSCM) were obtained. Secondly, select the two best chaotic mappings LCS and SCS. The randomness of these two chaotic mappings was verified through Lyapunov index and NIST SP 800–22 randomness tests. Thirdly, the encryption process is carried out according to the shape of a traditional Chinese fan, and the diffusion and scrambling of each pixel of the image are performed in parallel. This greatly improves encryption speed. When diffusing, changing the value of one pixel can affect the values of multiple subsequent pixels. When scrambling, each pixel changes position with the three pixels before it according to the chaotic sequence. Finally, through many experiments, it is proved that the image encryption algorithm not only greatly improves the encryption and decryption speed, but also improves various indexes. The key space reached 2^192^, the average information entropy was 7.9994, the average NPCR was 99.6172, and the average UACI was 33.4646. The algorithm can also resist some common attacks and accidents, such as exhaustion attack, differential attack, noise attack, information loss and so on.

## 1 Introduction

Nowadays, with the rapid development of the Internet and information technology, multimedia data transmission has become increasingly prominent. Image is a description of the similarity and vividness of objective things. It is an important information carrier in our lives. With the wide application of digital images in various fields, image encryption technology is becoming more and more important. Image encryption technology is an effective means to protect image information. It ensures the security of images during transmission and storage. Aiming at the shortcomings of existing image encryption technologies, such as low encryption algorithm efficiency, low encryption strength, etc. This can no longer meet the application requirements of large amounts of data and high confidentiality. Therefore, it becomes particularly necessary to develop an efficient image encryption algorithm. Chaos-based image encryption technology has the advantages of large key space, fast encryption speed, and strong randomness. It has become a current research hotspot.

Recently, researchers have published some typical image encryption algorithms. Zhou et al. [1] proposed a novel image encryption scheme based on chaotic signals with finite-precision error. Zhou et al. [2] proposed a novel image encryption cryptosystem based on true random numbers and chaotic systems. Zhou et al. [3] proposed a novel image cryptosystem based on new 2D hyperchaotic map and dynamical chaotic S-box. Ye et al. [4] proposed a new chaotic circuit with multiple memristors and its application in image encryption. Ye et al. [5] proposed hidden oscillation and chaotic sea in a novel 3d chaotic system with exponential function. Tao et al. [6] proposed spatiotemporal chaos in multiple dynamically coupled map lattices and its application in a novel image encryption algorithm.

Due to the advent of the big data era, more and more large batches of images need to be encrypted. This requires increasingly higher encryption speeds. The fast image encryption algorithm based on chaos has been further studied and explored based on previous research. It not only improves the encryption speed, but also ensures the security and reliability of the encryption algorithm. This has broad application prospects for practical applications.

In recent years, for rapid image encryption based on chaos, various researchers have proposed various methods with diffusion algorithms (changing pixel values) and scrambling algorithms (changing pixel positions) as the core. Various methods have been used to improve image encryption. speed. Wang et al. [7] proposed a fast image encryption algorithm based on the logical dynamic Arnold coupled logical map lattice model. Lin et al. [8] proposed a fast image encryption algorithm that enhances the randomness of chaotic sequences. Hanis et al. [9] proposed a fast encryption algorithm for block images based on improved logical mapping and butterfly structure. Stalin et al. [10] proposed a fast image encryption algorithm that combines block encryption, four-dimensional logical mapping and DNA systems. Abdelfatah [11] proposed a fast image encryption scheme based on dual chaotic pseudo-random generators. Gupta et al. [12] proposed a fast symmetric image encryption algorithm based on session keys. Talhaoui et al. [13] proposed an efficient and high-speed image fast encryption scheme based on Boolean chaos mapping. Kang et al. [14] proposed a fast encryption algorithm for color images using programmable supplementary maximum length cellular automata. Gao et al. [15] proposed a multi-image fast encryption algorithm based on single-channel scrambling, diffusion and chaotic mapping. Brahim et al. [16] proposed a fast image compression encryption scheme based on compressed sensing and parallel blocks. Tang et al. [17] proposed a fast image encryption method based on one-dimensional composite sinusoidal chaotic mapping. Fouda et al. [18] proposed a method for fast image encryption based on 8-bit precision passwords. Zhang et al. [19] proposed a parallel multi-image encryption based on cross-plane DNA manipulation and a novel 2D chaotic system.

The above image fast encryption algorithm belongs to the scrambling-diffusion image encryption algorithm. Scrambling and diffusion are two independent processes. Generally, it takes several rounds to achieve a satisfactory level of safety. This increases the time and complexity of calculations. and reduces its advantages in online image and high-volume image protection. In addition, it is not conducive to resisting separate attacks of proliferation and scrambling. Based on this, some image encryption algorithms use scrambling and diffusion to encrypt at the same time. For example, Wang et al. [20] proposed an image encryption algorithm based on parallel arrangement and diffusion strategy. And the sub-key cross-fusion method is used to form different keys in different rounds. Li et al. [21] proposed an Image encryption based on a fractional-order hyperchaotic system and fast row-column-level joint permutation and diffusion. Ma et al. [22] proposed a fast hyperchaotic image encryption scheme. Although this image encryption algorithm improves the speed of image encryption and decryption to a certain extent, it resists diffusion and scrambling attacks respectively. However, evaluation indicators such as key sensitivity, plain sensitivity, and information entropy still need to be improved. And there is still room for improvement in encryption speed.

In order to further improve the speed of image encryption and decryption and improve various evaluation indicators, this paper proposes a fast image encryption algorithm based on one-dimensional composite chaos. The specific chapters of this article are arranged as follows. Section 1 introduces the current research status of fast image encryption algorithms based on chaos, including various types of image fast encryption algorithms that perform diffusion scrambling separately and diffusion scrambling simultaneously. Section 2 introduces one-dimensional compound sinusoidal chaotic systems and analyzes their chaotic performance. Section 3 introduces the process of image encryption and decryption using the two best-performing composite chaos to generate chaotic sequences. During the image encryption process, the image is encrypted in a fan-shaped manner with associated plain. Changing the value of one point can affect the changes of multiple point values, and the scrambling and diffusion operations are performed simultaneously. Section 4 introduces the experimental results, including the comparison of various image encryption indicators such as encryption and decryption speed, correlation coefficient, histogram, information entropy, key space, key sensitivity, plain sensitivity, and robustness. It is proved that while improving the encryption speed, this algorithm ensures good key space, key sensitivity and plaintext sensitivity, can effectively resist various common attacks, and meets the needs of practical applications. Therefore, this algorithm has broad application prospects.

## 2 One-dimensional composite sinusoidal chaos map (CSCM)

In this chapter, first, four existing one-dimensional chaotic maps are introduced as seed maps. Secondly, in view of the shortcomings of existing chaos maps in weak dynamic behavior, two new one-dimensional compound sine chaos maps are proposed based on the one-dimensional compound sine chaos map (CSCM) in the literature [17]. SCS (Sine-Cubic-Sine) and TCS (Tent-Cubic-Sine). Finally, LSS (Logistic-Sine-Sine), STS (Sine-Tent-Sine), TLS (Tent-Logistic-Sine), LCS (Logistic-Cubic-Sine) and SCS, The bifurcation diagram of TCS proves that LCS and SCS have stronger chaotic properties.

### 2.1 Four typical one-dimensional chaotic maps

Most researchers use chaotic systems to generate chaotic matrices, and then perform scrambling and diffusion calculations on the chaotic matrix and plain images. We can use one-dimensional chaotic systems and multi-dimensional chaotic systems to generate chaotic images. Currently, image encryption schemes based on multi-dimensional chaotic systems are widely used [23–26]. However, due to the complex structure and numerous parameters of multi-dimensional chaotic systems, their hardware implementation becomes more difficult.

One-dimensional chaotic mapping may have the disadvantages of limited chaotic interval and uneven output state [27]. However, one-dimensional chaotic systems also have the advantages of simple structure, easy implementation, and low computational cost. In recent years, researchers have proposed a large number of image encryption algorithms based on one-dimensional chaos [28–30]. Therefore, it is of great significance to study a one-dimensional composite chaotic system with good chaotic characteristics.

There are four most commonly used one-dimensional chaos maps, including Tent chaos map, Cubic chaos map, Sine chaos map and Logistic chaos map. The definitions of these four chaotic maps are as shown in Eqs (1)–(4).


$$x_{n+1}=T(a,x_{n})=1-2a|x_{n}-{\left(2a\right)}^{-1}|$$ (1)

$$x_{n+1}=C(a,x_{n})=|4x_{n}^{3}-3ax_{n}|$$ (2)

$$x_{n+1}=S(a,x_{n})=a\sin(\pi x_{n})$$ (3)

$$x_{n+1}=L(a,x_{n})=4ax_{n}(1-x_{n})$$ (4)

Eq (1) is the Tent chaos map, Eq (2) is the Cubic chaos map, Eq (3) is the Sine chaos map, and Eq (4) is the Logistic chaos map. || represents absolute value operation, *x* is the variable of chaos iteration, and *a* ∈ (0,1) is the chaos control parameter. The combination of these four common types of chaos maps can produce many types of composite chaos maps to enhance the performance of chaos.

### 2.2 One-dimensional composite sinusoidal chaos map (CSCM) definition

In [17], a new one-dimensional compound sine chaotic system (CSCS) is introduced. CSCS is shown in Eq (5).

$$x_{n+1}=|\beta \pi \;\sin\{\pi [\mu_{1}(e,x_{n})+\mu_{2}(f,x_{n})+\theta ]\}|$$ (5)

where *μ*_1_(*e*, *x*_*n*_) and *μ*_2_(*e*, *x*_*n*_) are any two common chaotic maps introduced in Section 2.1. Chaos control parameters are *e*, *f* and *β*, *θ* ∈ [–1,1] is a variable. In the analysis of this article, the following values are set *e* = *a*, *f* = 1—*a*, *θ* = 0.5, and *β* > 0.

The four common chaotic maps in Section 2.1 are brought into Formula (5) and can form the following six combinations, among which LSS (Logistic-Sine-Sine), STS (Sine-Tent-Sine), TLS (Tent-Logistic-Sine), and LCS (Logistic-Cubic-Sine) are proposed by [16], as shown in Eqs (6)–(9),

$$x_{n+1}=|\beta \pi \;\sin\{\pi [4ax_{n}(1-x_{n})+f\;\sin(\pi x_{n})+\theta ]\}|\mathrm{mod}1$$ (6)

$$x_{n+1}=|\beta \pi \;\sin\{\pi [a\sin(\pi x_{n})+1-2f|x_{n}-0.5f^{-1}|+\theta ]\}|\mathrm{mod}\;1$$ (7)

$$x_{n+1}=|\beta \pi \;\sin\{\pi [1-2a|x_{n}-0.5a^{-1}|+4fx_{n}(1-x_{n})+\theta ]\}|\mathrm{mod}\;1$$ (8)

$$x_{n+1}=|\beta \pi \;\sin\{\pi [4ax_{n}(1-x_{n})+|4x_{n}^{3}-3fx_{n}|+\theta ]\}|\mathrm{mod}\;1$$ (9)

In Eqs (6)–(9), in order to make the value of *x* in (0, 1), this article performs mod 1 operation on each formula, that is, finding the remainder after dividing by 1.

On this basis, this paper proposes SCS (Sine-Cubic-Sine) and TCS (Tent-Cubic-Sine). In Section 2.3, it is analyzed and compared with (6)-(9), as shown in Eqs (10)–(11) shown.


$$x_{n+1}=|\beta \pi \;\sin\{\pi [a\sin(\pi x_{n})+|4x_{n}^{3}-3fx_{n}|+\theta ]\}|\mathrm{mod}\;1$$ (10)

$$x_{n+1}=|\beta \pi \;\sin\{\pi [1-2a|x_{n}-0.5a^{-1}|+|4x_{n}^{3}-3fx_{n}|+\theta ]\}|\mathrm{mod}\;1$$ (11)

The purpose of the mod 1 calculation is to normalize these chaotic maps for ease of comparison. Keep the value range of *x* between 0 and 1.

### 2.3 CSCS performance analysis

#### 2.3.1 Bifurcation diagram

This section tests and analyzes the performance of the six chaotic systems in Section 2.2 through bifurcation diagrams. The bifurcation diagram of a chaotic system is an important tool used to describe chaotic phenomena. It helps us see periodic and non-periodic behavior in chaotic systems as a parameter changes. In the bifurcation diagram, the abscissa represents the value of the system parameter. The ordinate represents a specific measurement value of the system state, such as position, angle, amplitude, etc.

By changing the control parameter *β*, we draw the bifurcation diagram of LSS, STS, TLS, LCS, SCS, and TCS generated by CSCS. Set 3 *β* values for each bifurcation diagram, which are 0.5, 50 and 200 respectively. Take the initial value *x* = 0.4. The value is from 0 to 1, incrementing by 0.001 each time. After 300 iterations, the chaotic transitional form was removed. Figs 1–6 shows the bifurcation diagrams of LSS, STS, TLS, LCS, SCS, and TCS when *β* = 0.5. Fig 7–12 shows the bifurcation diagram of LSS, STS, TLS, LCS, SCS, and TCS when *β* = 50. Fig 13–18 shows the bifurcation diagram of LSS, STS, TLS, LCS, SCS, and TCS when *β* = 200.

**Fig 1:**
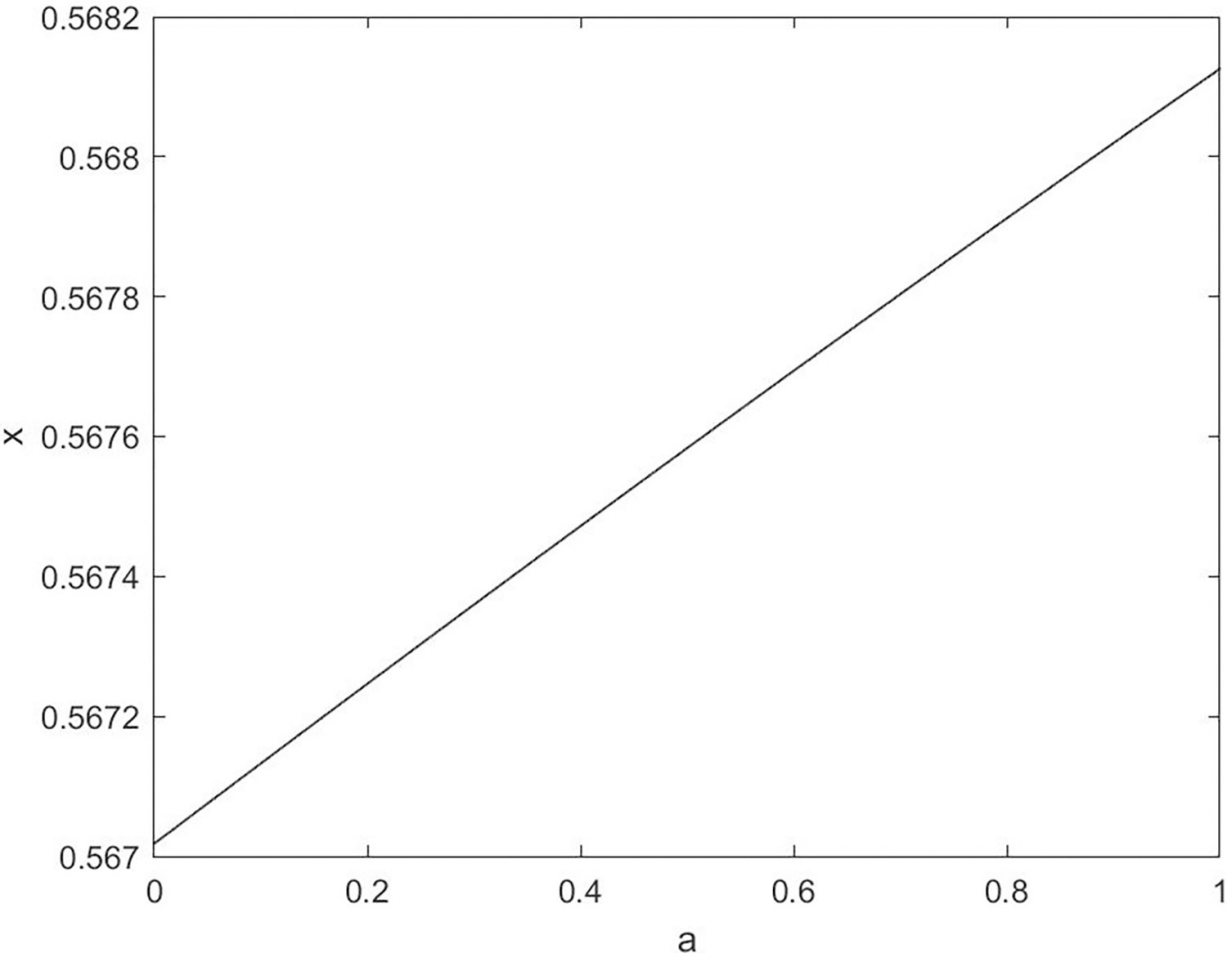
Bifurcation diagram of LSS for *β* = 0.5.

**Fig 2:**
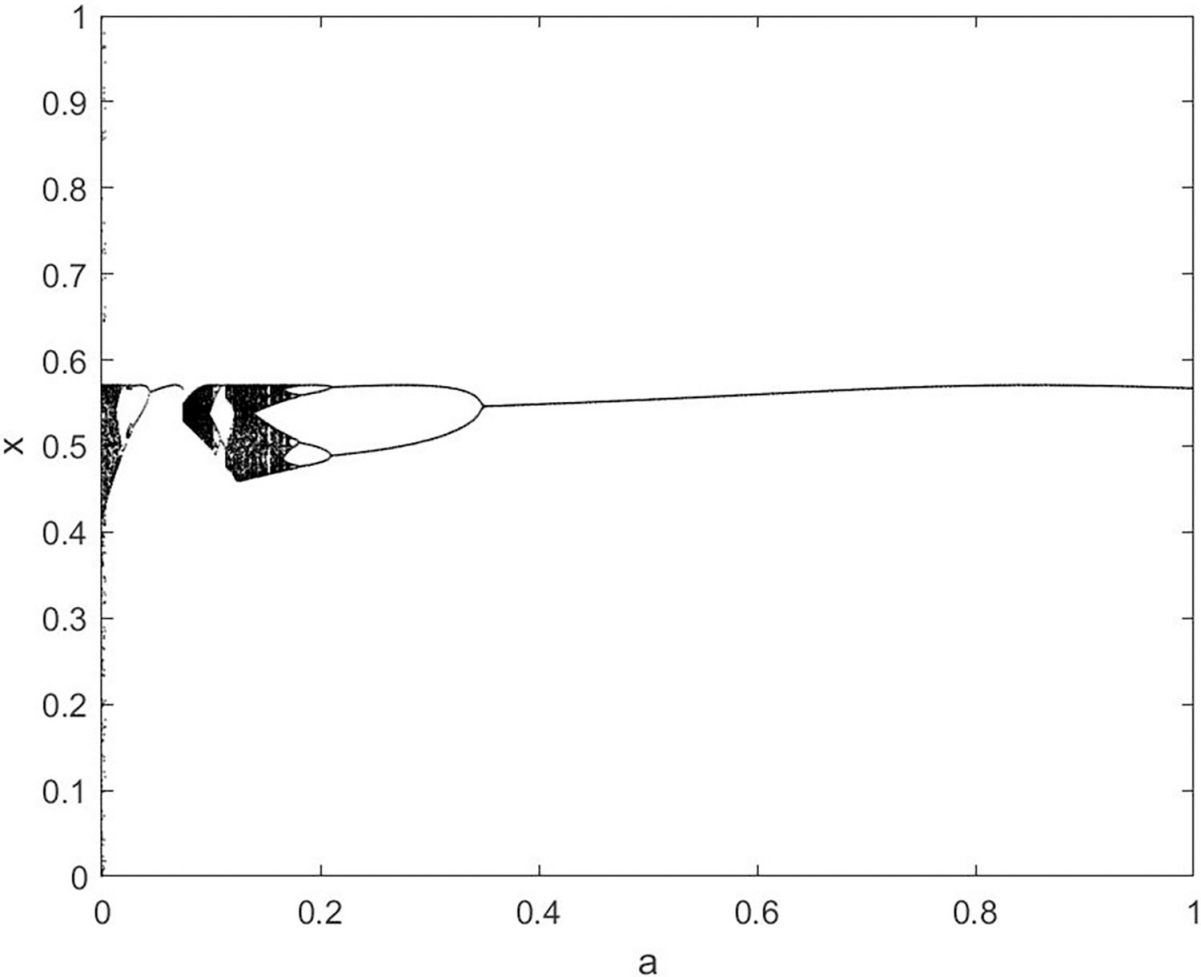
Bifurcation diagram of STS for *β* = 0.5.

**Fig 3:**
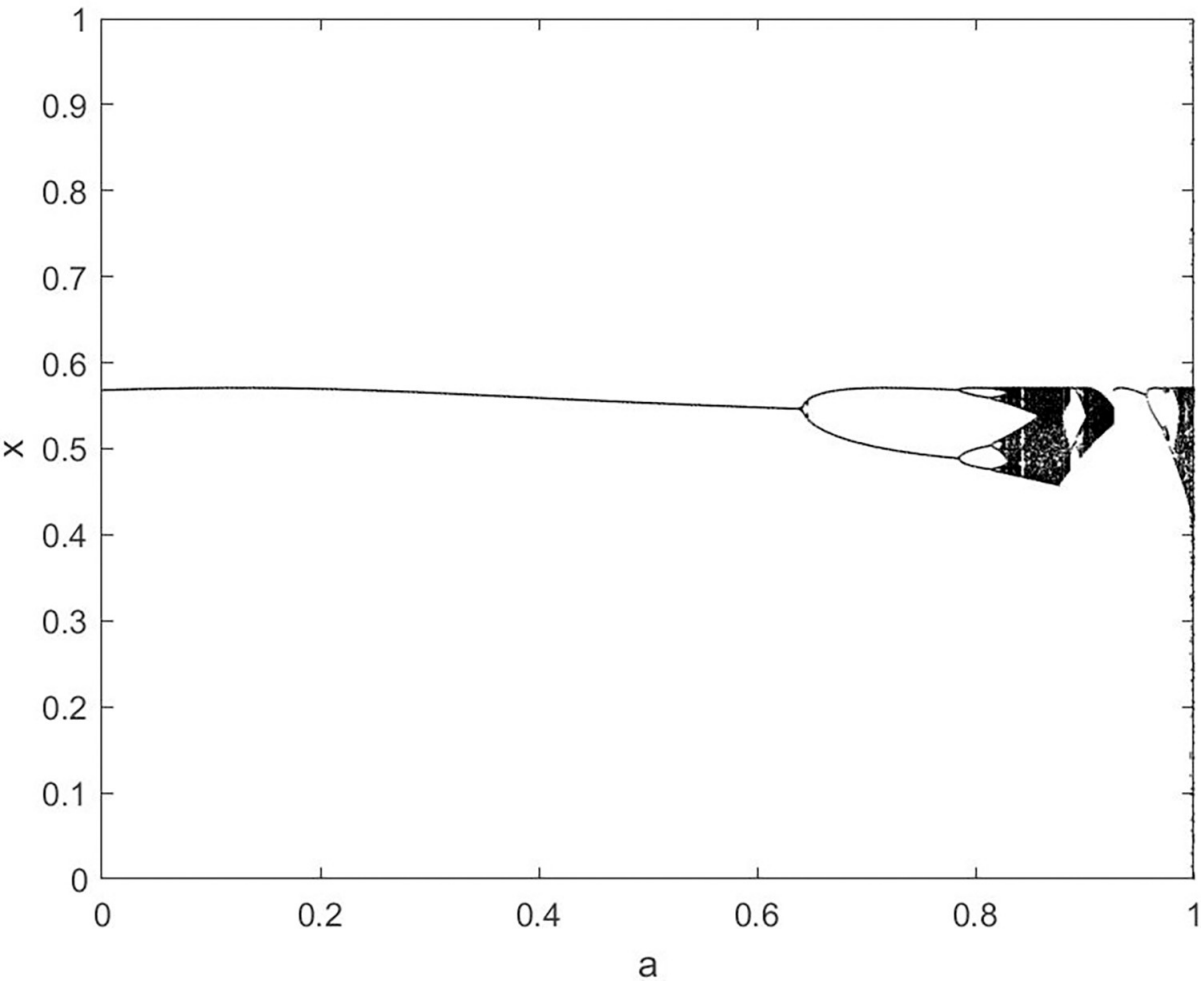
Bifurcation diagram of TLS for *β* = 0.5.

**Fig 4:**
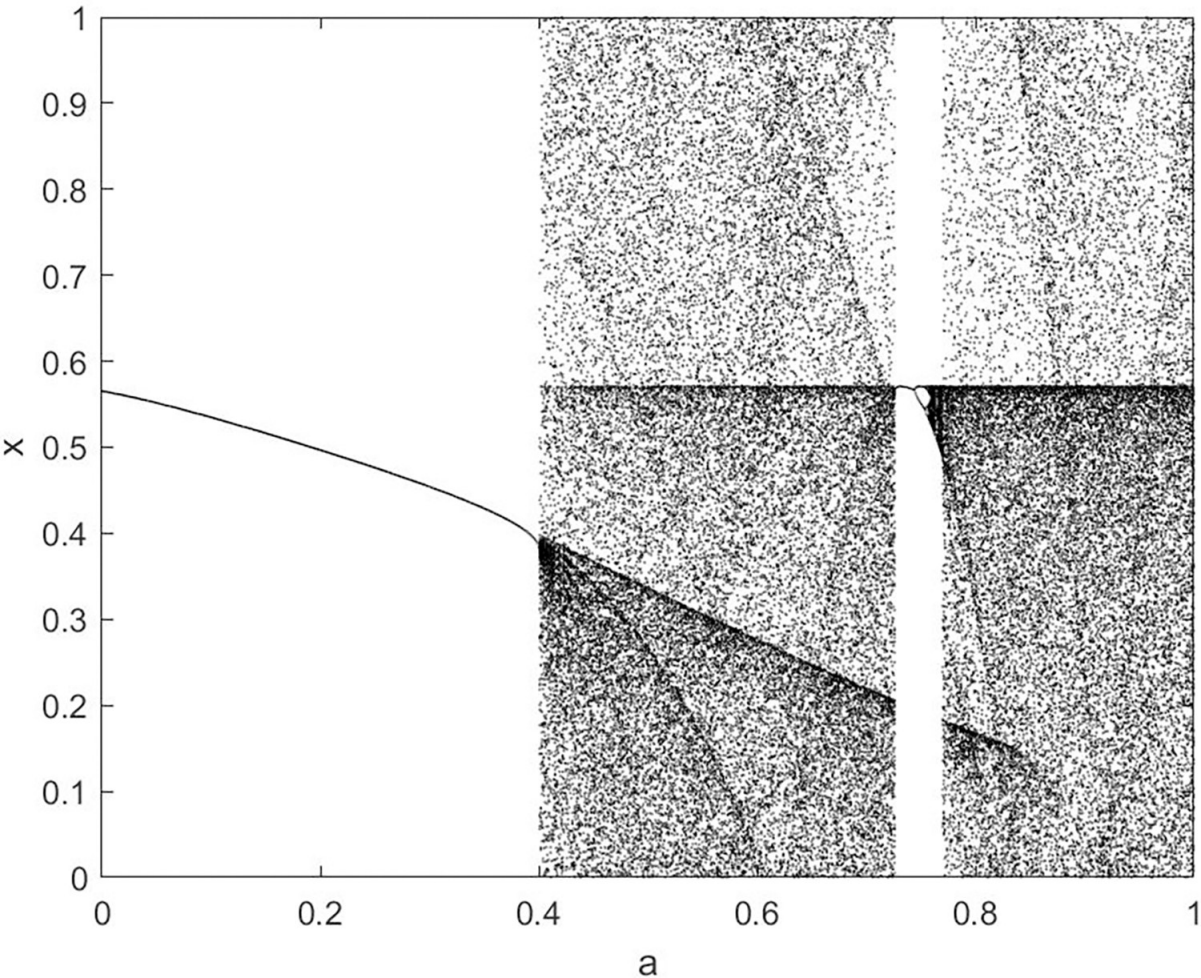
Bifurcation diagram of LCS for *β* = 0.5.

**Fig 5:**
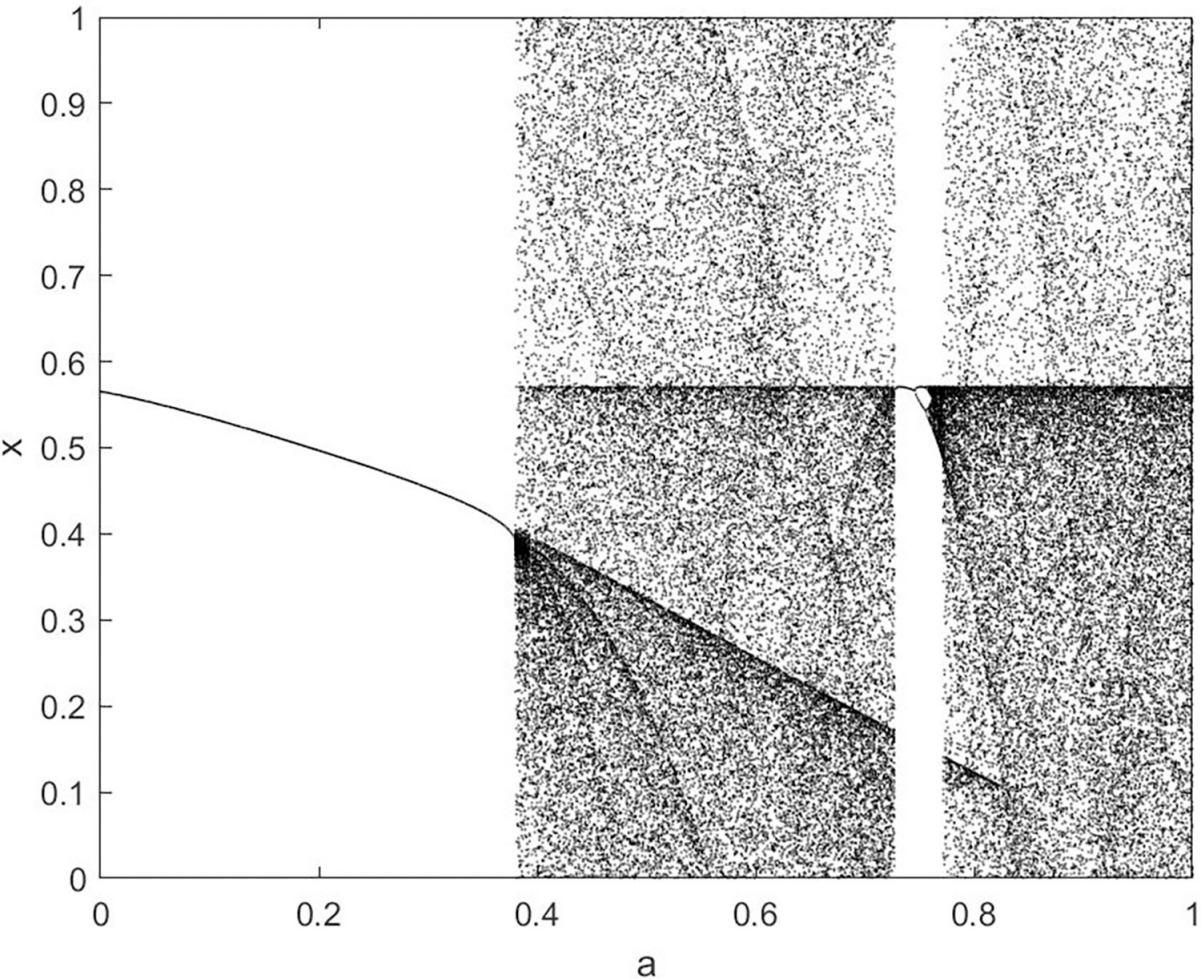
Bifurcation diagram of SCS for *β* = 0.5.

**Fig 6:**
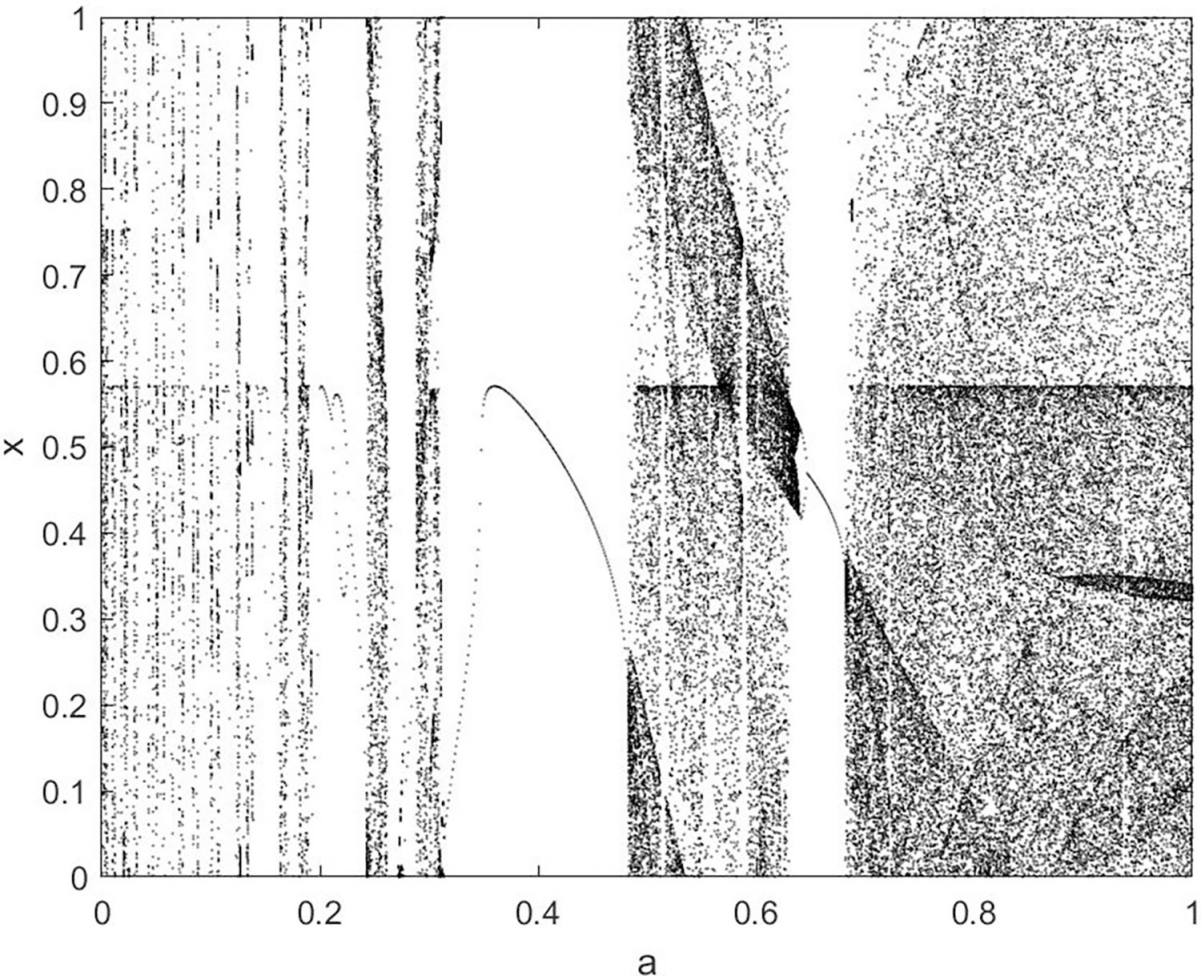
Bifurcation diagram of TCS for *β* = 0.5.

**Fig 7:**
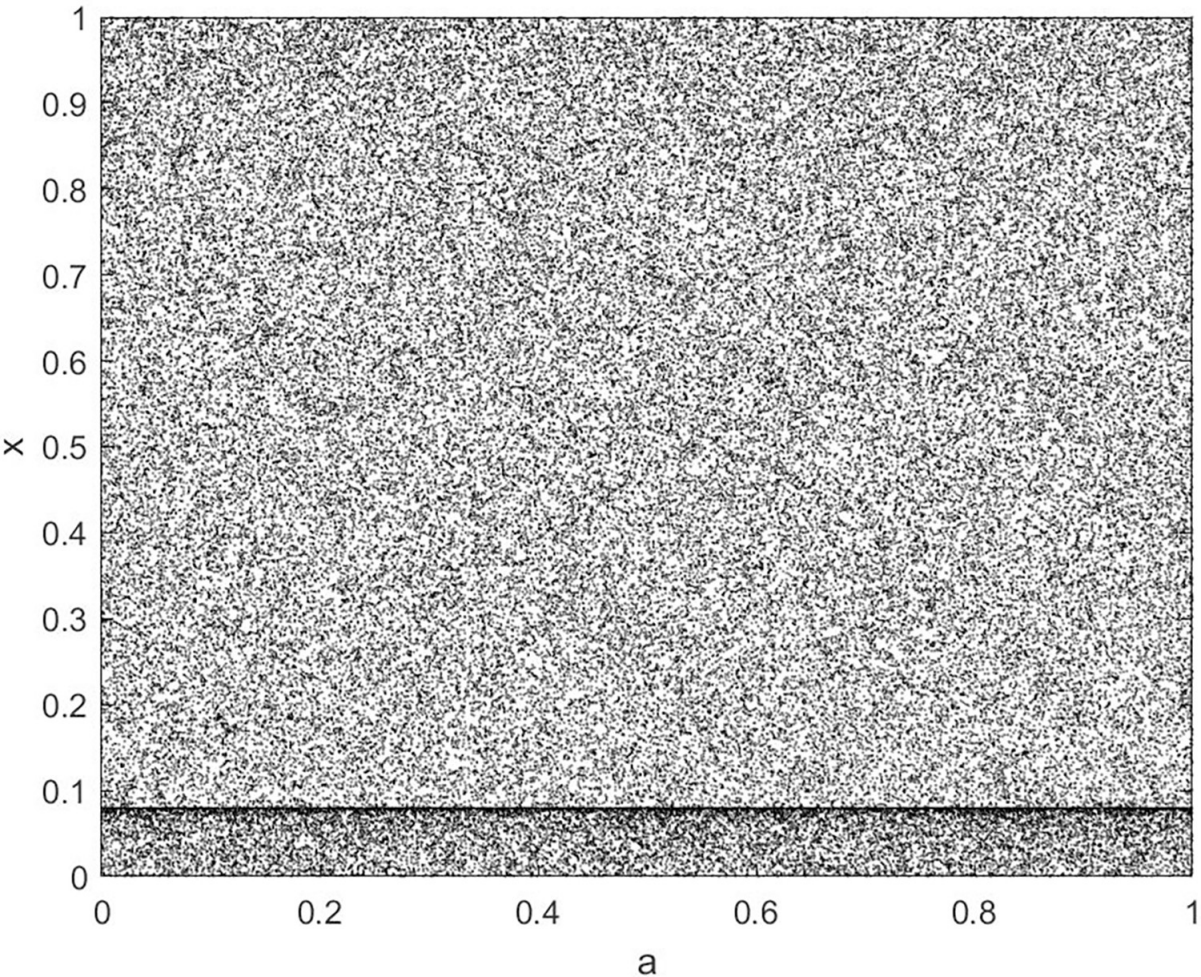
Bifurcation diagram of LSS for *β* = 50.

**Fig 8:**
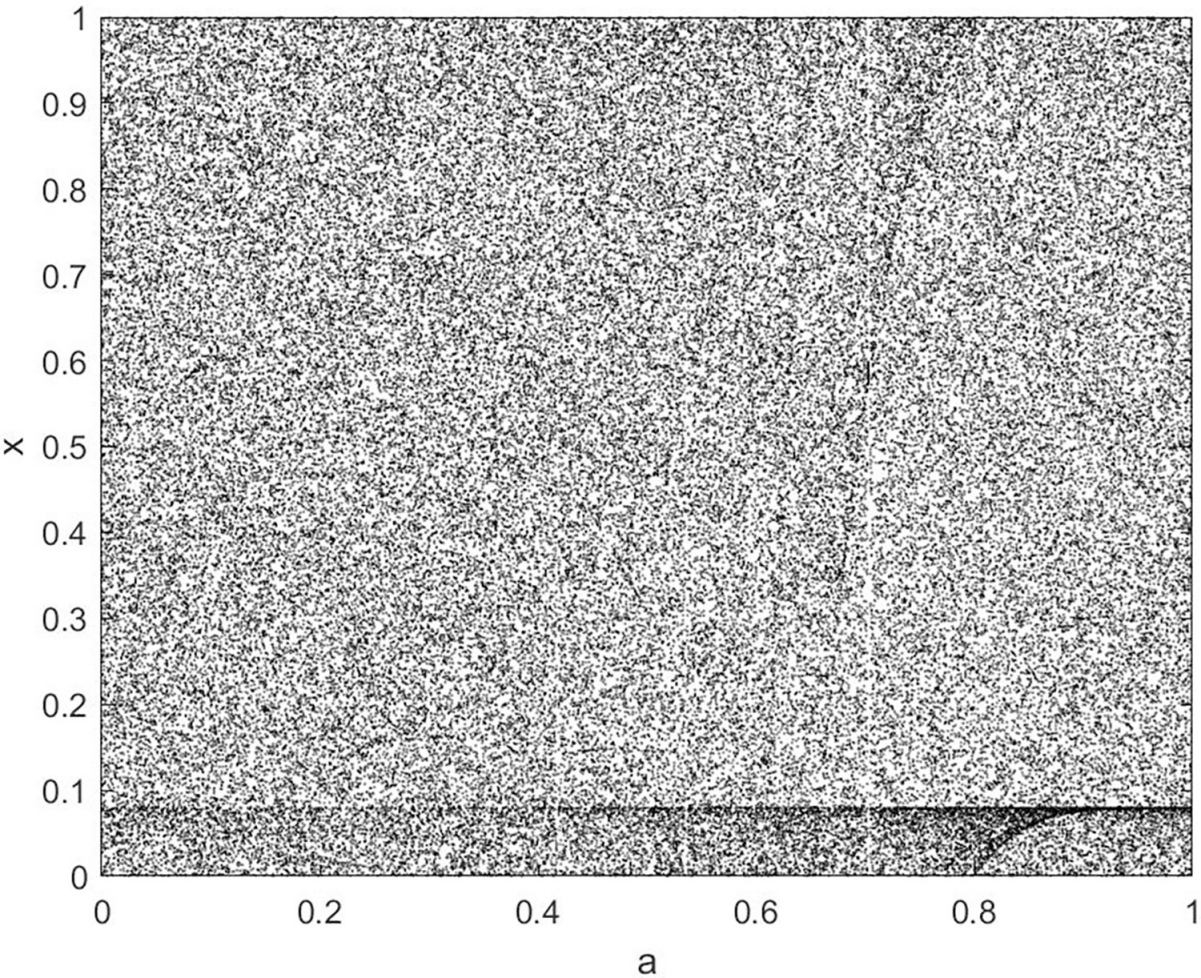
Bifurcation diagram of STS for *β* = 50.

**Fig 9:**
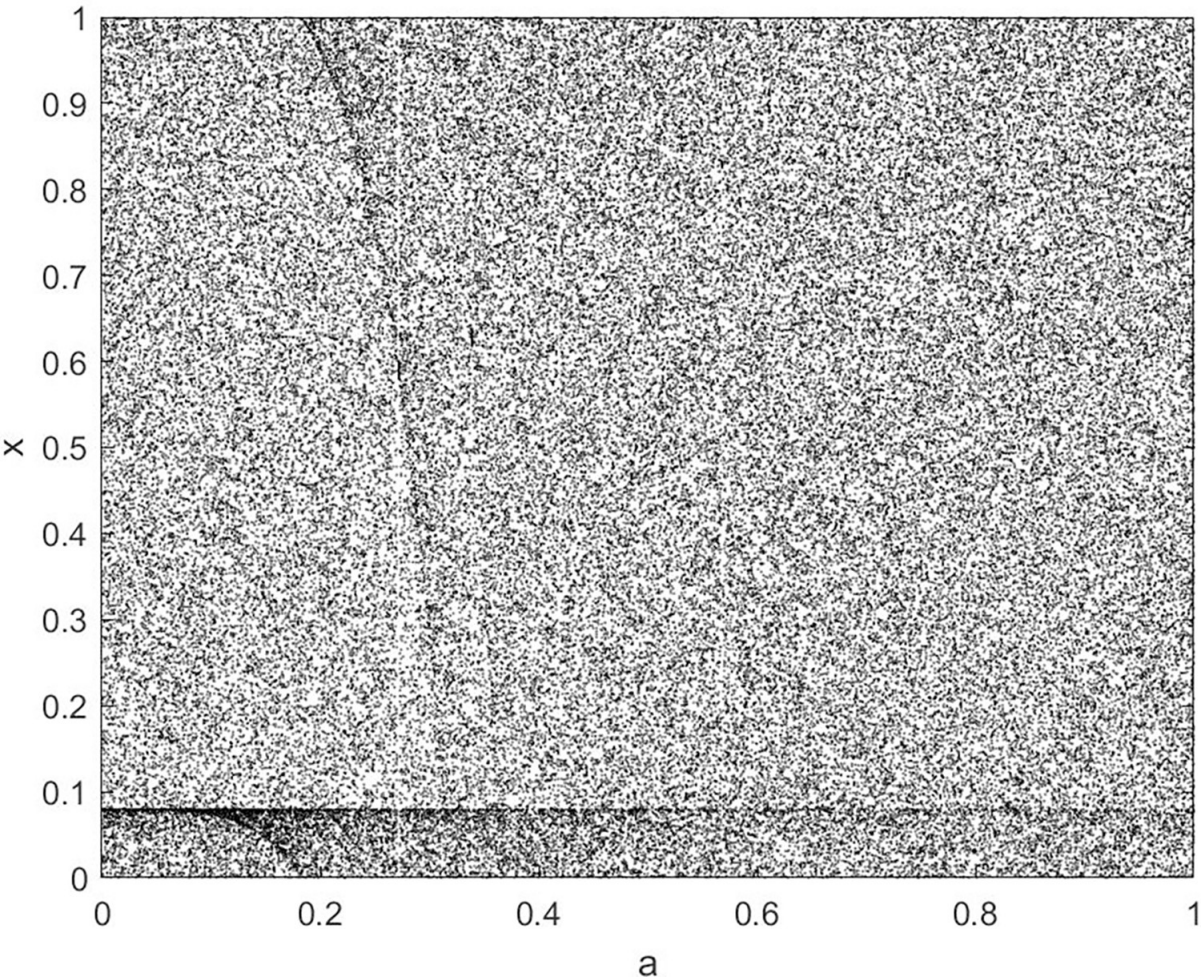
Bifurcation diagram of TLS for *β* = 50.

**Fig 10:**
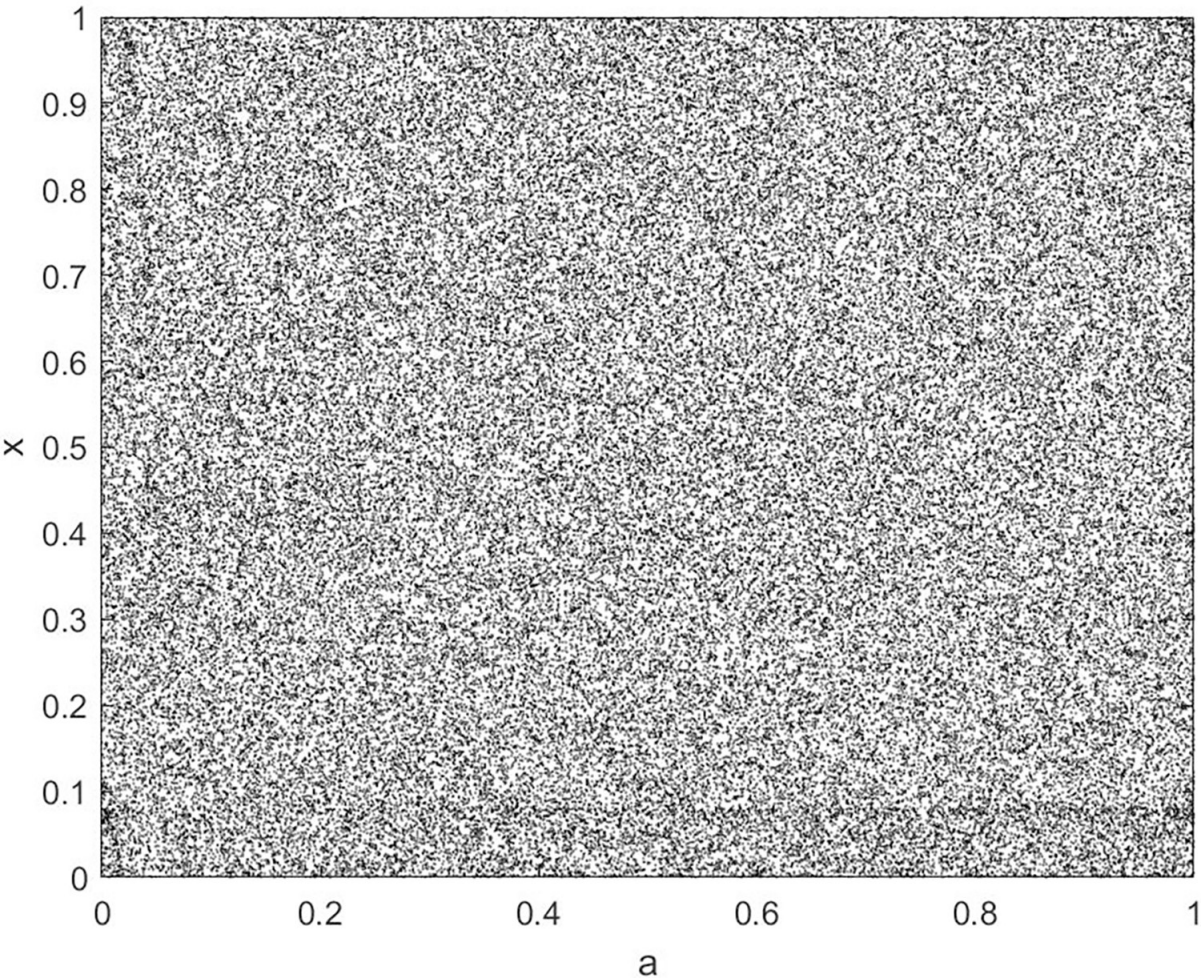
Bifurcation diagram of LCS for *β* = 50.

**Fig 11:**
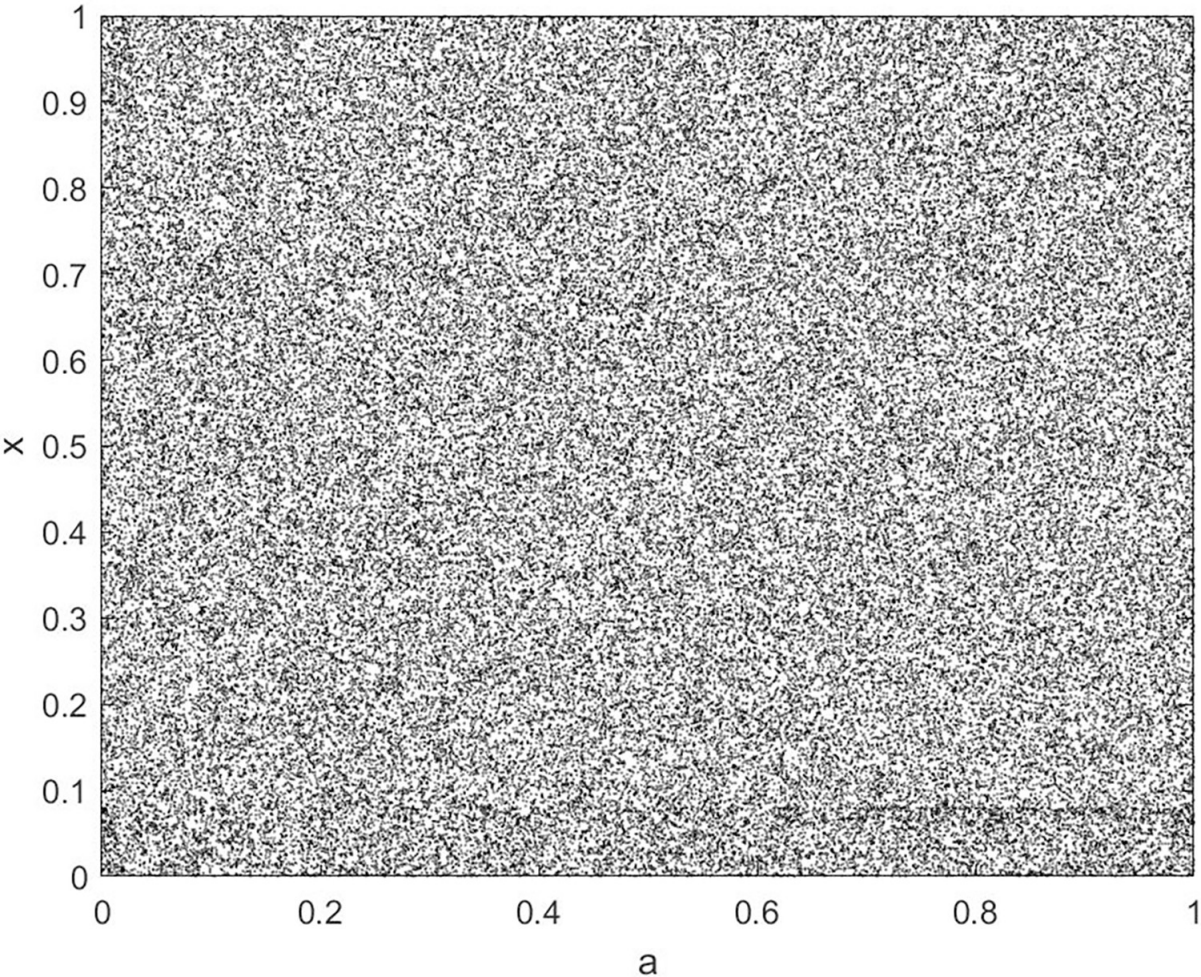
Bifurcation diagram of SCS for *β* = 50.

**Fig 12:**
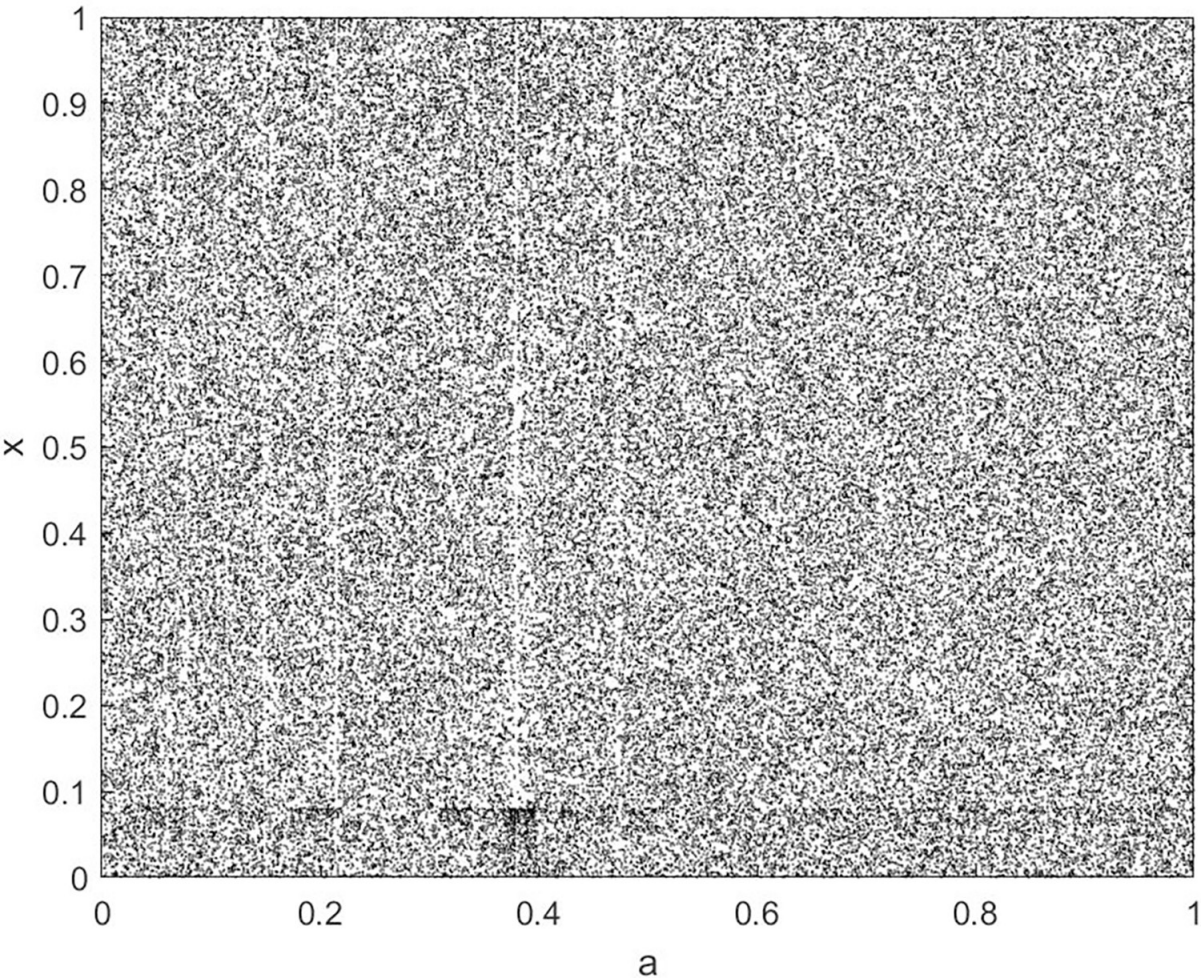
Bifurcation diagram of TCS for *β* = 50.

**Fig 13:**
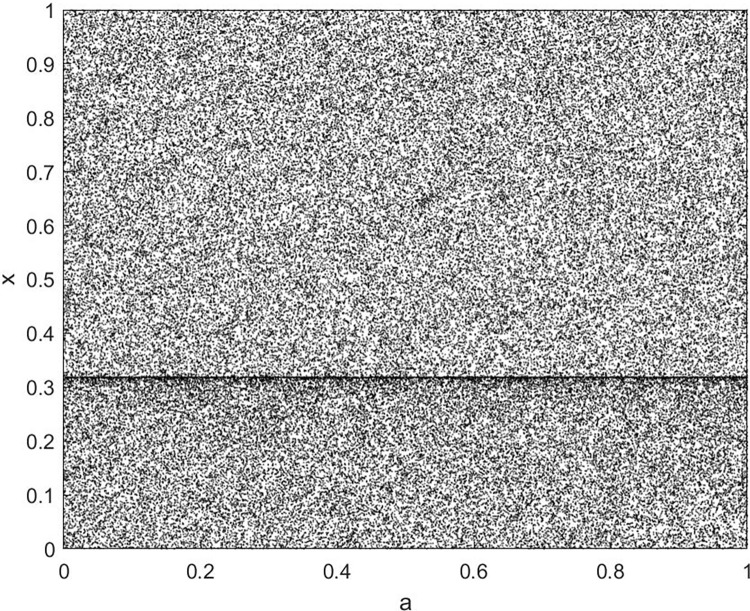
Bifurcation diagram of LSS for *β* = 200.

**Fig 14:**
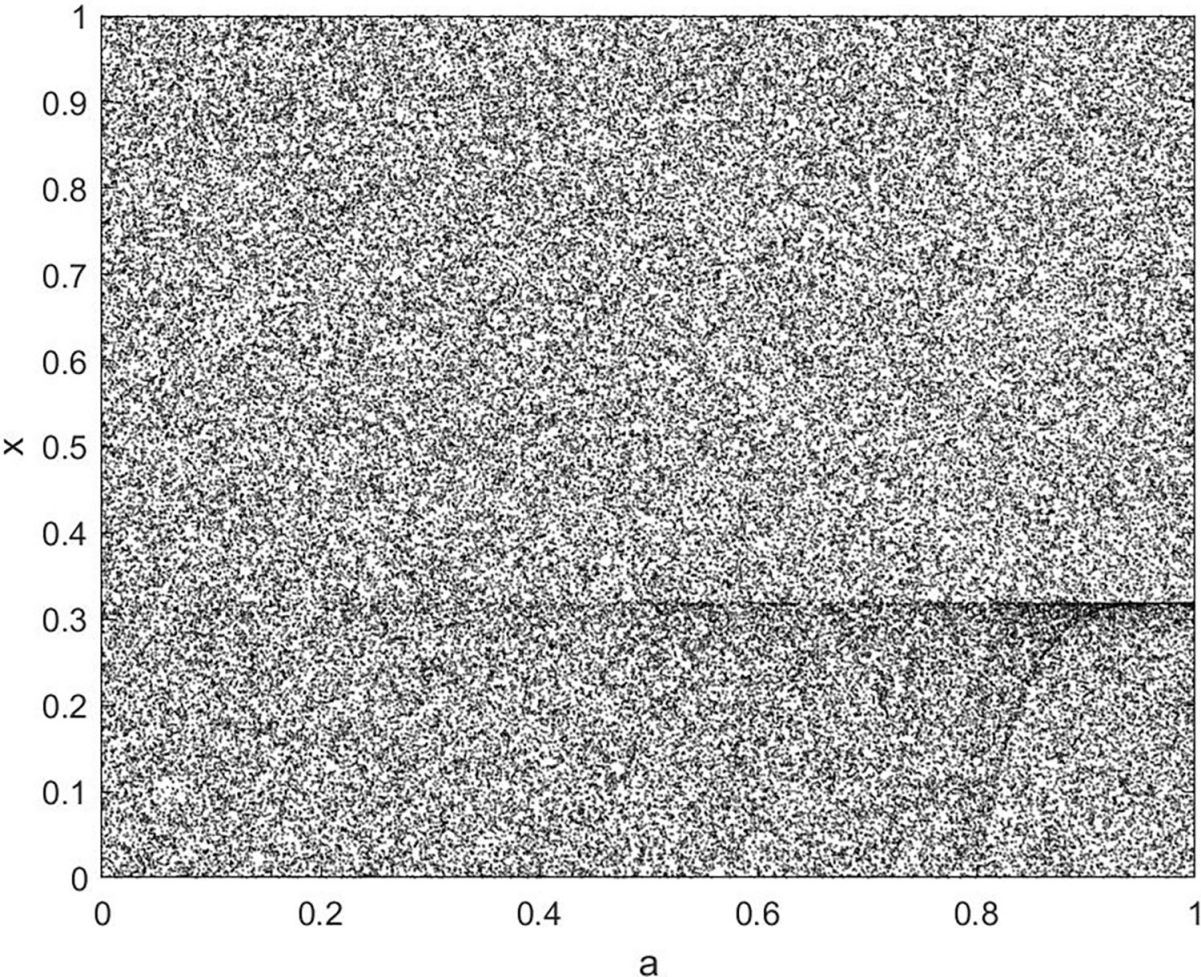
Bifurcation diagram of STS for *β* = 200.

**Fig 15:**
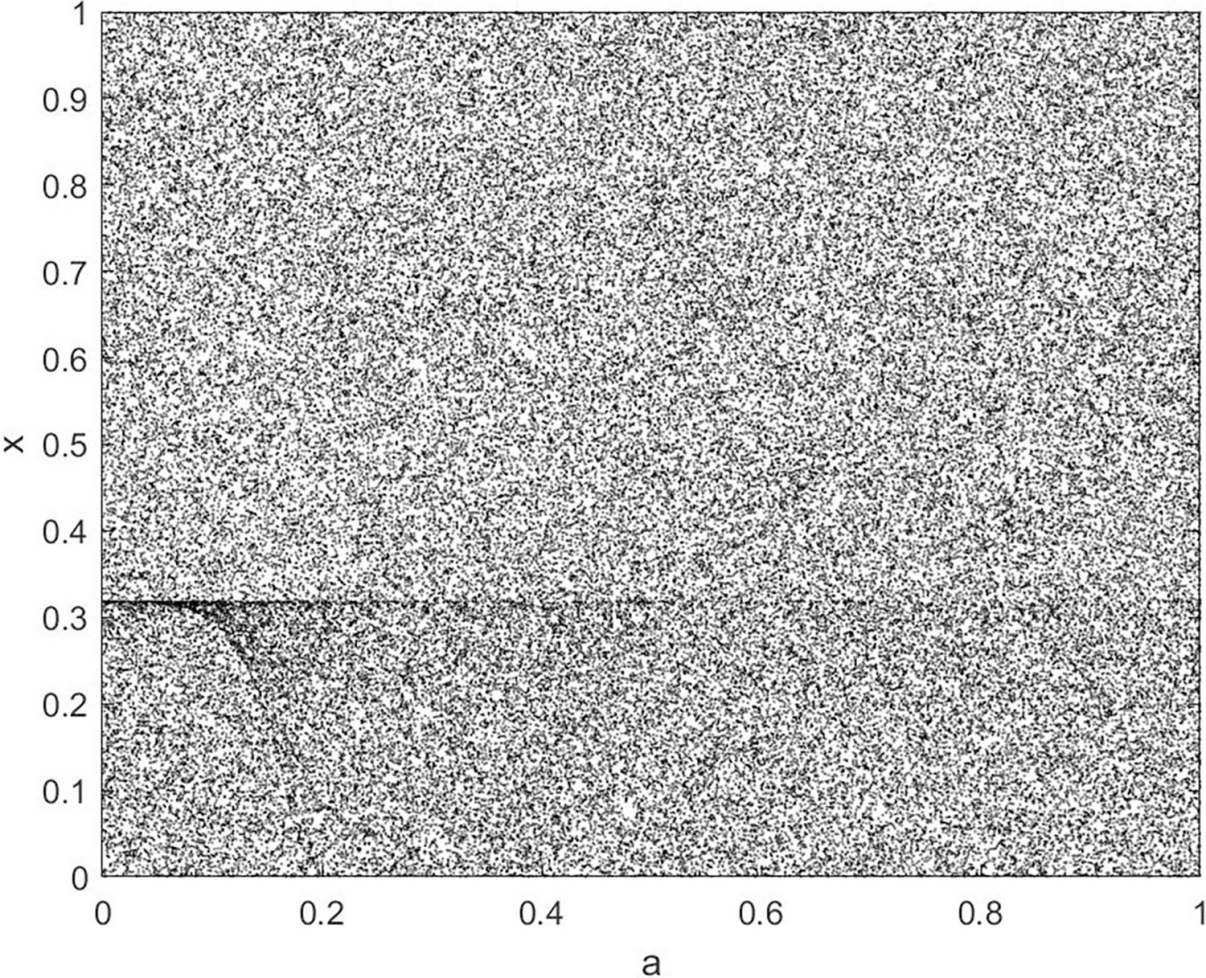
Bifurcation diagram of TLS for *β* = 200.

**Fig 16:**
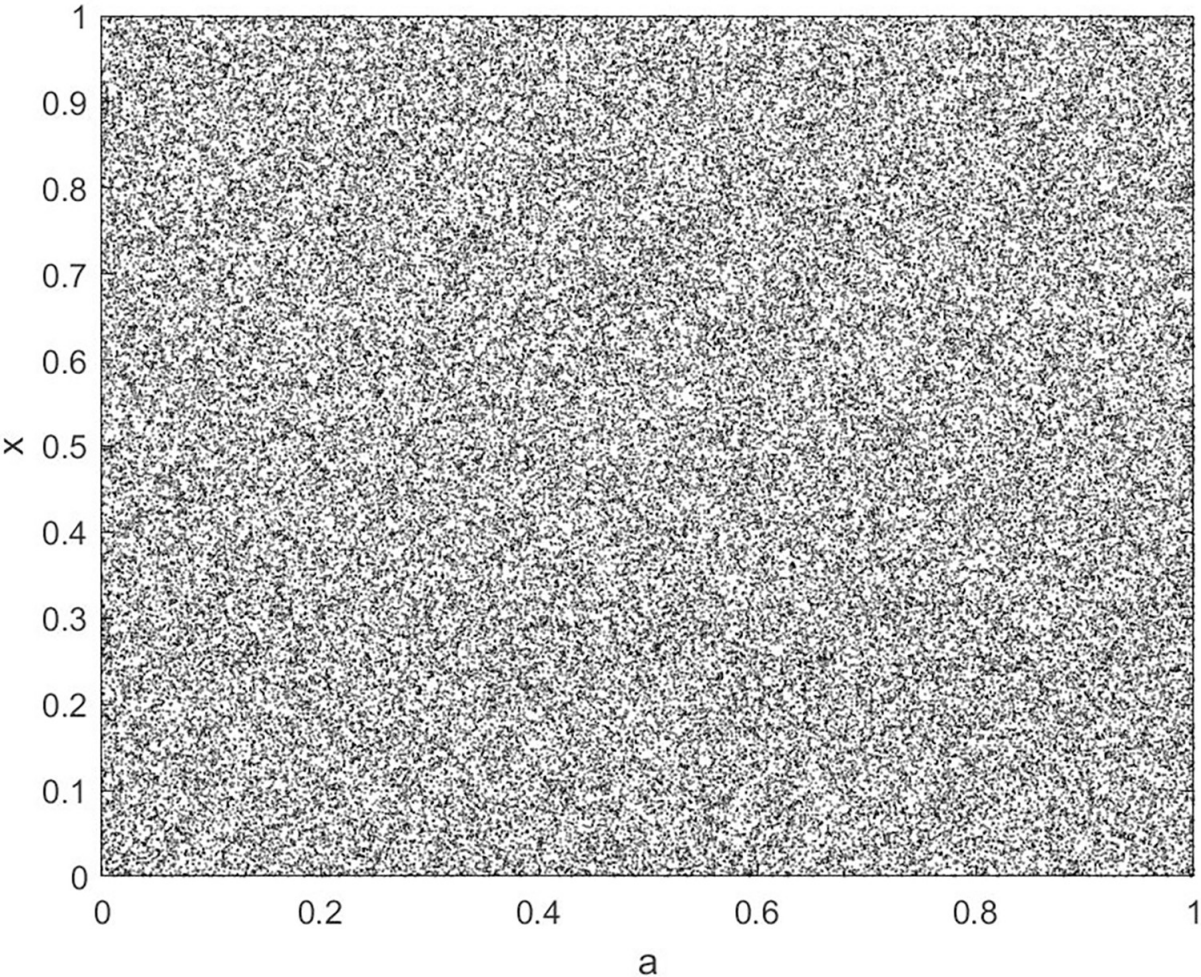
Bifurcation diagram of LCS for *β* = 200.

**Fig 17:**
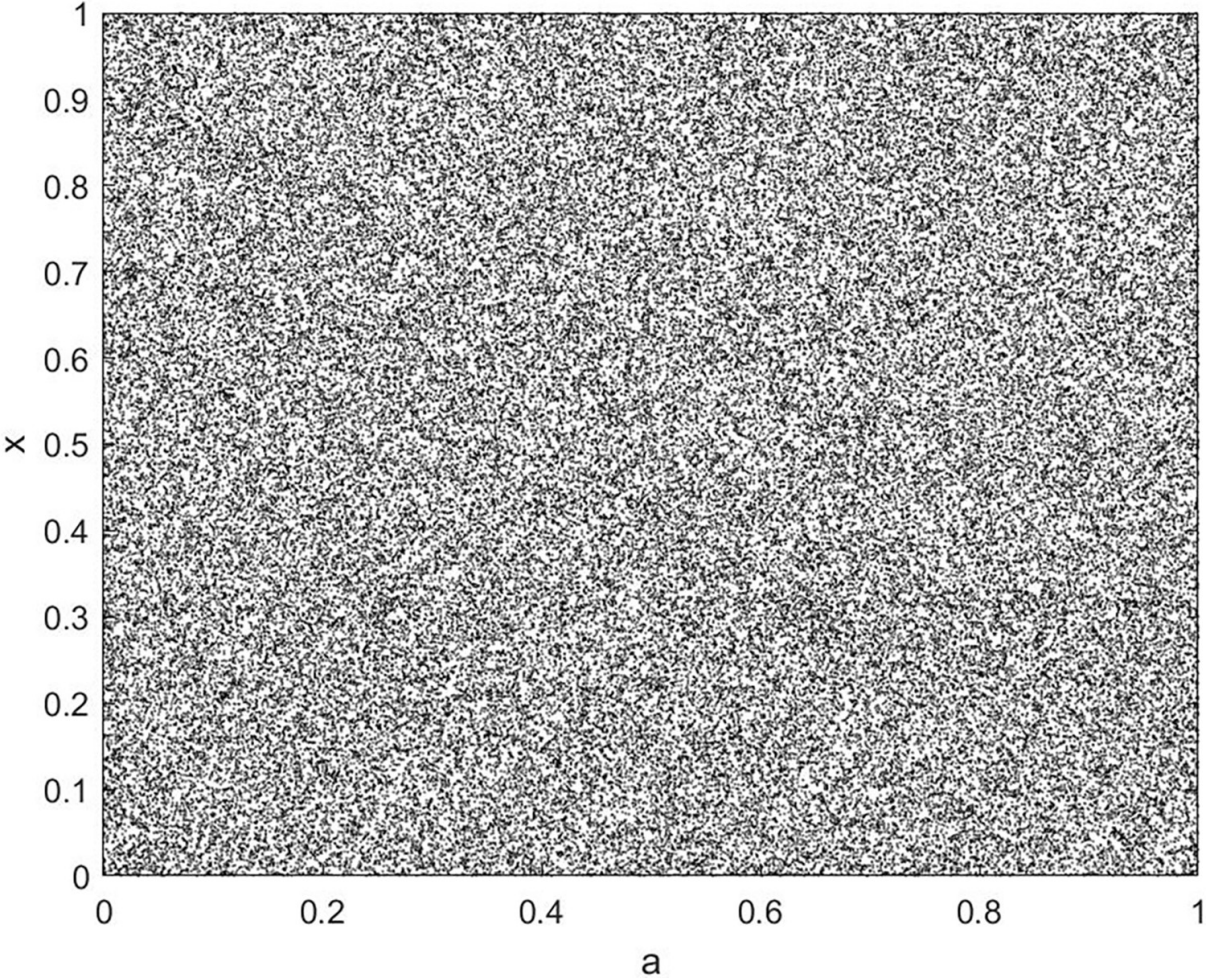
Bifurcation diagram of SCS for *β* = 200.

**Fig 18:**
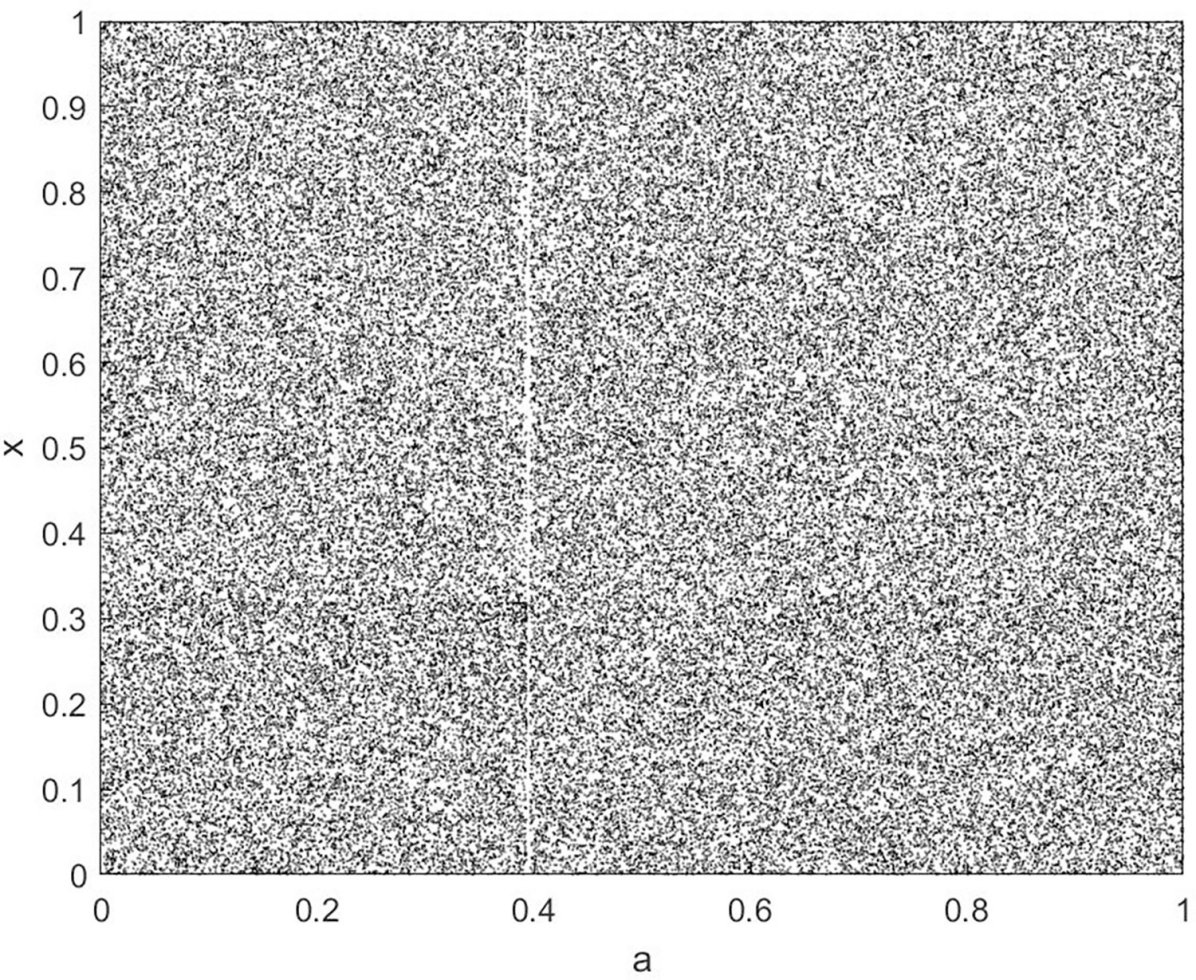
Bifurcation diagram of TCS for *β* = 200.

In Fig 1, when the control parameter β is small, such as *β* = 0.5, a small part of the entire data range can be randomly covered by the output of these bifurcation graphs. In Fig 2, when *β* takes a slightly larger value, such as *β* = 50, the entire data range can basically be randomly covered by the output of these bifurcation graphs. But there are *a* small number of output values that are relatively concentrated. In Fig 3, when β takes a large value, such as *β* = 200, the entire data range can basically be randomly covered by the output of these bifurcation graphs. The concentrated output values become smaller. The most scattered output values are Fig 3 (d) LCS and (e) SCS. These two images are basically flawless. Therefore, the encryption algorithm in this chapter uses the chaotic image generated by these two values as the encryption password.

#### 2.3.2 Lyapunov index

Lyapunov exponent is an important mathematical tool for describing the behavior of chaotic systems. It is used to measure the exponential growth rate between adjacent trajectories in dynamic systems [16]. The Lyapunov index can help us determine whether a system has chaotic behavior and how high the degree of chaos is. A Lyapunov exponent greater than 0 indicates that the system is highly sensitive to small changes in initial conditions, which is a typical feature of chaotic phenomena. The Lyapunov calculation formula is shown in Eq (12).

$$\lambda =\underset{N\to \infty }{\lim}\frac{1}{N}\sum_{n=1}^{N}\ln|f^{\prime }(x_{n})|$$ (12)

where | *f ’* (*x*_*n*_) | is the absolute value of the derivative of the mapping function at point *x*_*n*_, and *N* is the number of iterations. A positive Lyapunov index indicates that the system is chaotic and extremely sensitive to initial conditions.

**Fig 19:**
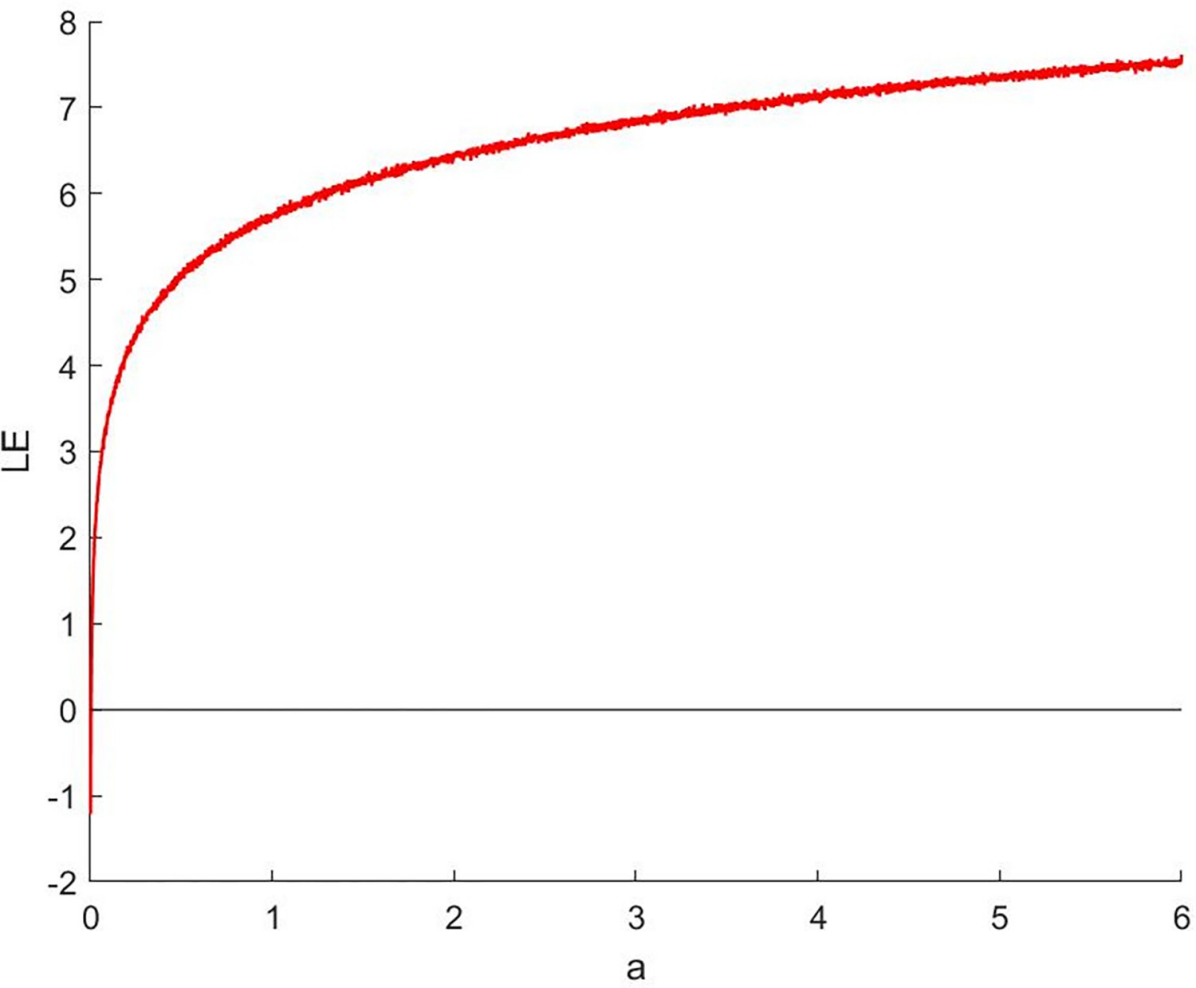
Lyapunov index of LCS.

**Fig 20:**
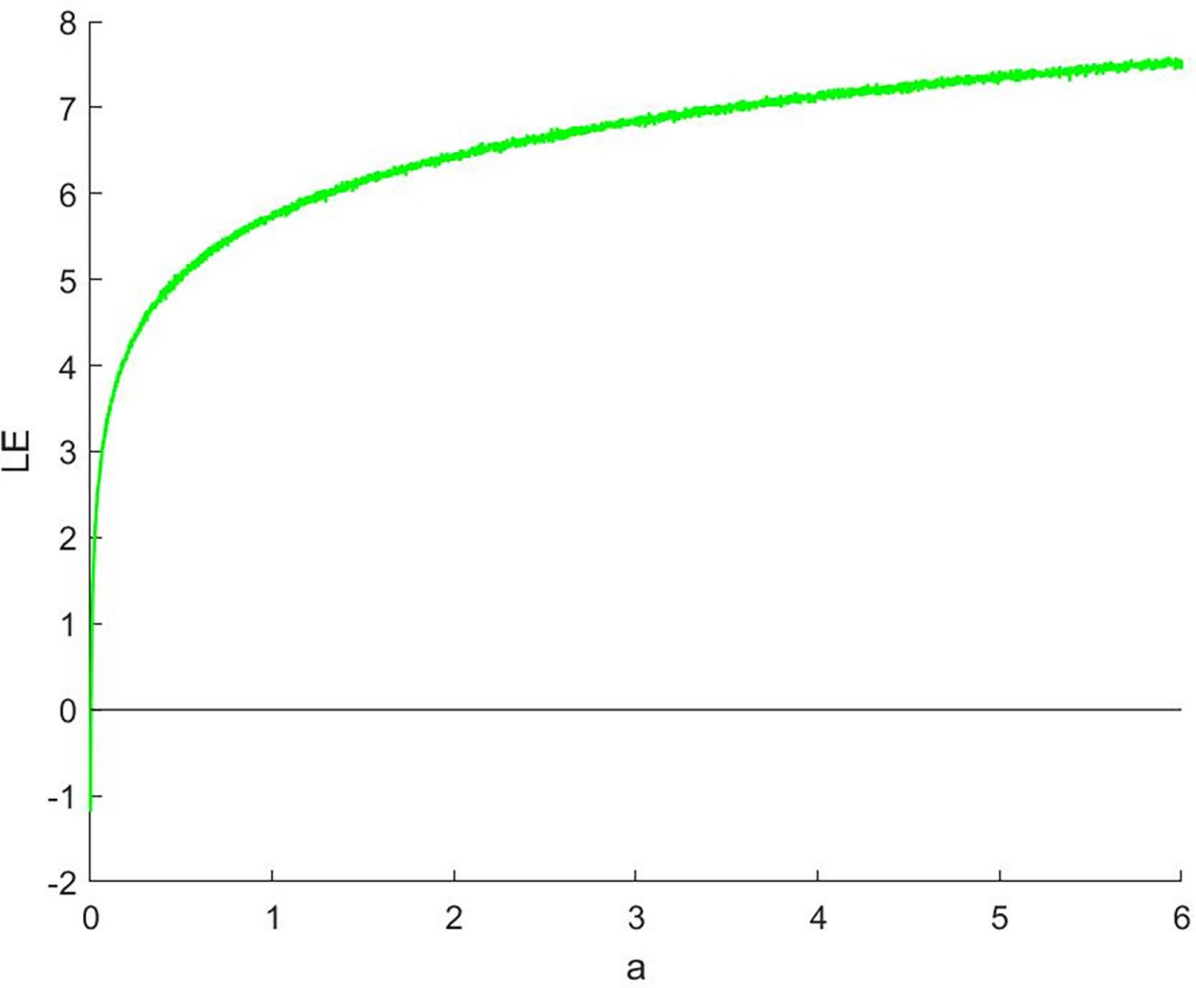
Lyapunov index of SCS.

Analyze the Lyapunov exponent values of (9) LCS and (10) SCS. When *β* = 200, *θ* = 0.5, the Lyapunov exponent value is shown in Figs 19 and 20 are the Lyapunov exponent graphs of LCS and SCS respectively. All Lyapunov exponent values are greater than 0, satisfying the characteristics of chaos.

#### 2.3.3 NIST SP 800–22 randomness test

NIST SP 800–22 is a technical report published by the National Institute of Standards and Technology (NIST). The full name is "A Statistical Test Suite for Random and Pseudo-Random Number Generators for Cryptographical Applications". NIST SP 800–22 includes a series of statistical tests for evaluating the randomness and statistical properties of generated random number sequences. These tests cover a variety of different types of randomness detection, including uniformity, independence, frequency distribution, sequence, uniform distribution, long periodicity, etc. The results of the test can help determine whether the random number generator is secure and random enough [23]. All tests of NIST SP 800–22 are greater than 0.01, indicating that the randomness meets the standard. Based on the values of each key parameter in Section 2.2, draw a conclusion whether randomness is met. Table 1 shows the randomness detection values of LCS and SCS when *β* = 200, *θ* = 0.5, *a* = 0.5. It can be seen that all detection values P are greater than 0.01, indicating that the randomness reaches the standard.

**Table 1:** The randomness detection value of LCS and SCS.

Sub-tests	P of LCS	P of SCS	Results
**frequency**	0.0801	0.5646	pass
**Block Frequency**	0.1149	0.1518	pass
**Run**	0.6420	0.5082	pass
**Longest Run**	0.6911	0.7318	pass
**binary matrix**	0.7286	0.0687	pass
**Discrete Fourier**	0.7456	0.1048	pass
**Non-Overlapping Template**	0.8616	0.3446	pass
**Overlapping Template**	0.9565	0.7882	pass
**Maurer general statistics**	0.2649	0.0832	pass
**Linear Complexity**	0.1688	0.6324	pass
**Seriale** *	0.5518	0.6969	pass
**Approximate Entropy**	0.8460	0.6762	pass
**Cumulative Sums** *	0.9716	0.7735	pass
**Random Excursions** *	0.3204	0.3344	pass
**Random Excursions Variant** *	0.6868	0.6211	pass

*The average values of multiple tests

## 3 Image sector fast encryption and decryption algorithm based on LCS and SCS composite chaos

**Fig 21:**
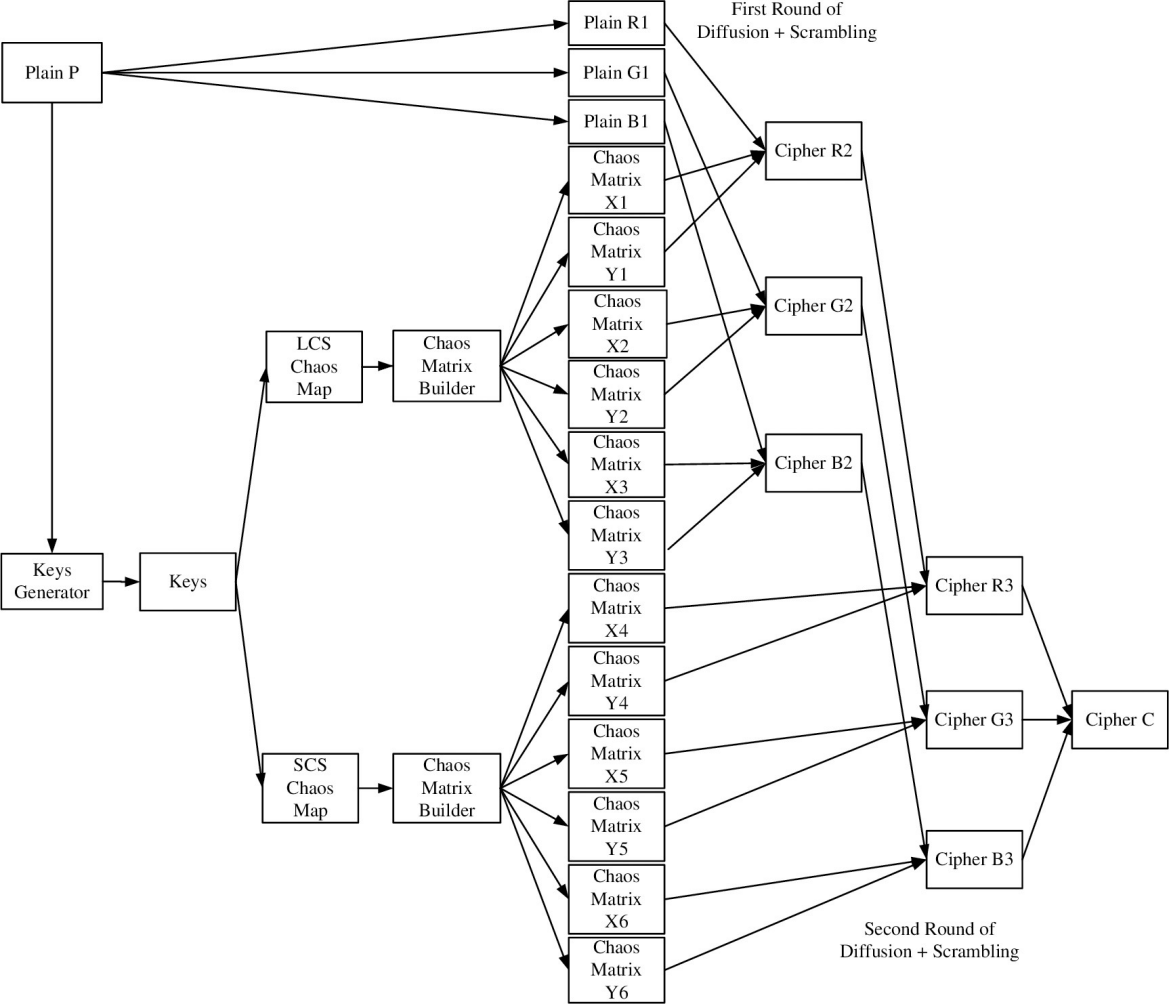
Image encryption algorithm flow chart.

The overall flow chart of the image encryption algorithm designed in this chapter is shown in Fig 21. The encryption process is generally divided into three processes. First, generate the key. Second, the chaos matrix password is generated according to the LCS and SCS chaos formulas). Third, image sector encryption algorithm with two rounds of diffusion and scrambling simultaneously.

### 3.1 Key generator

Through the analysis in Section 2.3, this paper uses the chaos mapping of LCS and SCS with better performance, namely Eqs (10) and (11) to generate the chaos matrix. In order to distinguish each parameter and facilitate image encryption, these two formulas are rewritten, as shown in Eqs (13) and (14).


$$x_{n+1}=|\beta_{1}\pi \;\sin\{\pi [4a_{1}x_{n}(1-x_{n})+|4x_{n}^{3}-3(1-a_{1})x_{n}|+\theta_{1}]\}|$$ (13)

$$x_{n+1}=|\beta_{2}\pi \;\sin\{\pi [a_{2}\;\sin(\pi x_{n})+|4x_{n}^{3}-3(1-a_{2})x_{n}|+\theta_{2}]\}|$$ (14)

The corresponding keys include a total of 8 keys for the chaos system, including 6 system control parameters *a*_1_ ∈ [0,1], *a*_2_ ∈ [0,1], *β*_1_ > 0, *β*_2_ > 0,*θ*_1_ ∈ [0,1], *θ*_2_ ∈ [0,1] and 2 system initial values *x*_0_ ∈ [0,1]. In order to expand the key space and enhance the ability of the image encryption algorithm to resist attacks, this article uses the SHA-256 function to generate keys. Specific steps are as follows.

Using the SHA-256 function based on the plain color image *P*, the 256-bit hash value *K* can be obtained. Then, *K* is divided into 32 *k*s, each *k* is 8 bits. As shown in Eq (15). 1 ≤ *u* ≤ 32.


$$K=k_{1}k_{2}\ldots k_{32},k_{u}=k_{u1}k_{u2}\ldots k_{u8}$$ (15)

Calculate the six system control parameters and *K*, as shown in Eqs (16)–(18).


$$\{\begin{array}{c} a_{1}=(a_{1}+0.001\times (k_{1}⊕k_{2}))\mathrm{mod}\;1\\ a_{2}=(a_{2}+0.001\times (k_{3}⊕k_{4}))\mathrm{mod}\;1 \end{array}$$ (16)

$$\{\begin{array}{c} \beta_{1}=(\beta_{1}+0.001\times (k_{5}⊕k_{6}))\mathrm{mod}\;1\\ \beta_{2}=(\beta_{2}+0.001\times (k_{7}⊕k_{8}))\mathrm{mod}\;1 \end{array}$$ (17)

$$\{\begin{array}{c} \theta_{1}=(\theta_{1}+0.001\times (k_{9}⊕k_{10}))\mathrm{mod}\;1\\ \theta_{2}=(\theta_{2}+0.001\times (k_{11}⊕k_{12}))\mathrm{mod}\;1 \end{array}$$ (18)

The purpose of multiplying by 0.001 is to produce small changes in each control parameter. The purpose of the mod operation is to keep the control parameters within their respective value ranges. ⊕ is the XOR operation.

(3) Calculate 12 system initial values *x*_*i*_ ∈ [0,1], *i* = 1, 2,…, 12. and the specific values of a based on *k*_*i*_ and plain *P*. The specific method is in Section 3.2.

### 3.2 Chaos matrix password generator

This section uses LCS and SCS chaos mapping to generate 6 *m* × *n* pseudo-random matrices each as the chaos matrix password. Among them, *P* is the plain text color image, *m* is the length of *P*, *n* is the width of *P*, and let *m* = *n*. Specific steps are as follows.

(1) Use the parameter values *a*_1_, *a*_2_, *β*_1_, *β*_2_, *θ*_1_, *θ*_2_ calculated by Eqs (16)–(18) as the initial values of Eqs (13) and (14).

(2) In the plain text color image *P*, three images of red, green, and blue are separated according to the three primary colors principle, called *R*1, *G*1, and *B*1.

(3) Calculate *i*_1_, *i*_2_, *i*_3_, through Eq (19), and calculate *j*_1_, *j*_2_, *j*_3_, through Eq (20).

$$\{\begin{array}{c} i_{1}=(k_{13}⊕k_{14})\mathrm{mod}\;m\\ i_{2}=(k_{15}⊕k_{16})\mathrm{mod}\;m\\ i_{3}=(k_{17}⊕k_{18})\mathrm{mod}\;m \end{array}$$ (19)

$$\{\begin{array}{c} j_{1}=(k_{19}⊕k_{20})\mathrm{mod}\;n\\ j_{2}=(k_{21}⊕k_{22})\mathrm{mod}\;n\\ j_{3}=(k_{23}⊕k_{24})\mathrm{mod}\;n \end{array}$$ (20)

where *i*_1_, *i*_2_, *i*_3_, *j*_1_, *j*_2_, *j*_3_ are used as the row number or column number of a pixel in *R*1, *G*1, *B*1.

(4)According to Eq (21), take out each value in *R*1, *G*1, and *B*1 respectively.


$$\{\begin{array}{c} x_{1}=0.001\times R1(i_{1},j_{1})\\ x_{2}=0.001\times G1(i_{2},j_{2})\\ x_{3}=0.001\times B1(i_{3},j_{3})\\ x_{4}=0.001\times R1(i_{1},i_{1})\\ x_{5}=0.001\times G1(i_{2},i_{2})\\ x_{6}=0.001\times B1(i_{3},i_{3})\\ x_{7}=0.001\times R1(j_{1},i_{1})\\ x_{8}=0.001\times G1(j_{2},i_{2})\\ x_{9}=0.001\times B1(j_{3},i_{3})\\ x_{10}=0.001\times R1(j_{1},j_{1})\\ x_{11}=0.001\times G1(j_{2},j_{2})\\ x_{12}=0.001\times B1(j_{3},j_{3}) \end{array}$$ (21)

The purpose of multiplying all *x* values by 0.001 is to keep their values between 0 and 0.255. And select part of the key from the plain to ensure that the encryption key changes with the plain text each time, which can resist many common attacks.

(5) Put {*x*_1_, *a*_1_, *β*_1_, *θ*_1_}, {*x*_2_, *a*_1_, *β*_1_, *θ*_1_}, {*x*_3_, *a*_1_, *β*_1_, *θ*_1_}, {*x*_4_, *a*_1_, *β*_1_, *θ*_1_}, {*x*_5_, *a*_1_, *β*_1_, *θ*_1_}, {*x*_6_, *a*_1_, *β*_1_, *θ*_1_} respectively into Eq (13) and iterate *r* times. *r* = 300, skip the transition form of chaotic mapping. Continue to iterate m × n times to obtain 6 chaos matrix codes {*X*_1_}, {*X*_2_}, {*X*_3_}, {*Y*_1_}, {*Y*_2_}, {*Y*_3_}. Put {*x*_7_, *a*_2_, *β*_2_, *θ*_2_}, {*x*_8_, *a*_2_, *β*_2_, *θ*_2_}, {*x*_9_, *a*_2_, *β*_2_, *θ*_2_}, {*x*_10_, *a*_2_, *β*_2_, *θ*_2_}, {*x*_11_, *a*_2_, *β*_2_, *θ*_2_}, {*x*_12_, *a*_2_, *β*_2_, *θ*_2_} into Eq (14) and iterate *r* times, skip Transition form through chaotic mapping. Continue to iterate *m* × *n* times to obtain 6 chaos matrix codes {*X*_4_}, {*X*_5_}, {*X*_6_}, {*Y*_4_}, {*Y*_5_}, {*Y*_6_}.

### 3.3 Image sector fast encryption algorithm

**Fig 22:**
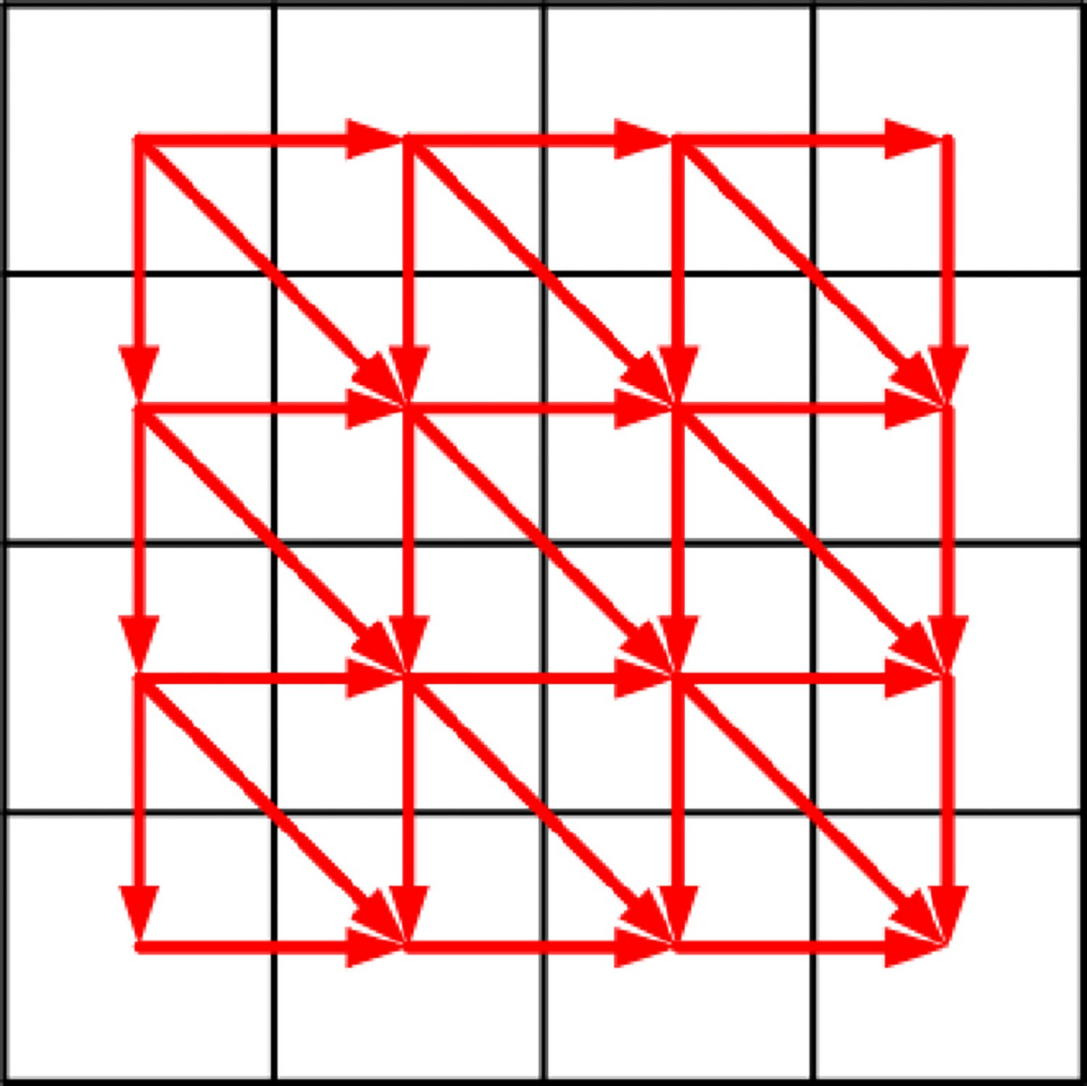
First round of image sector fast encryption algorithm diagram.

**Fig 23:**
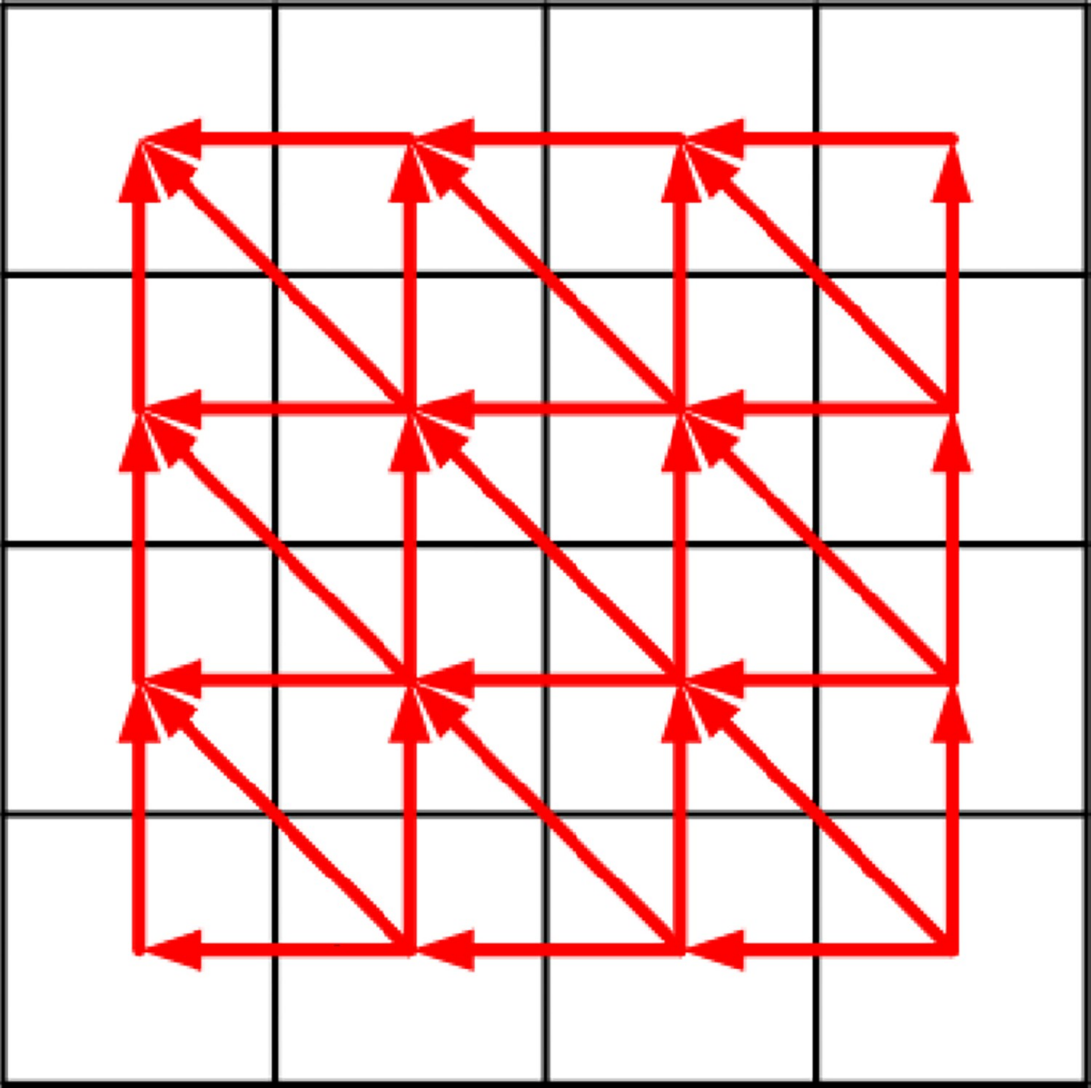
Second round of image sector fast encryption algorithm diagram.

The image sector encryption algorithm based on LCS and SCS chaotic mapping is shown in Figs 22 and 23.

As can be seen in these two figures, the encryption process is like unfolding a traditional Chinese folding fan. Moreover, during the encryption process, diffusion and scrambling are performed alternately at the same time, which improves the encryption speed. Therefore, it is called the image sector fast encryption algorithm. Fig 22 shows the first round of encryption, starting from the upper left corner of the image and ending at the lower right corner, encrypting *R*1, *G*1, *B*1 to *R*2, *G*2, *B*2. Fig 23 shows the second round of encryption, starting from the lower right corner of the image and ending at the upper left corner, encrypting *R*2, *G*2, *B*2 to *R*3, *G*3, *B*3. The chaos matrix {*X*_1_}, {*X*_2_}, {*X*_3_}, {*X*_4_}, {*X*_5_}, {*X*_6_} generated through Section 3.2 is used for diffusion. The generated chaos matrix {*Y*_1_}, {*Y*_2_}, {*Y*_3_}, {*Y*_4_}, {*Y*_5_}, {*Y*_6_} is used for scrambling. Specific steps are as follows.

The first round of encryption starts from the upper left corner. *R*1(1,1) is diffused into *R*2(1,1) through Eq (22).


$$R2(1,1)=(R1(1,1)+X1(1,1)+k_{25})\mathrm{mod}\;m$$ (22)

(2) If it is the first row element of the image, *R*1(1, *j*) is diffused into *R*2(1, *j*) through Eq (23). *j* = 2, 3,…, *n*.


$$R2(1,j)=(R1(1,j)+R1(1,j-1)+X1(1,j)+k_{26})\mathrm{mod}\;m$$ (23)

**Fig 24:**
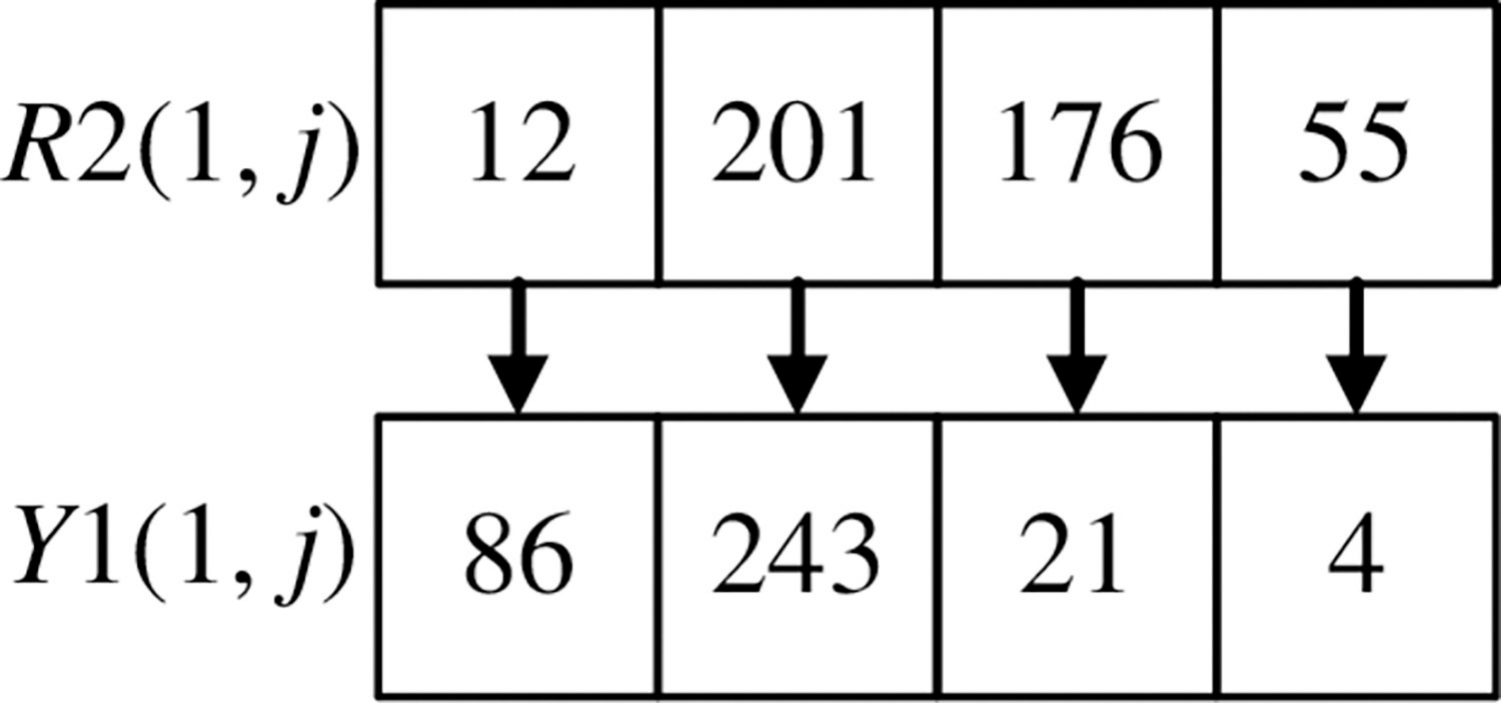
The corresponding relationship between R2(1, j) and Y1(1, j).

**Fig 25:**
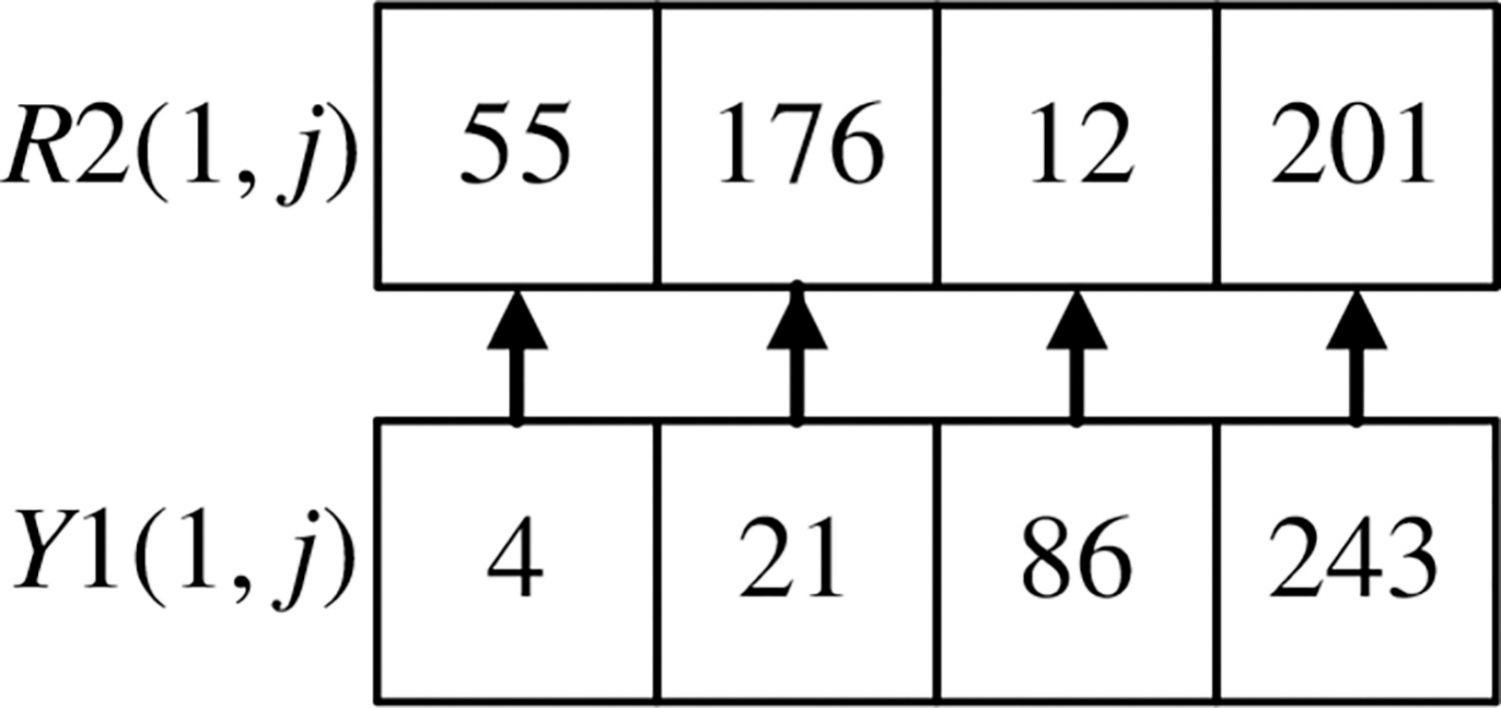
R2(1, j) scrambles the pixels according to Y1(1, j).

The scrambling method of *R*2(1, *j*) is shown in Figs 24 and 25. Record the corresponding relationship between each pixel point at the same position of *R*2(1, *j*) and *Y*1(1, *j*), as shown in Fig 24. Arrange *Y*1(1, *j*) and its first three pixels in ascending order using the selection sort algorithm. Disorganize the pixels of *R*2(1, *j*) according to the position of the corresponding pixel of *Y*1(1, *j*), as shown in Fig 25.

If it is the first column element of the image, *R*1(*i*, 1) is diffused into *R*2(*i*, 1) through Eq (24). *i* = 2, 3,…, *m*.


$$R2(i,1)=(R1(i,1)+R2(i-1,1)+X1(i,1)+k_{27})\mathrm{mod}\;n$$ (24)

**Fig 26:**
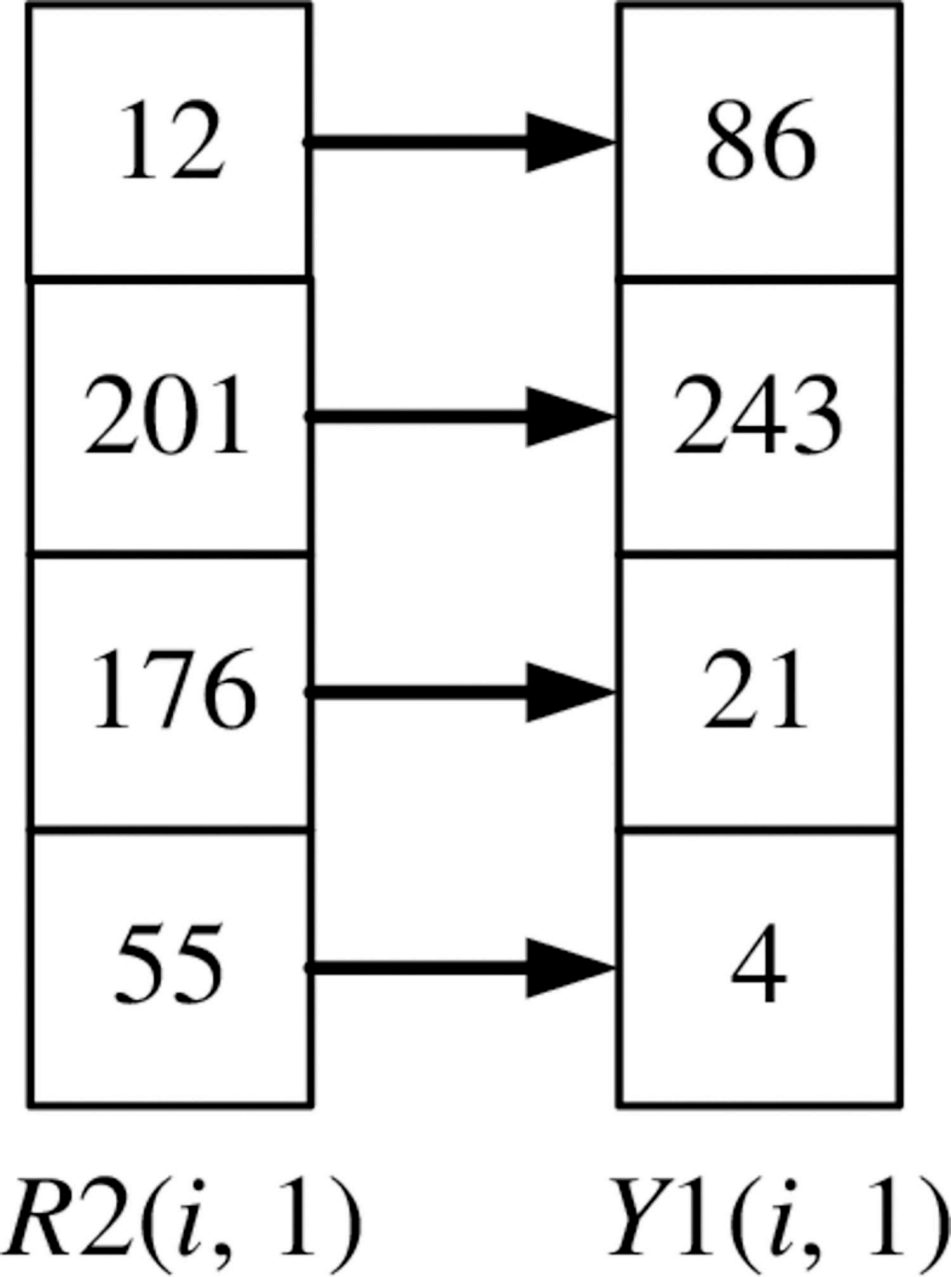
The corresponding relationship between R2(i, 1) and Y1(i, 1).

**Fig 27:**
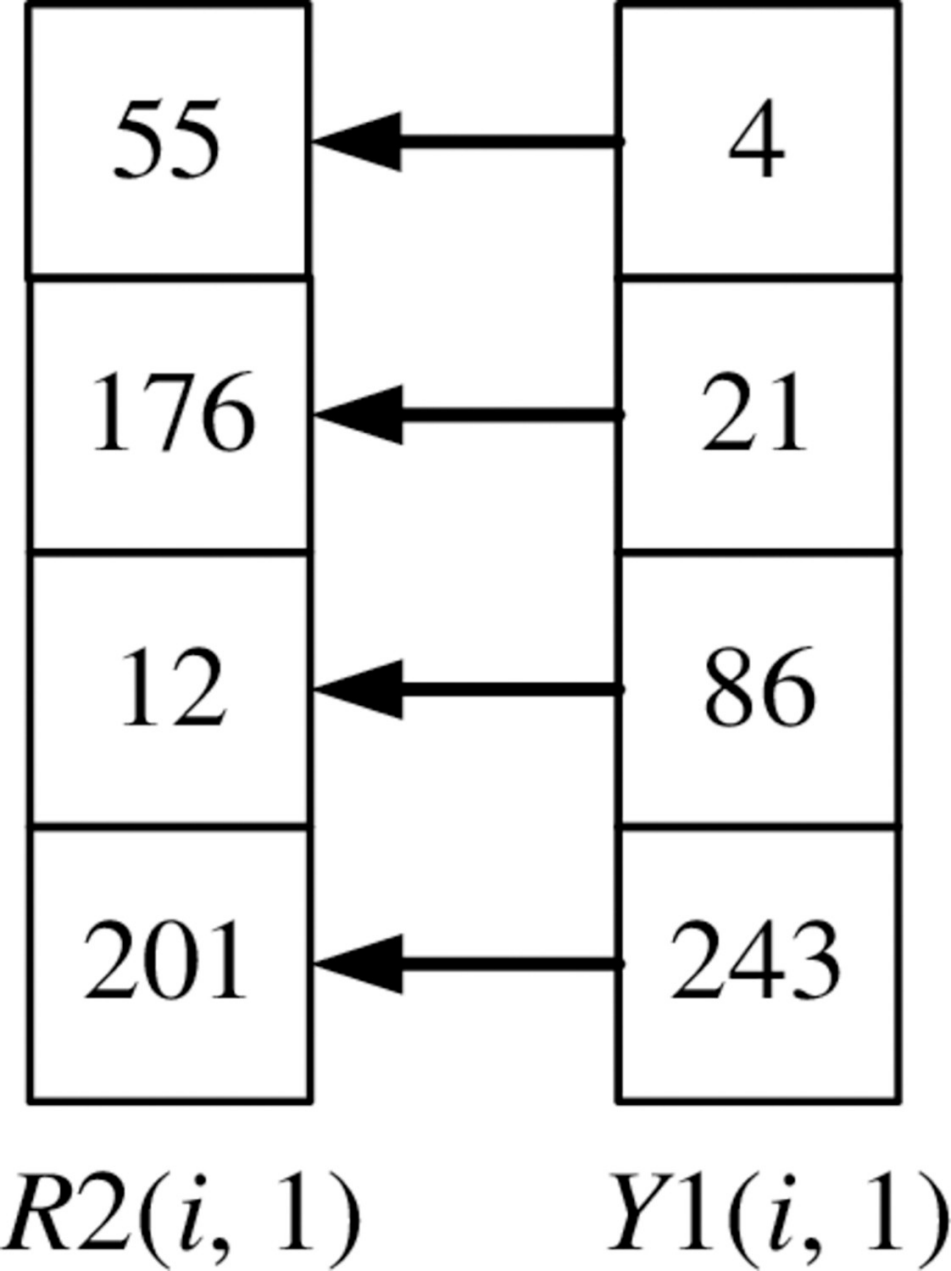
R2(i, 1) scrambles the pixels according to Y1(i, 1).

The scrambling method of *R*2(*i*,1) is shown in Figs 26 and 27. Record the corresponding relationship between each pixel point at the same position of *R*2(*i*,1) and *Y*1(*i*,1), as shown in Fig 26. Arrange *Y*1(*i*,1) and its first three pixels in ascending order using the selection sort algorithm. Disorganize the pixels of *R*2(*i*,1) according to the position of the corresponding pixel of *Y*1(*i*,1), as shown in Fig 27.

If it is not the first row or column element of the image, *R*1(*i*, *j*) is diffused into *R*2(*i*, *j*) through Eq (25). *i* = 2, 3,…, *m*, *j* = 2, 3,…, *n*.


$$\begin{array}{l} R2(i,j)=(R1(i,j)+R2(i-1,j)+R2(1,j-1)+R2(i-1,j-1)\\ +X1(i,j)+k_{28})\mathrm{mod}\;m \end{array}$$ (25)

**Fig 28:**
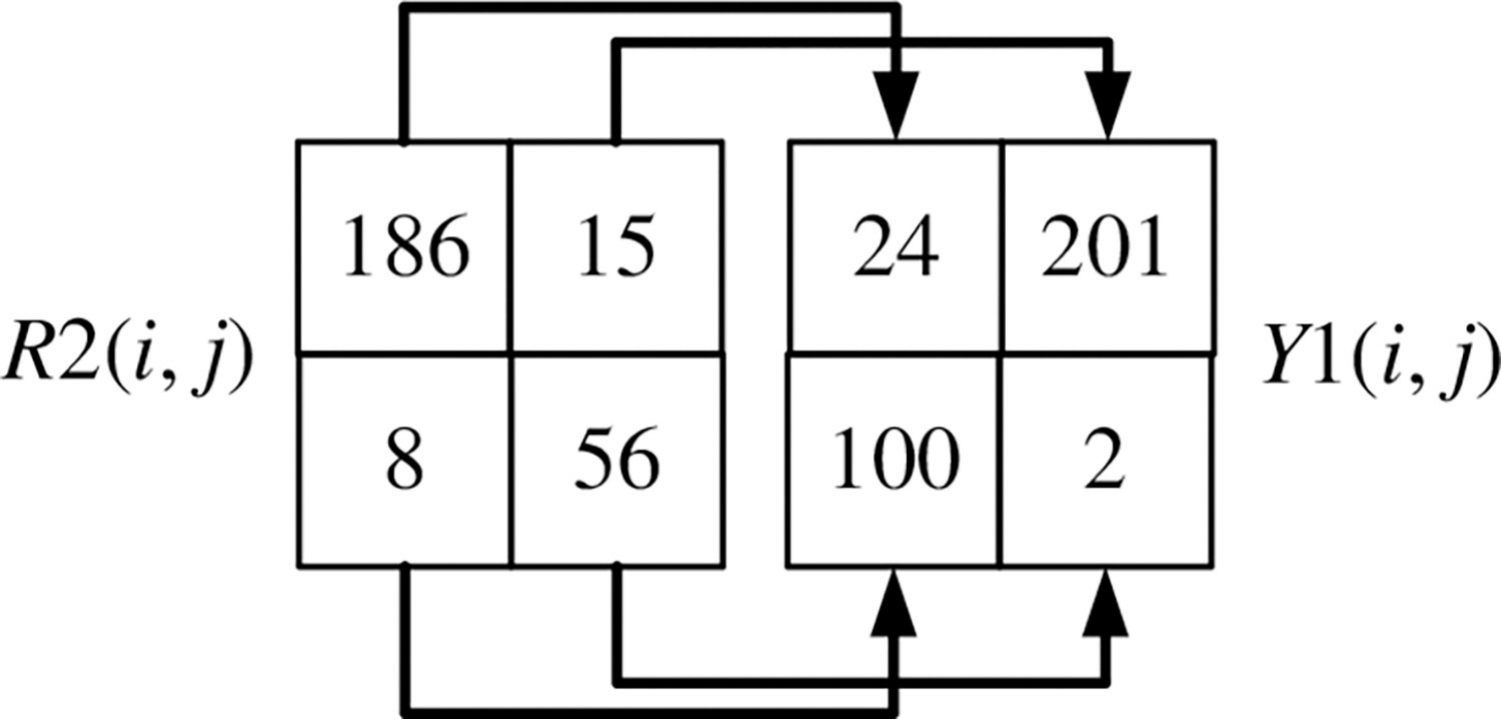
Correspondence between the four points of R2 and the four points of Y1.

**Fig 29:**
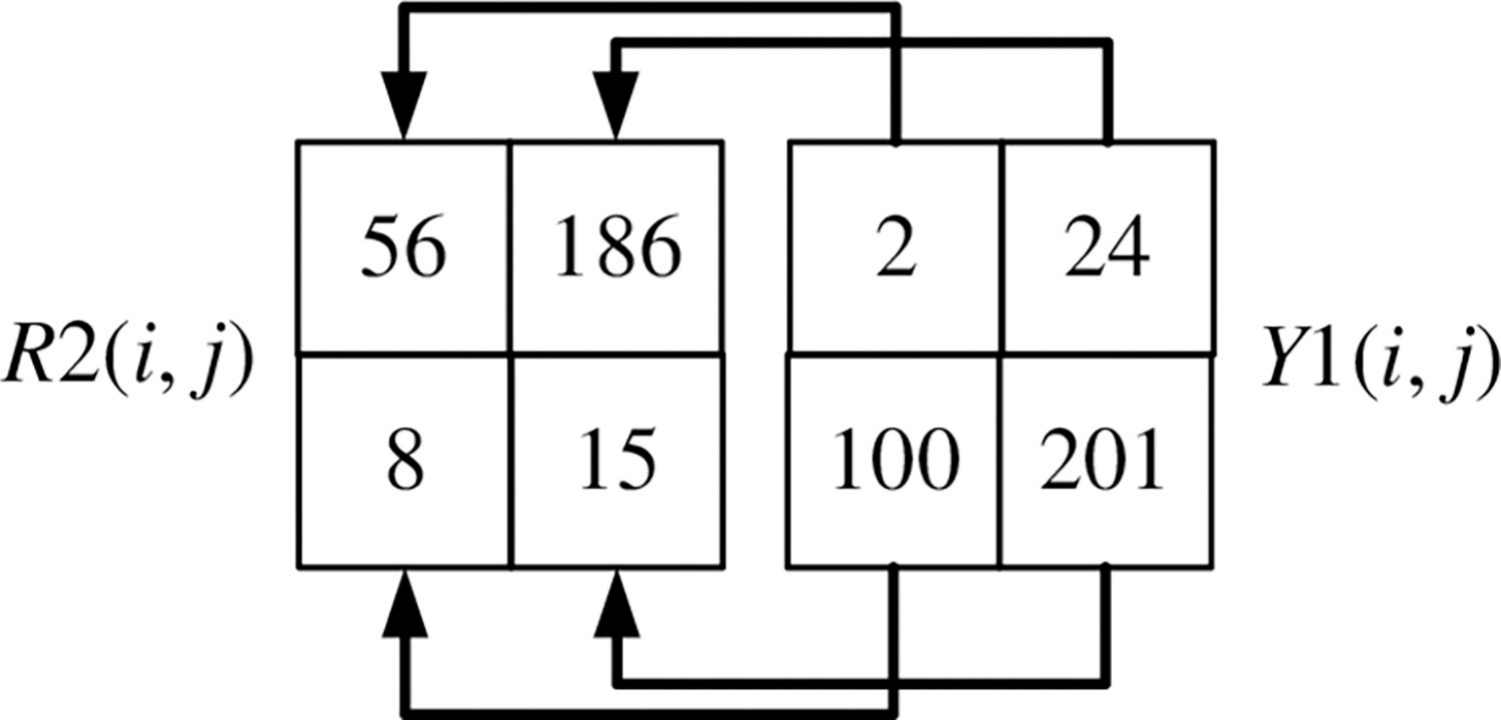
The four points of R2 scramble the pixels according to the four points of Y1.

The scrambling method of *R*2(*i*, *j*) is shown in Figs 28 and 29. Record the corresponding relationship between each pixel point at the same position of *R*2(*i*, *j*), *R*2(*i*-1, *j*), *R*2(*i*, *j*-1), *R*2(*i*-1, *j*-1) and *Y*1(*i*, *j*), *Y*1(*i*-1, *j*), *Y*1(*i*, *j*-1), *Y*1(*i*-1, *j*-1) as shown in Fig 28. Arrange the four pixels of *Y*1(*i*, *j*), *Y*1(*i*-1, *j*), *Y*1(*i*, *j*-1), *Y*1(*i*-1, *j*-1) in ascending order using the selection sort algorithm. After sorting *R*2(*i*, *j*), *R*2(*i*-1, *j*), *R*2(*i*, *j*-1), *R*2(*i*-1, *j*-1) in ascending order of *Y*1(*i*, *j*), *Y*1(*i*-1, *j*), *Y*1(*i*, *j*-1), *Y*1(*i*-1, *j*-1) the position of the corresponding pixel is scrambled, as shown in Fig 29.

(8) (1)—(7) are the first round of encryption, the process of *R*1 turning into *R*2. The encryption process of *G*1 and *B*1 is similar to the encryption process of *R*1. Diffusion calculation of *G*1 and *X*2, scrambling calculation of *G*1 and *Y*2, diffusion calculation of *B*1 and *X*3, scrambling calculation of *B*1 and *Y*3, and *G*2, *B*2 is obtained.

(9) The second round of encryption starts from the lower right corner of *R*2. *R*2(*m*, *n*) is diffused into *R*3(*m*, *n*) through Eq (26).


$$R3(m,n)=(R2(m,n)+X4(m,n)+k_{29})\mathrm{mod}\;m$$ (26)

(10) If it is the last row element of the image, *R*2(*m*, *j*) will be diffused into *R*3(*m*, *j*) through Eq (27). *j* = 2, 3,…, *n*.


$$R3(m,j)=(R2(m,j)+R3(m,j+1)+X4(1,j)+k_{30})\mathrm{mod}\;m$$ (27)

(11) The scrambling method of *R*3(*m*, *j*) is the same as (3). *R*3(*m*, *j*) and *Y*4(*m*, *j*) are calculated. The specific method is the same as the calculation method of *R*2(1, *j*) and *Y*1(1, *j*) in Fig 7.

(12) If it is the last column element of the image, *R*2(*i*, *n*) is diffused into *R*3(*i*, *n*) through Eq (28). *i* = 2, 3,…, *m*.


$$R3(i,n)=(R2(i,n)+R3(i+1,n)+X4(i,n)+k_{31})\mathrm{mod}\;n$$ (28)

(13) The scrambling method of *R*3(*i*, *n*) is the same as (5). *R*3(*i*, *n*) and *Y*4(*i*, *n*) are calculated. The specific method is the same as the calculation method of *R*2(*i*, 1) and *Y*1(*i*, 1) in Fig 8.

(14) If it is not the last row or column element of the image, *R*2(*i*, *j*) is diffused into *R*3(*i*, *j*) through Eq (29). *i* = 2, 3,…, *m*, *j* = 2, 3,…, *n*.


$$\begin{array}{l} R3(i,j)=(R2(i,j)+R3(i+1,j)+R3(i,j+1)+R3(i+1,j+1)\\ +X4(1,j)+k_{32})\mathrm{mod}\;m \end{array}$$ (29)

(15) The scrambling method of *R*3(*i*, *j*) is the same as (7). Calculate *R*3(*i*, *j*), *R*3(*i*+1, *j*), *R*3(*i*, *j*+1), *R*3(*i*+1, *j*+1) and *R*3(*i*, *j*), *R*3(*i*+1, *j*), *R*3(*i*, *j*+1), *R*3(*i*+1, *j*+1) the specific method is the same as the calculation method of each element in Fig 9.

(16) (9)—(15) is the second round of encryption, the process of *R*2 turning into *R*3. The encryption process of *G*2 and *B*2 is similar to the encryption process of *R*2. Diffusion calculation of *G*2 and *X*5, scrambling calculation of *G*2 and *Y*5, diffusion calculation of *B*2 and *X*6, scrambling calculation of *B*2 and *Y*6, and *G*3, *B*3 is obtained.

(17) Combine *R*3, *G*3, *B*3 into cipher *C*, and the encryption algorithm ends.

### 3.4 Image decryption algorithm

**Fig 30:**
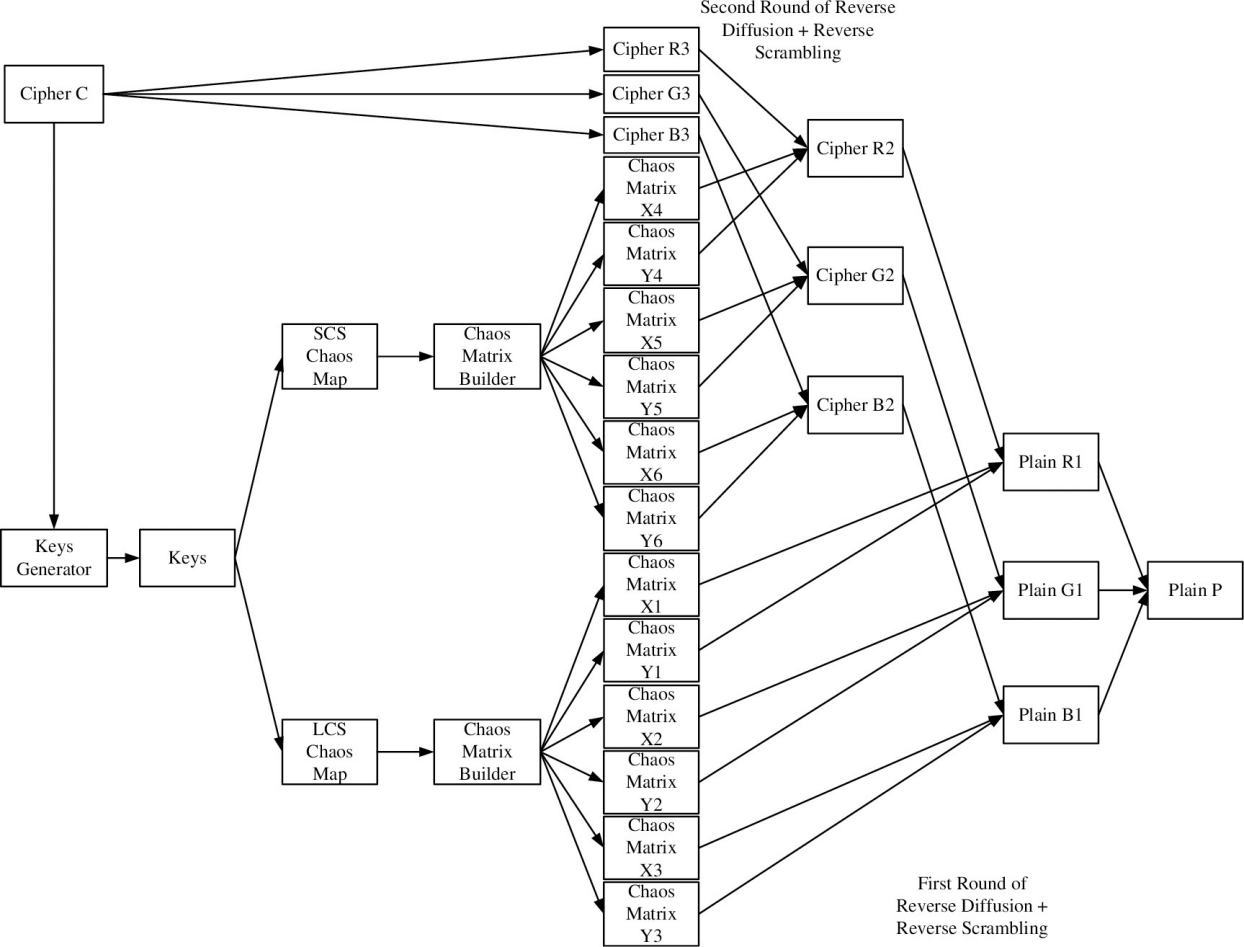
Image decryption algorithm flow chart.

The flow chart of this image decryption algorithm is shown in Fig 30. Reverse the entire encryption process. Enter the cipher and key to get the plain. The specific process will not be explained in detail.

## 4 Experimental results and analysis

### 4.1 Experimental environment and results

#### 4.1.1 Experimental environment

(1) Hardware environment: Intel Core i9-12900H 14-core processor, main frequency is 2.50GHz. The memory is DDR5 4800MHz 32GB. 1TB SSD.

(2) Software environment: Windows 11 Home Edition, Matlab R2018b.

#### 4.1.2 Experimental results

The experimental images in this article use the USC-SIPI database. The URL of the database is http://sipi.usc.edu/database/. The experiment uses color images of 256×256 size. The encryption and decryption results of the Pepper image are shown in Figs 31–42.

**Fig 31:**
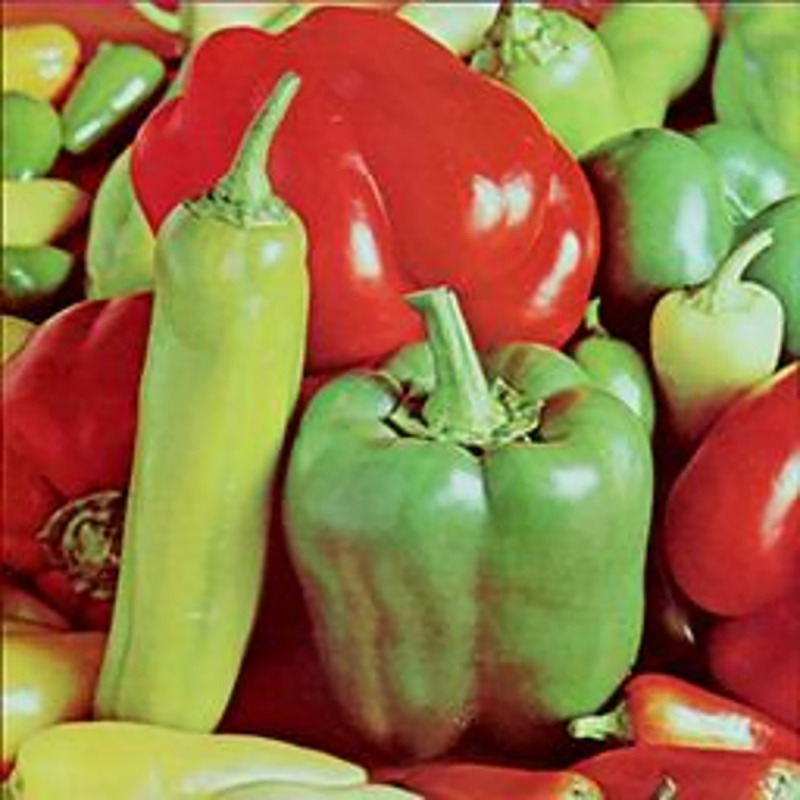
Color plain.

**Fig 32:**
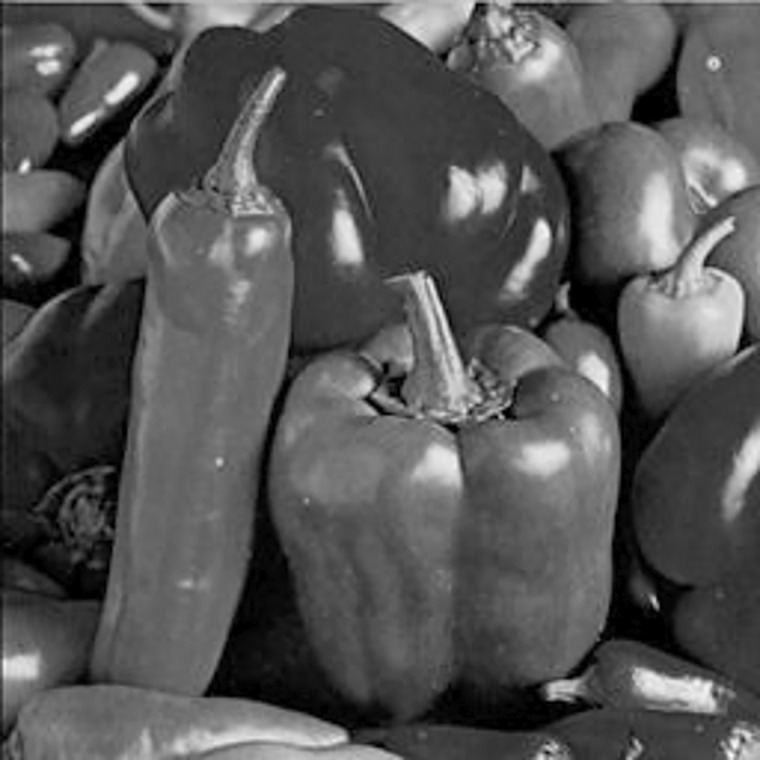
Red plain.

**Fig 33:**
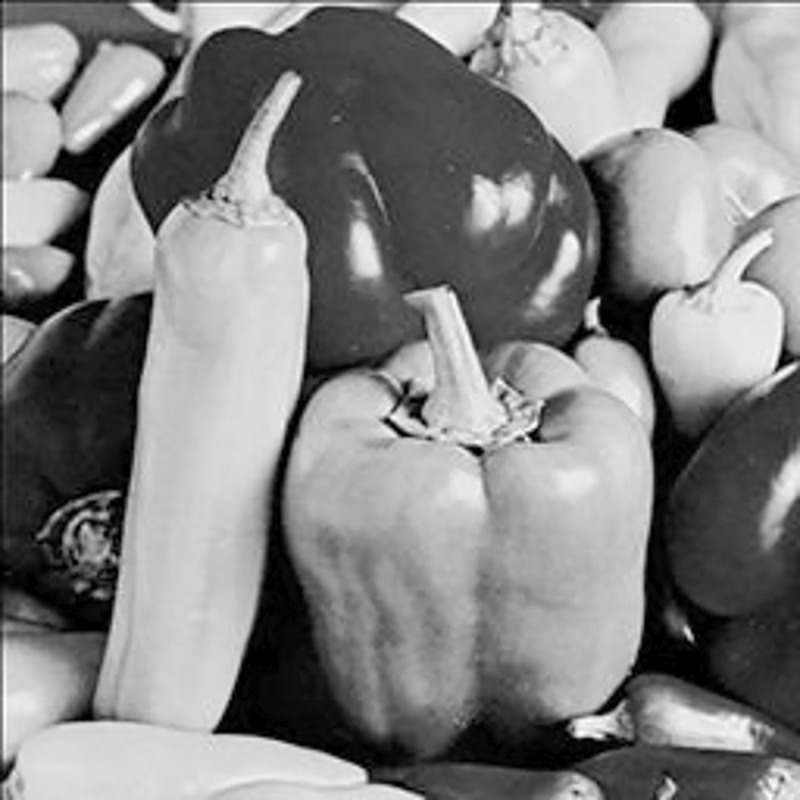
Green plain.

**Fig 34:**
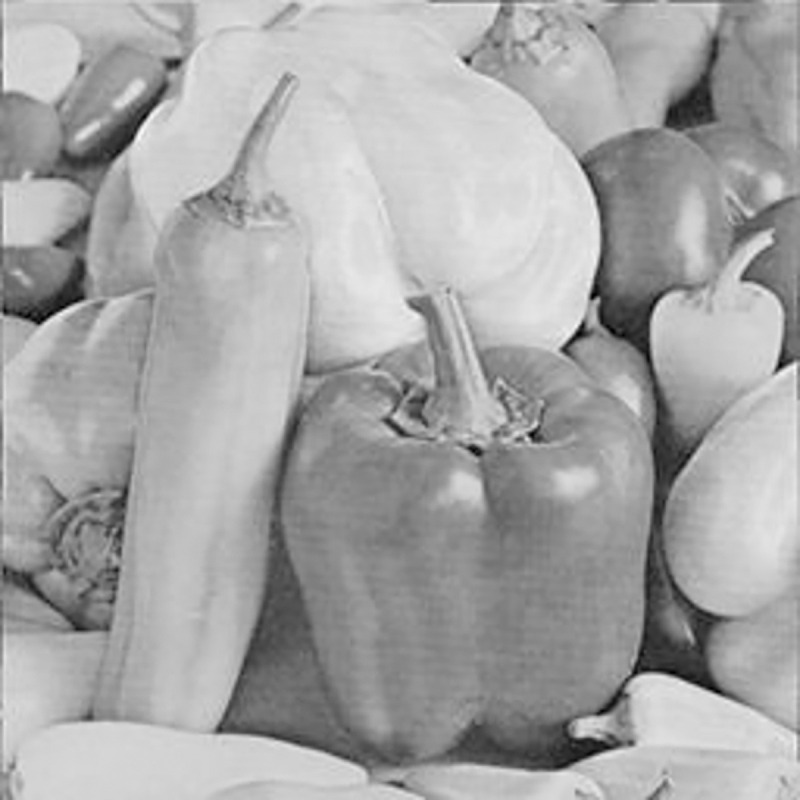
Blue plain.

**Fig 35:**
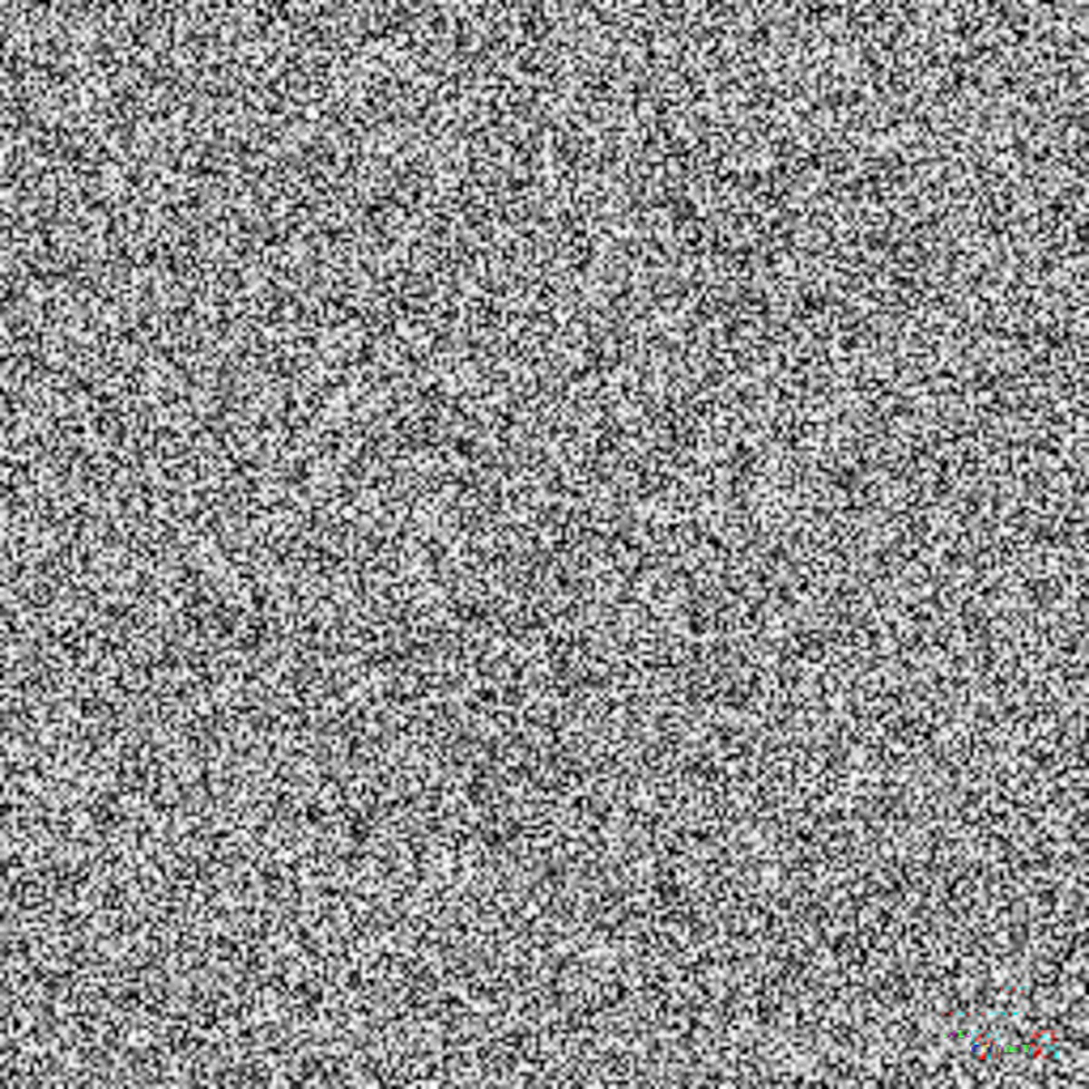
Encrypted red plain.

**Fig 36:**
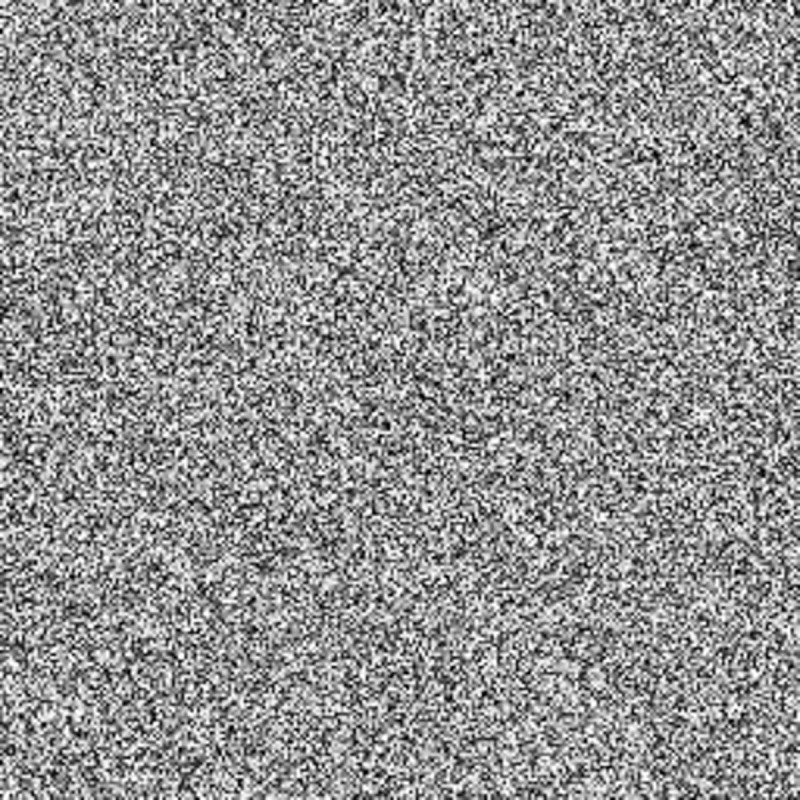
Encrypted green plain.

**Fig 37:**
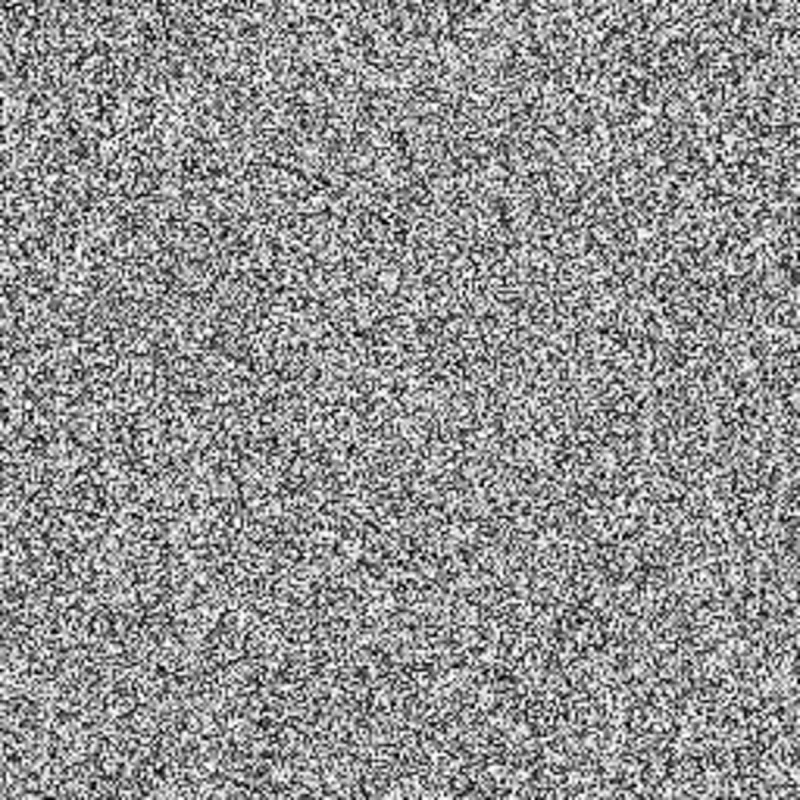
Encrypted blue plain.

**Fig 38:**
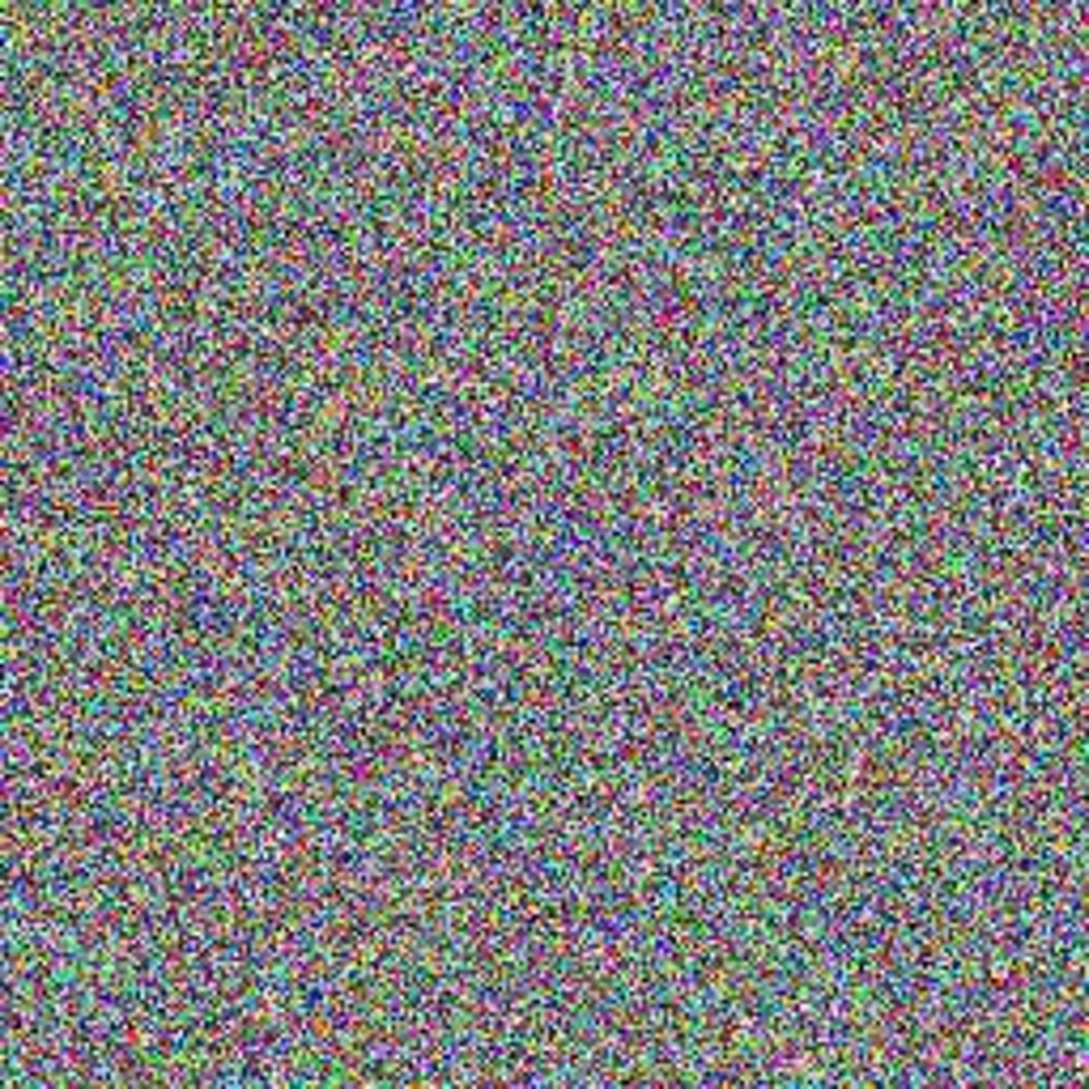
Encrypted color plain.

**Fig 39:**
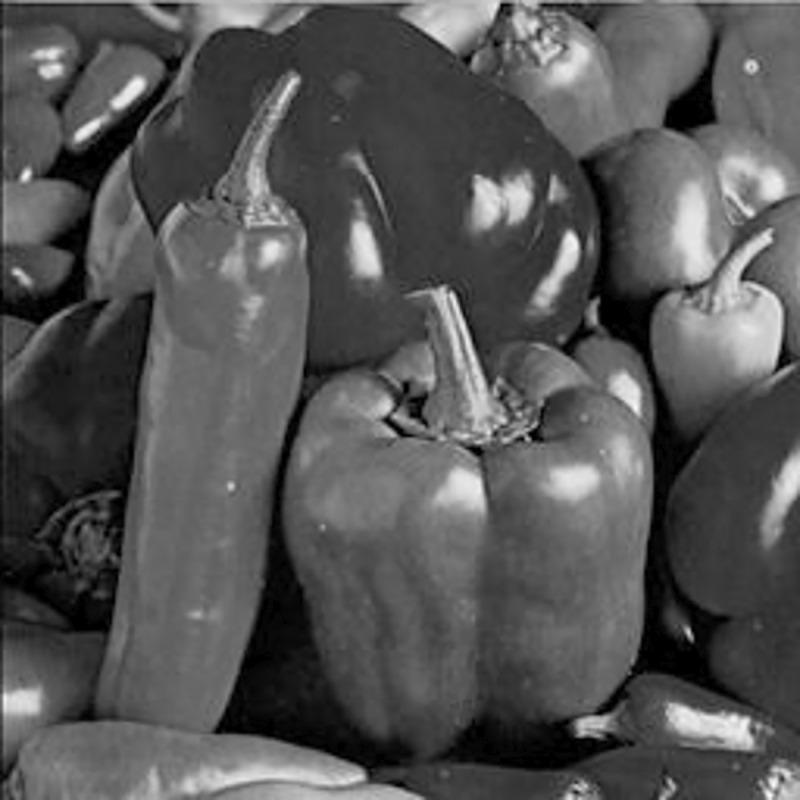
Decrypted red plain.

**Fig 40:**
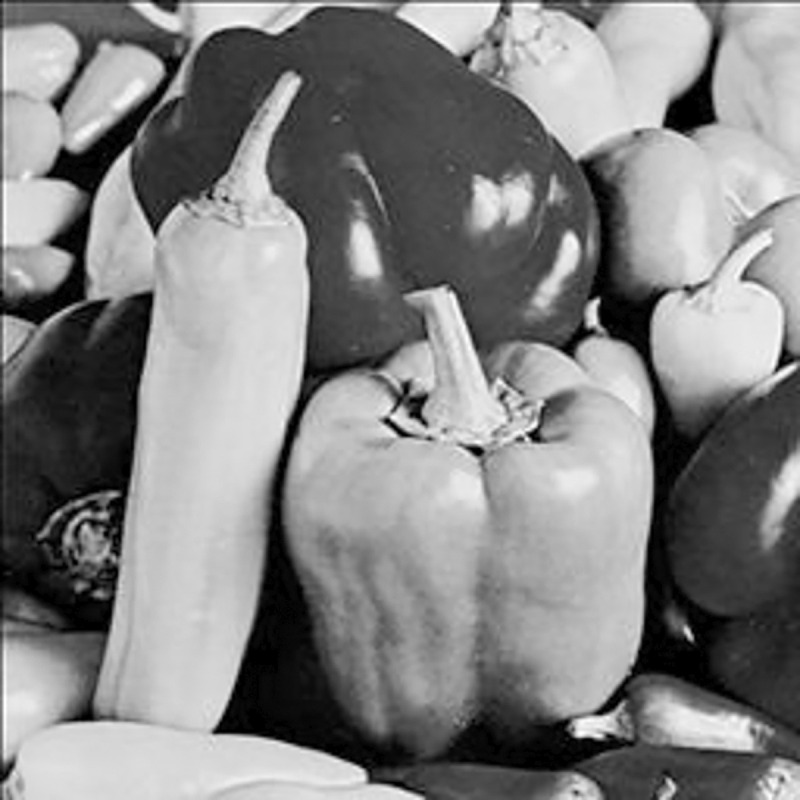
Decrypted green plain.

**Fig 41:**
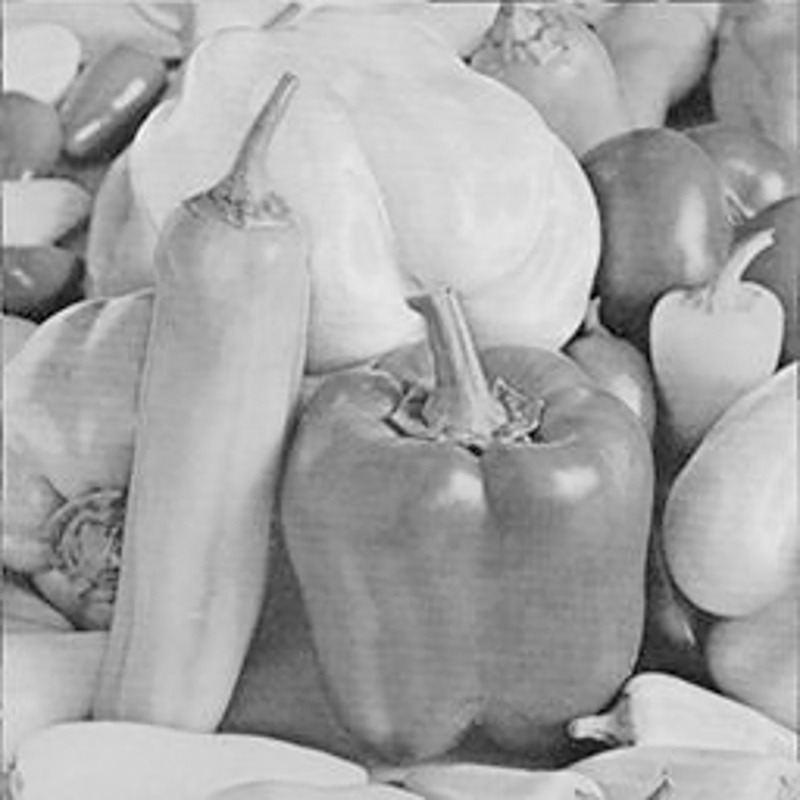
Decrypted blue plain.

**Fig 42:**
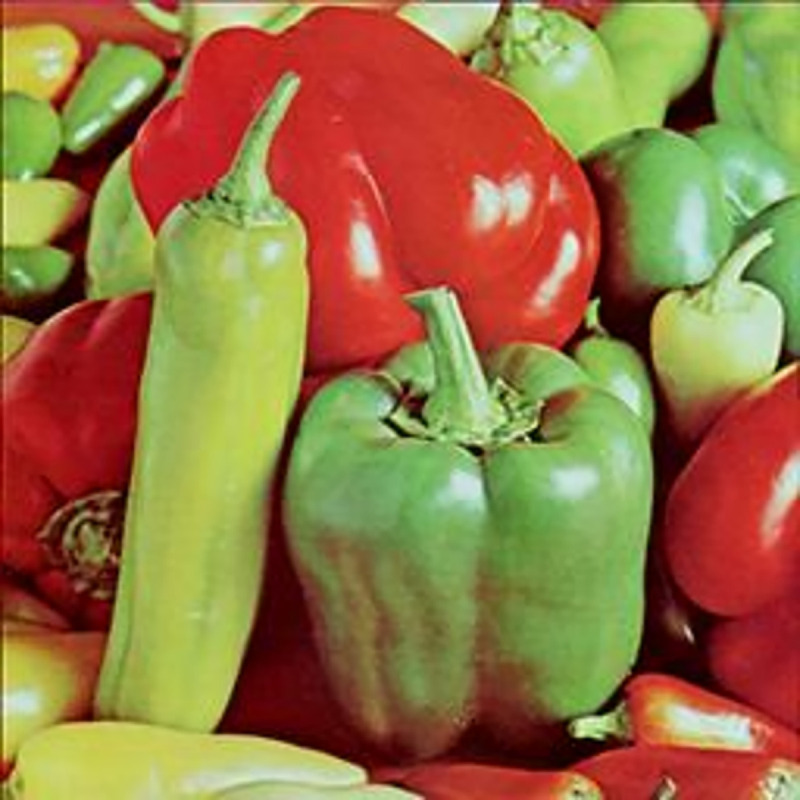
Decrypted color plain.

Fig 31 is colored plain text. Figs 32–34 are the plain decomposed into three channels of red, green and blue, and Figs 35–37 are the cipher after three-channel encryption. Fig 38 is the color cipher merged with the three-channel cipher. Figs 39–41 are the plain after three-channel decryption. Fig 42 is the colored plain merged with plain after three-channel decryption. The following is a comparison of various indicators between the algorithm in this article and other fast image encryption algorithms.

### 4.2 Experimental performance analysis

#### 4.2.1 Encryption and decryption speed

The speed of image encryption and decryption based on chaos mapping depends on multiple factors, including the selected encryption algorithm, computer hardware performance, image size and complexity, etc. Here we introduce the experiments under the above-mentioned software and hardware configuration. After experimenting with 20 pictures, the average experimental results are compared with the encryption and decryption speeds of references, as shown in Table 2.

**Table 2:** Encryption and decryption time (unit: Seconds).

Algorithm	Proposed	Ref. [13]	Ref. [14]	Ref. [15]	Ref. [16]	Ref. [17]
**Encryption time**	**0.81**	4.5	0.82	0.75	0.83	1.21
**Decryption time**	**0.80**	4.3	0.81	0.74	0.81	1.12

As can be seen from Table 2, the encryption and decryption time of this algorithm is shorter. In the era of big data, image data sets are getting larger and larger. Algorithms with short encryption and decryption times have more application prospects.

About the time complexity of this algorithm. Assume that the size of the plain image is *m*×*n*. The chaotic system iteratively generates 12 chaotic sequences with a length of *mn*. The computational complexity is 12*mn*. Two rounds of diffusion + scrambling encryption process, each round generates 3 ciphers, and the computational complexity is 2×2×3×*mn* = 12*mn*. Overall, the time complexity of the algorithm in this chapter is O(24*mn*). This time complexity is acceptable.

#### 4.2.2 Keys space

In this encryption algorithm, the keys involved are a1, *a*_1_, *a*_2_, *β*_1_, *β*_2_, *θ*_1_, *θ*_2_, *i*_1_, *i*_2_, *i*_3_, *j*_1_, *j*_2_, *j*_3_, a total of 12 keys. The calculation of each key uses 2 value of *k* in Eq (15). Each *k* is 8 bits. Therefore, the key space is ((2^8^)^2^)^12^ = 2^192^>2^100^, which is enough to resist exhaustive attacks. The comparison between the key space of this paper experiments and other literature is shown in Table 3. The key space of the algorithm in this chapter is not the largest. However, on the basis of resisting exhaustive attacks, a smaller key space can increase the encryption speed.

**Table 3:** Keys space.

Algorithm	Proposed	Ref. [13]	Ref. [14]	Ref. [15]	Ref. [16]	Ref. [17]
**Keys space**	2^691^	2^360^	2^281^	**2** ^**827**^	2^380^	2^409^

#### 4.2.3 Correlation coefficient

The correlation coefficient of chaos map image encryption is an indicator used to evaluate the performance of encryption algorithms. The correlation coefficient is often used to compare the similarity between plain and cipher to measure the quality of image encryption algorithms. The value of the correlation coefficient can provide a measure of the degree of encryption protection of an image.

The correlation coefficient is usually expressed as a value ranging from -1 to 1 and has the following meaning:

1. If the correlation coefficient is equal to 1, it means that the original image and the encrypted image are exactly the same, there is no information loss, and the encryption is perfect.
2. If the correlation coefficient is close to 1, it means that the original image and the encrypted image are very similar, but there may be some minor changes or noise.
3. If the correlation coefficient is close to 0, it means that there is no obvious similarity between the original image and the encrypted image, and encryption leads to large information changes.
4. If the correlation coefficient is close to -1, it means that the original image and the encrypted image are completely different, and encryption has caused serious information changes.

The correlation coefficient is calculated through Eqs (30)–(33).


$$r_{XY}=\frac{COV(X,Y)}{\sqrt{D(X)D(Y)}}$$ (30)

$$E(X)=\frac{1}{R}\sum_{i=1}^{R}x_{i}$$ (31)

$$D(X)=\frac{1}{R}{\sum_{i=1}^{R}\left(x_{i}-E(X)\right)}^{2}$$ (32)

$$COV(X,Y)=\frac{1}{R}\sum_{i=1}^{R}\left(x_{i}-E(X)(y_{i}-E(Y)\right)$$ (33)

Among them, *R* represents the number of pixels in the picture. *E* (*X*), *E* (*Y*) represent the mathematical expectations of *X*, *Y* respectively. *D* (*X*), *D* (*Y*) represents the variance of *X*, *Y*. *COV* (*X*, *Y*) represents the covariance of *X*, *Y*. *r*_*XY*_ represents the correlation coefficient of *X*, *Y*.

**Fig 43:**
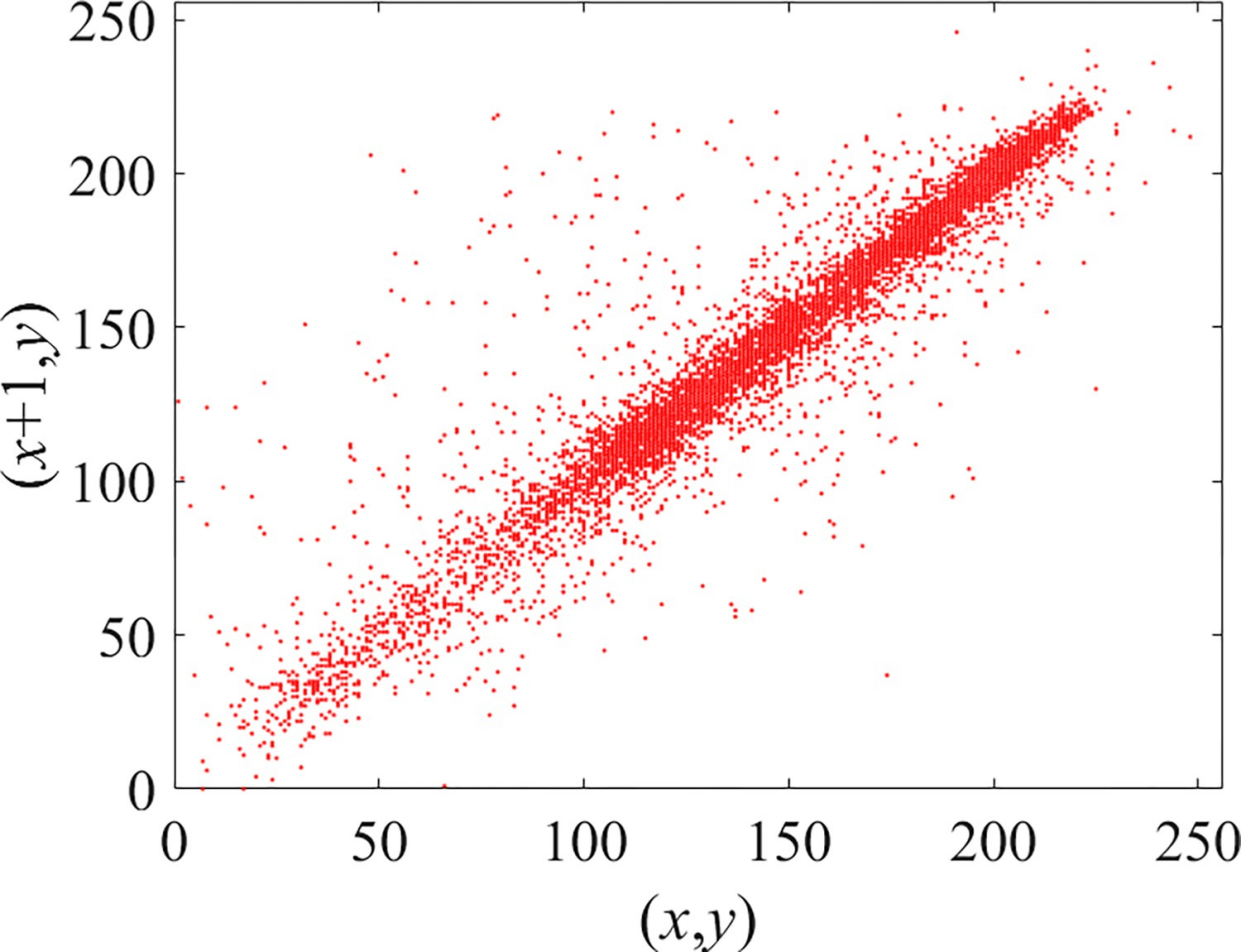
Red plain horizontal correlation.

**Fig 44:**
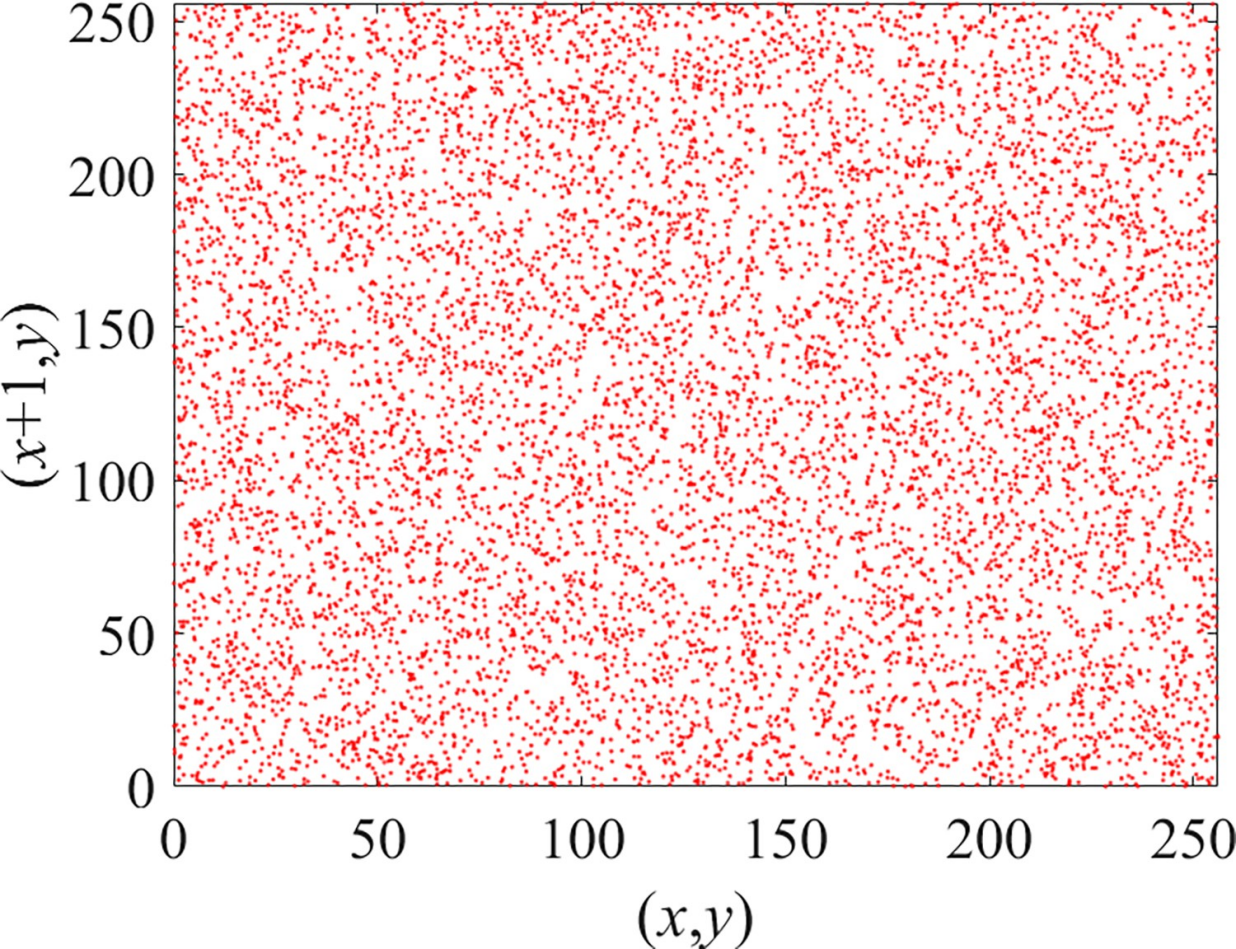
Red cipher horizontal correlation.

**Fig 45:**
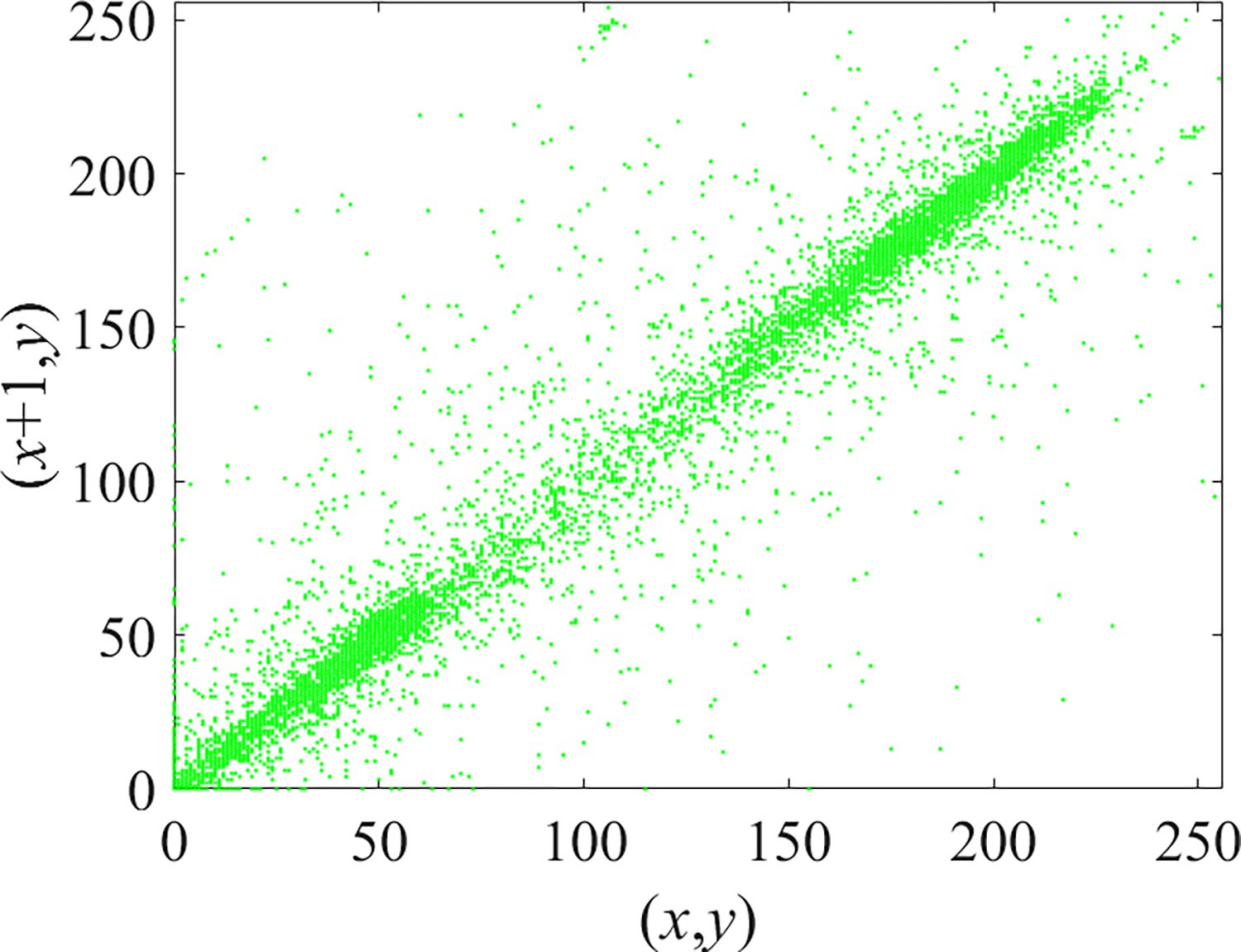
Green plain horizontal correlation.

**Fig 46:**
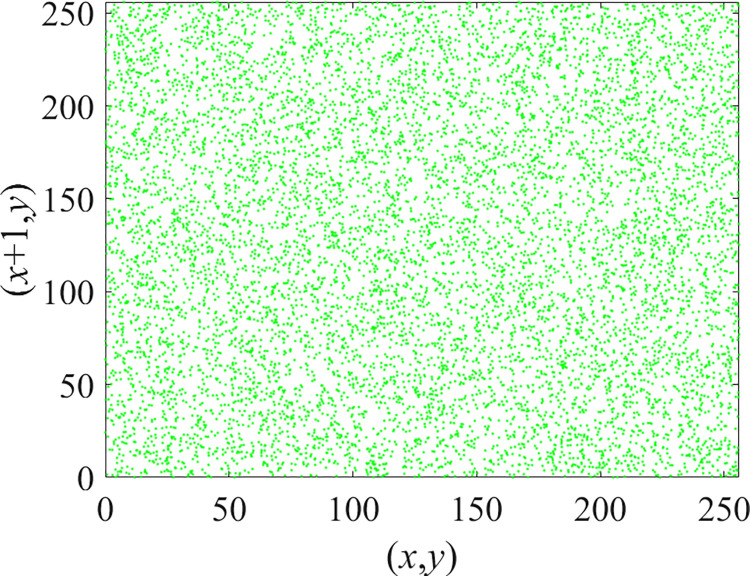
Green cipher horizontal correlation.

**Fig 47:**
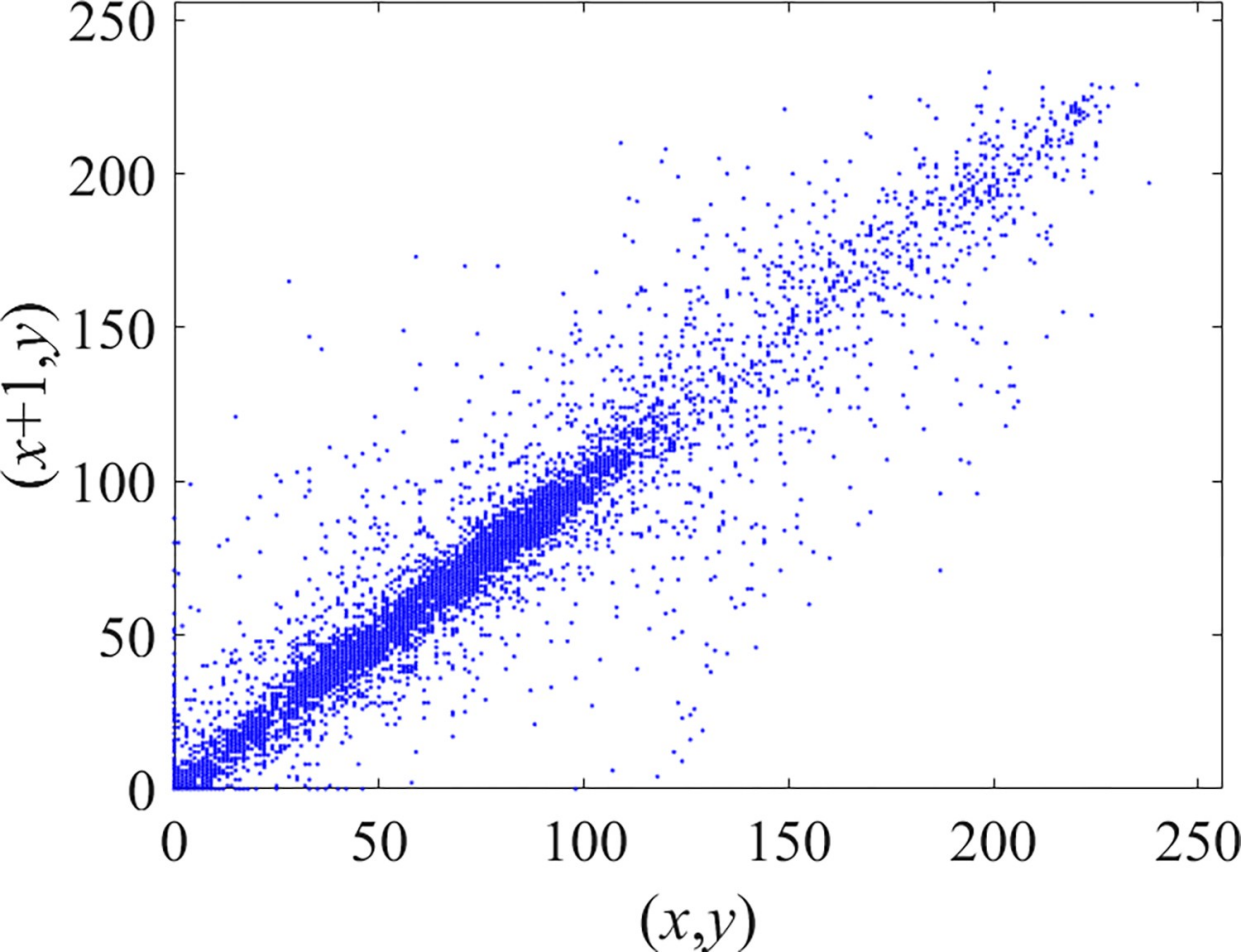
Blue plain horizontal correlation.

**Fig 48:**
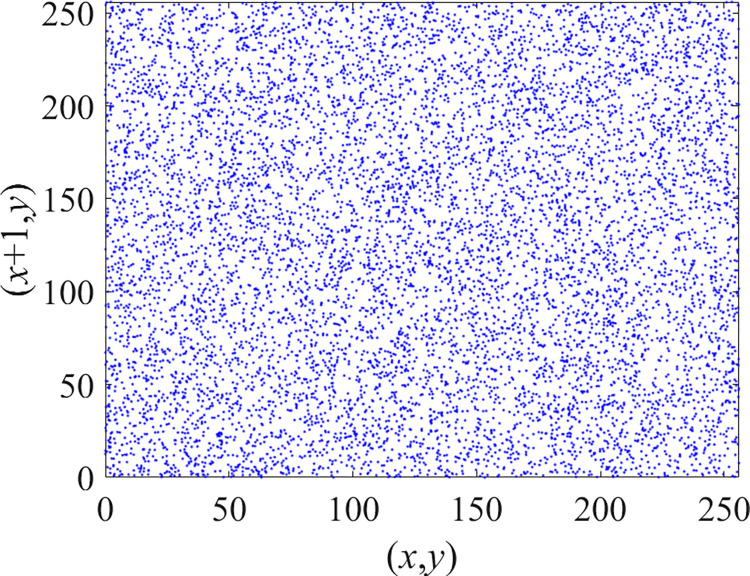
Blue cipher horizontal correlation.

**Fig 49:**
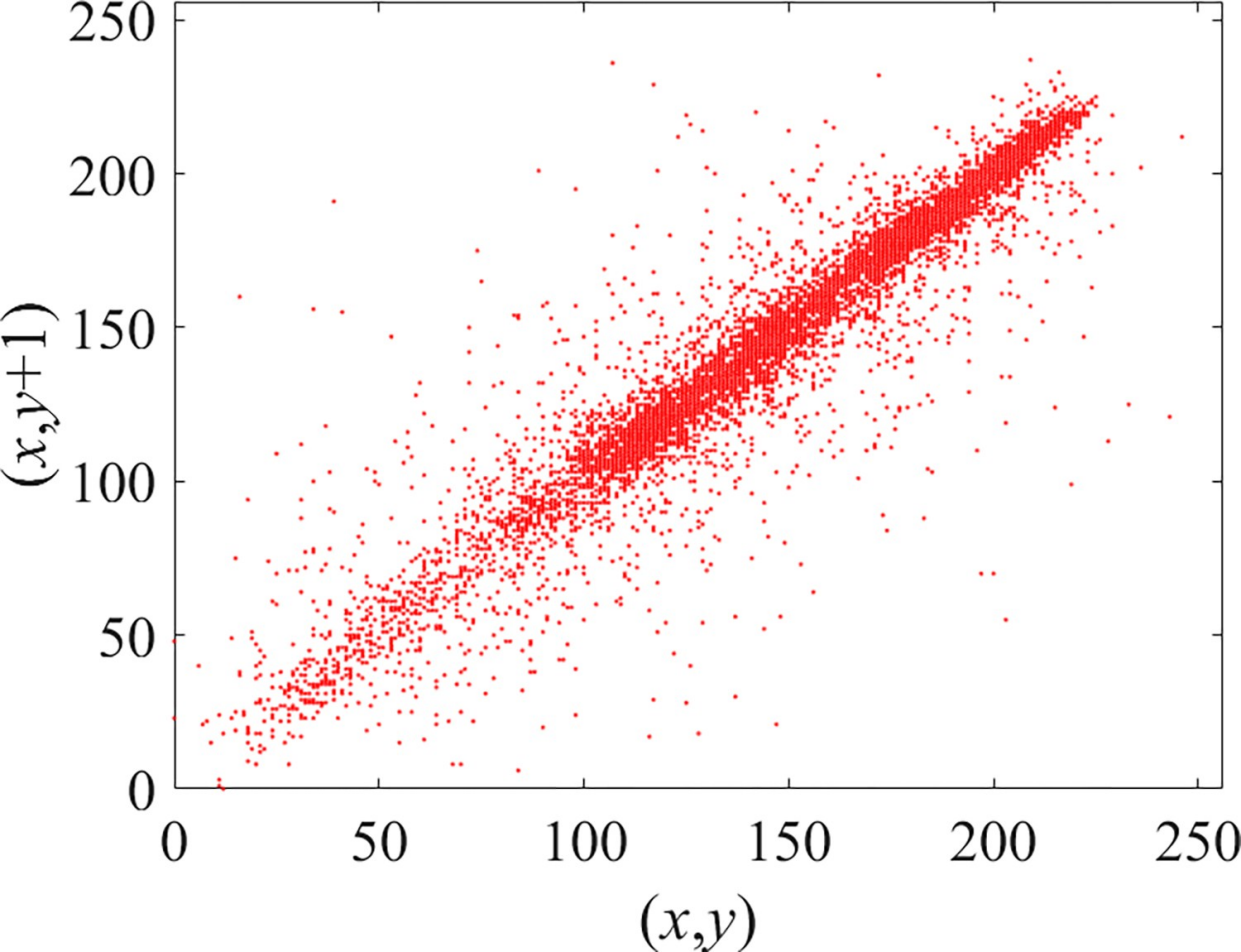
Red plain vertical correlation.

**Fig 50:**
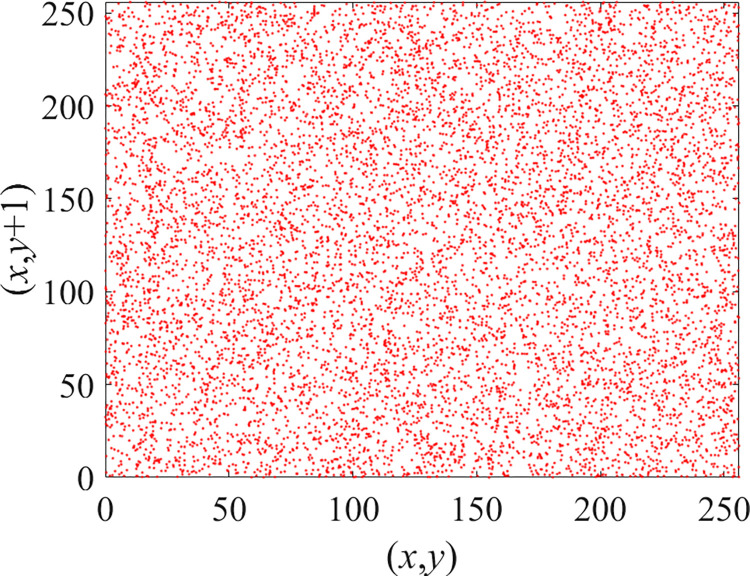
Red cipher vertical correlation.

**Fig 51:**
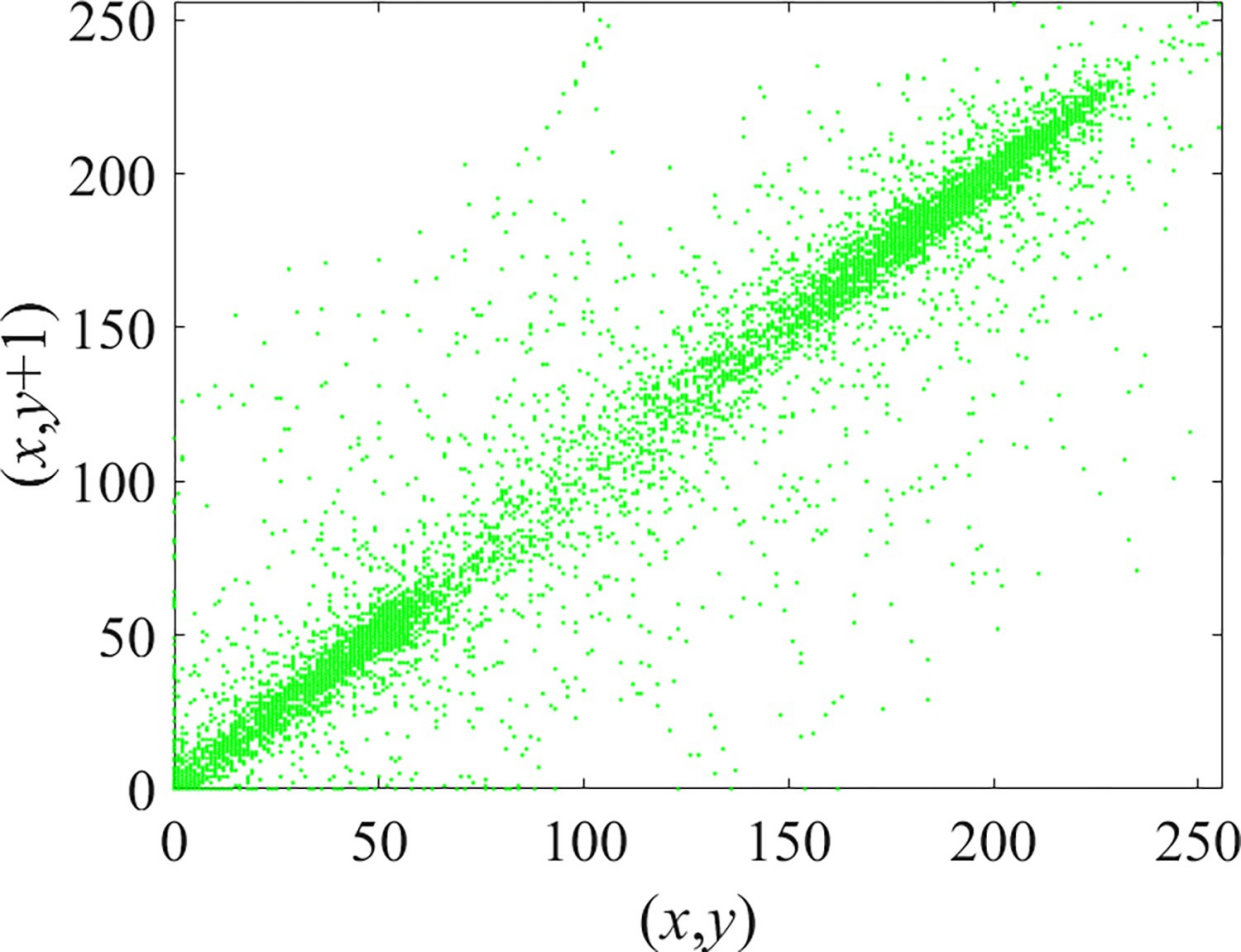
Green plain vertical correlation.

**Fig 52:**
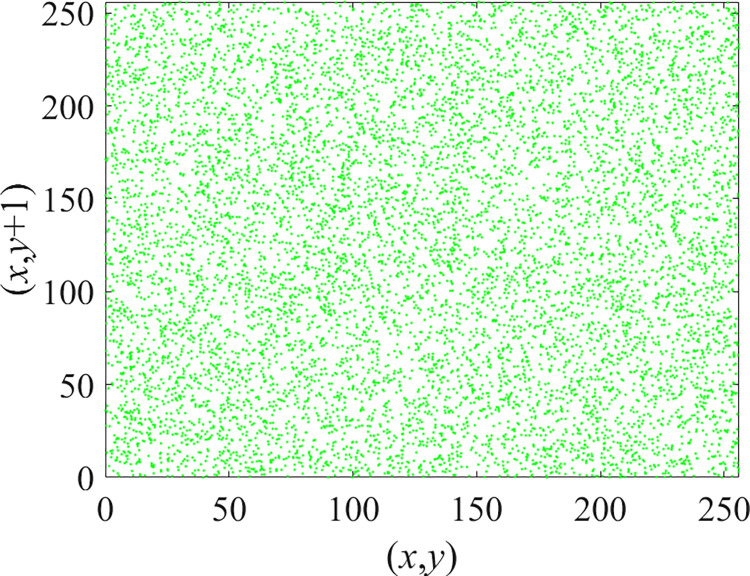
Green cipher vertical correlation.

**Fig 53:**
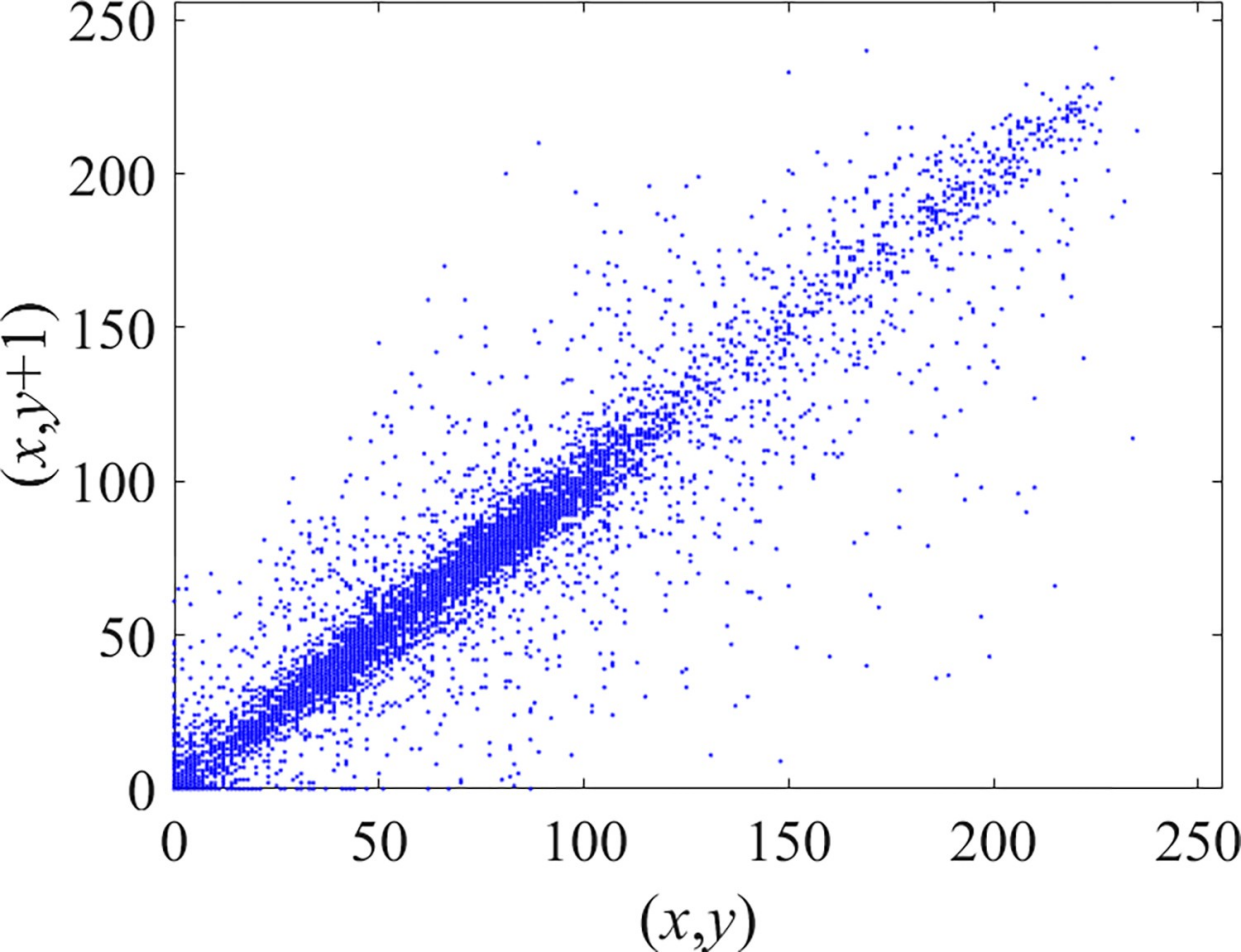
Blue plain vertical correlation.

**Fig 54:**
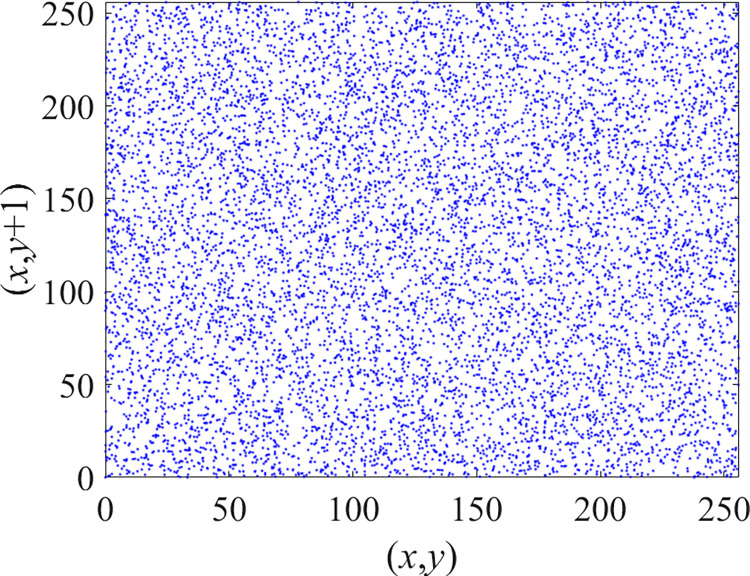
Blue cipher vertical correlation.

**Fig 55:**
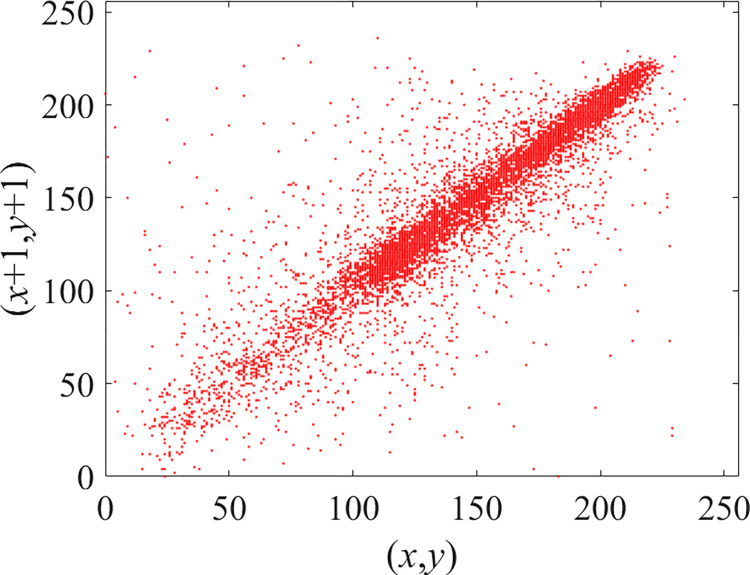
Red plain diagonal correlation.

**Fig 56:**
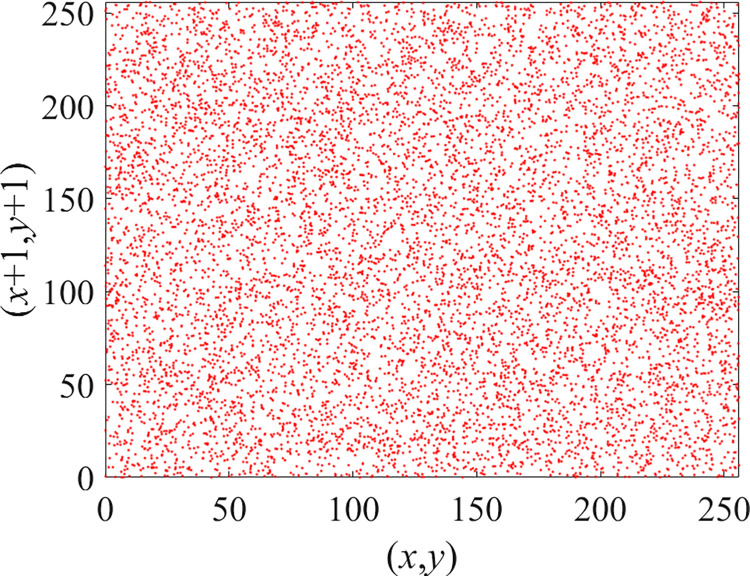
Red cipher diagonal correlation.

**Fig 57:**
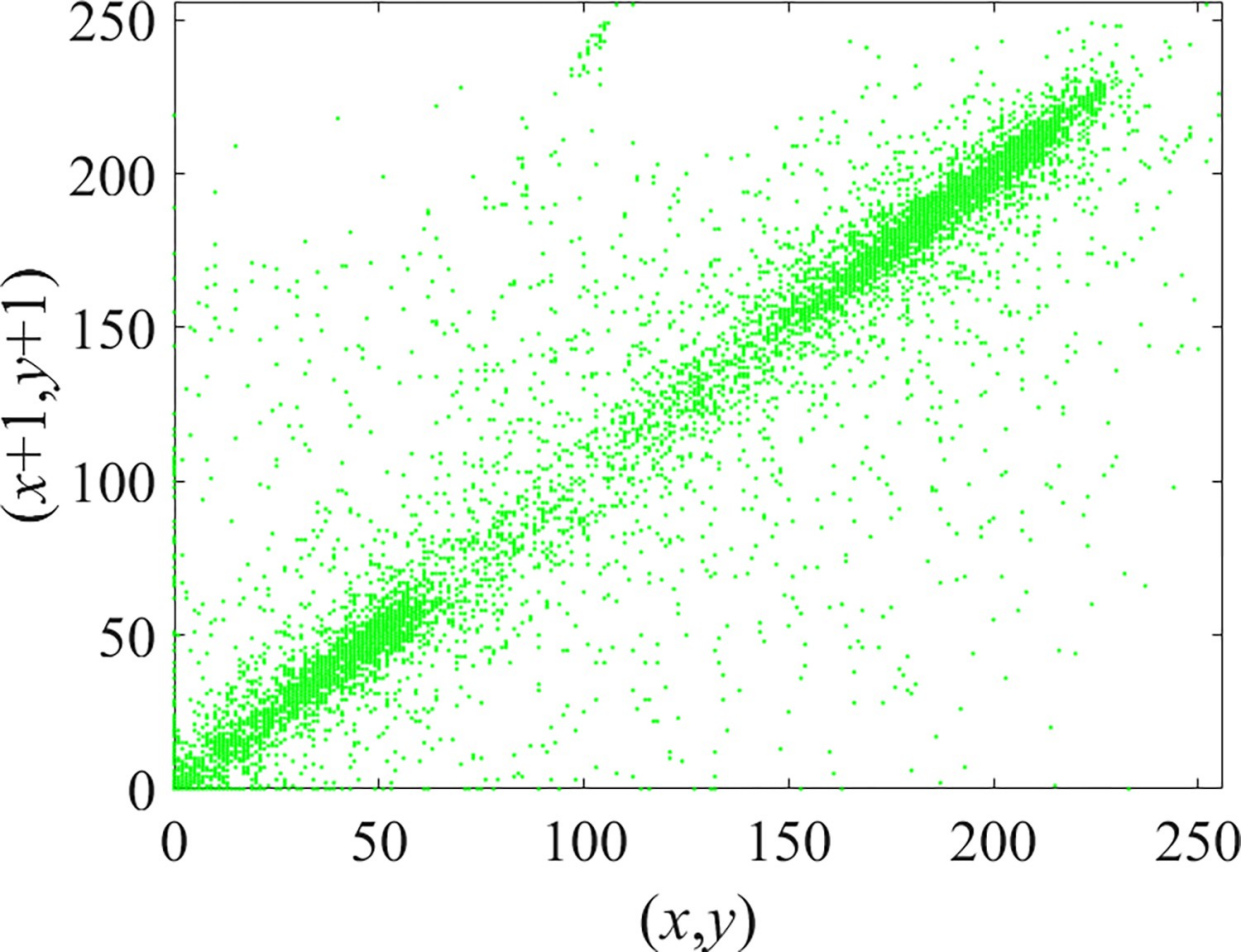
Green plain diagonal correlation.

**Fig 58:**
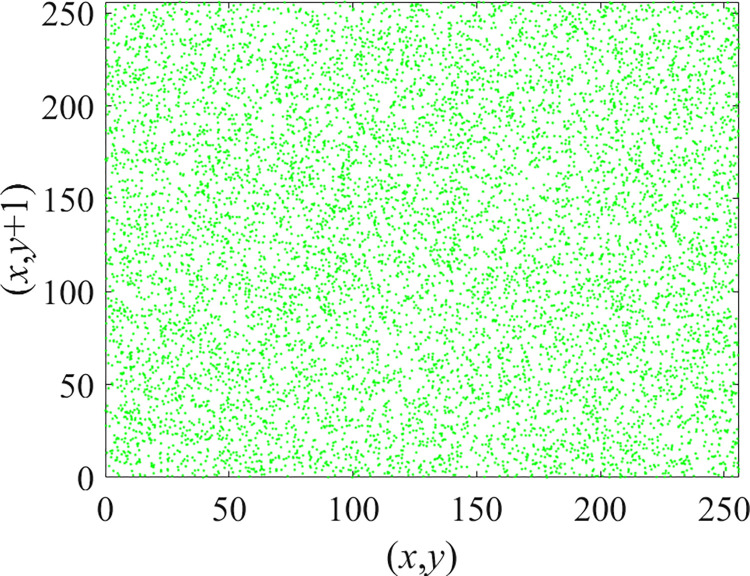
Green cipher diagonal correlation.

**Fig 59:**
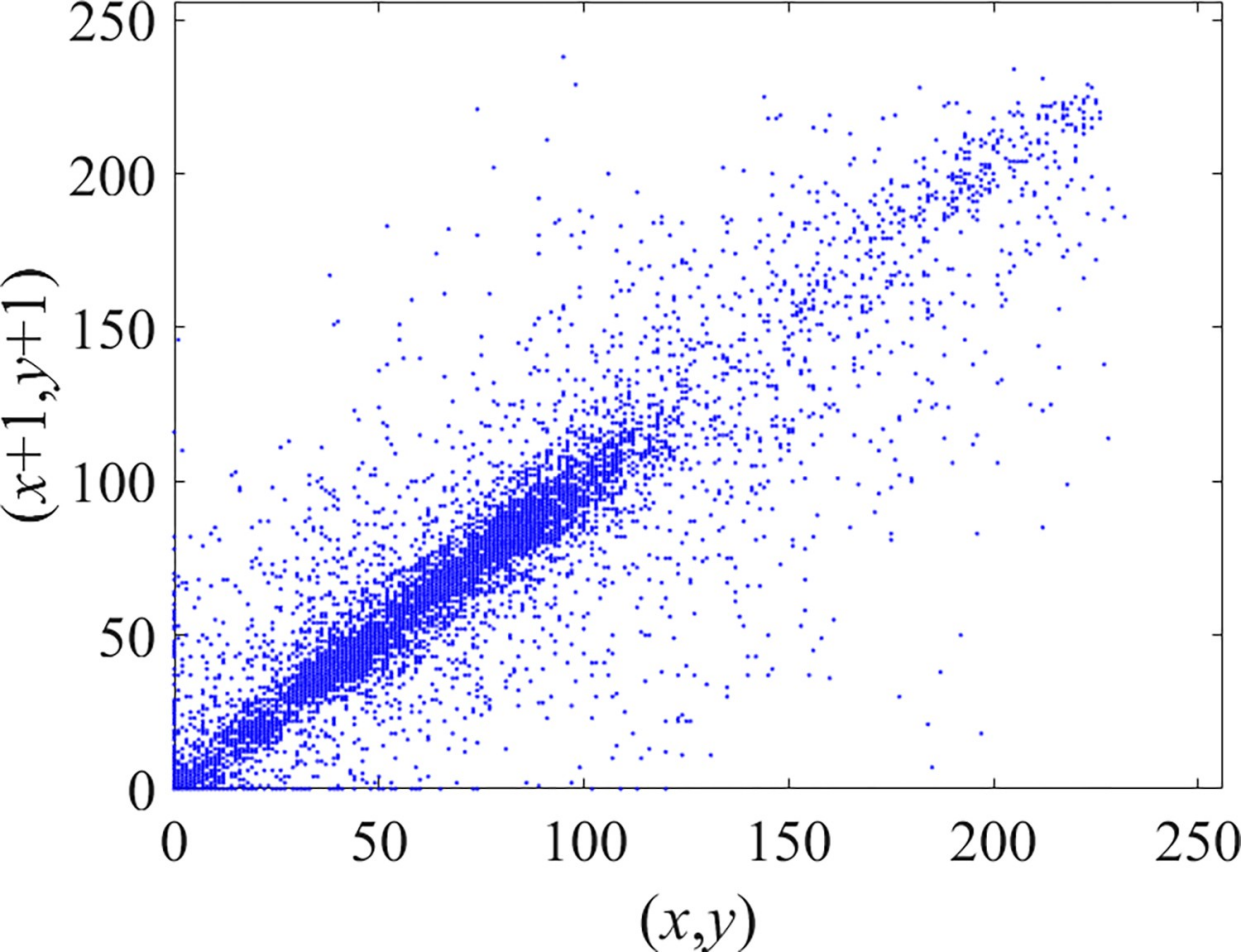
Blue plain diagonal correlation.

**Fig 60:**
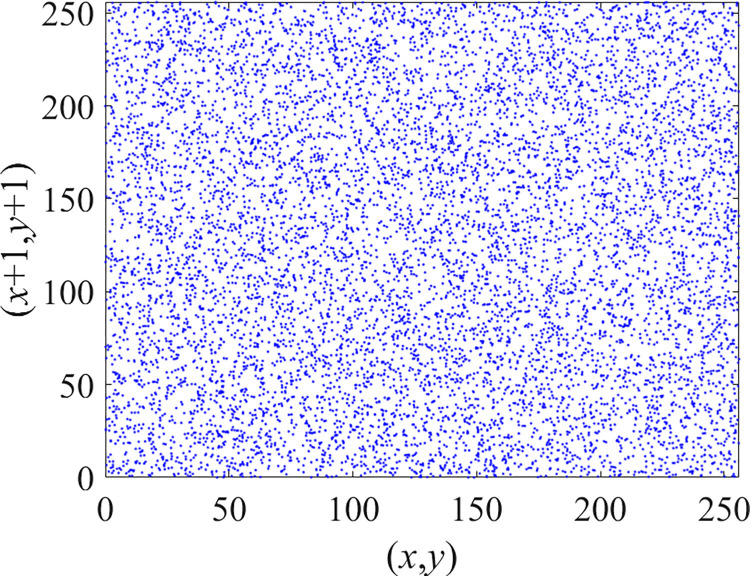
Blue cipher diagonal correlation.

Through the calculation of Formulas (30) to (33), the pepper image is encrypted using the algorithm in this paper. The experimental results of the correlation coefficient of the image are shown in Table 4. Each value is rounded to four decimal places. The correlation coefficient of plain text images is close to 1. The correlation coefficient of the corresponding cipher image is close to 0. It shows that the algorithm in this paper eliminates the correlation between adjacent pixels. The average cipher correlation coefficient of the algorithm in this chapter and the comparative experimental results of different schemes are shown in Table 5. Compared with other algorithms, the cipher correlation coefficient of the algorithm proposed in this chapter is not inferior to other algorithms. Achieved better performance in image encryption effect. As can be seen from Table 4, the correlation coefficient of this algorithm is close to -1, which can reach the cipher standard. The correlation coefficient image of Pepper is shown in Figs 43–60.

**Table 4:** The plain correlation coefficient of the algorithm.

Image	Color	Plain			Cipher		
		horizontal	vertical	diagonal	horizontal	vertical	diagonal
**Pepper**	**Red**	0.9510	0.9458	0.9088	-0.0013	-0.0158	-0.0156
	**Green**	0.9633	0.9566	0.9336	-0.0032	0.0019	0.0211
	**Blue**	0.9459	0.9390	0.9047	0.0120	0.0147	-0.0025
	**Average**	0.9534	0.9471	0.9157	0.0025	0.0003	0.0010
**Plane**	**Red**	0.9713	0.9423	0.9117	0.0041	0.0162	-0.0182
	**Green**	0.9675	0.9411	0.9176	0.0117	-0.0045	-0.0123
	**Blue**	0.9437	0.9082	0.8728	0.0092	-0.0196	0.0187
	**Average**	0.9608	0.9305	0.9007	0.0083	-0.0026	-0.0039
**House**	**Red**	0.9341	0.9668	0.9179	0.0156	0.0056	0.0004
	**Green**	0.9506	0.9807	0.9351	0.0058	0.0104	-0.0054
	**Blue**	0.9749	0.9821	0.9607	0.0009	0.0058	0.0058
	**Average**	0.9532	0.9765	0.9379	0.0074	0.0073	0.0003
**Baboon**	**Red**	0.8678	0.9158	0.8553	0.0057	0.0003	0.0014
	**Green**	0.7571	0.7988	0.6999	-0.0077	-0.0219	-0.0217
	**Blue**	0.8718	0.8804	0.8039	0.0318	-0.0106	0.0072
	**Average**	0.8323	0.8650	0.7864	0.0099	0.0107	0.0044

**Table 5:** Correlation coefficient comparison.

Algorithm	Proposed	Ref. [13]	Ref. [14]	Ref. [15]	Ref. [16]	Ref. [17]
**horizontal**	0.0070	0.0039	0.0035	0.0021	0.0009	0.0025
**vertical**	0.0039	0.0059	-0.0025	0.0029	0.0071	-0.0012
**diagonal**	0.0005	-0.0050	-0.0002	0.0023	-0.0072	0.0001

As can be seen from Figs 43–60, there is a strong correlation between each pixel of the plain, while there is basically no correlation between each pixel of the cipher.

#### 4.2.4 Histogram

**Fig 61:**
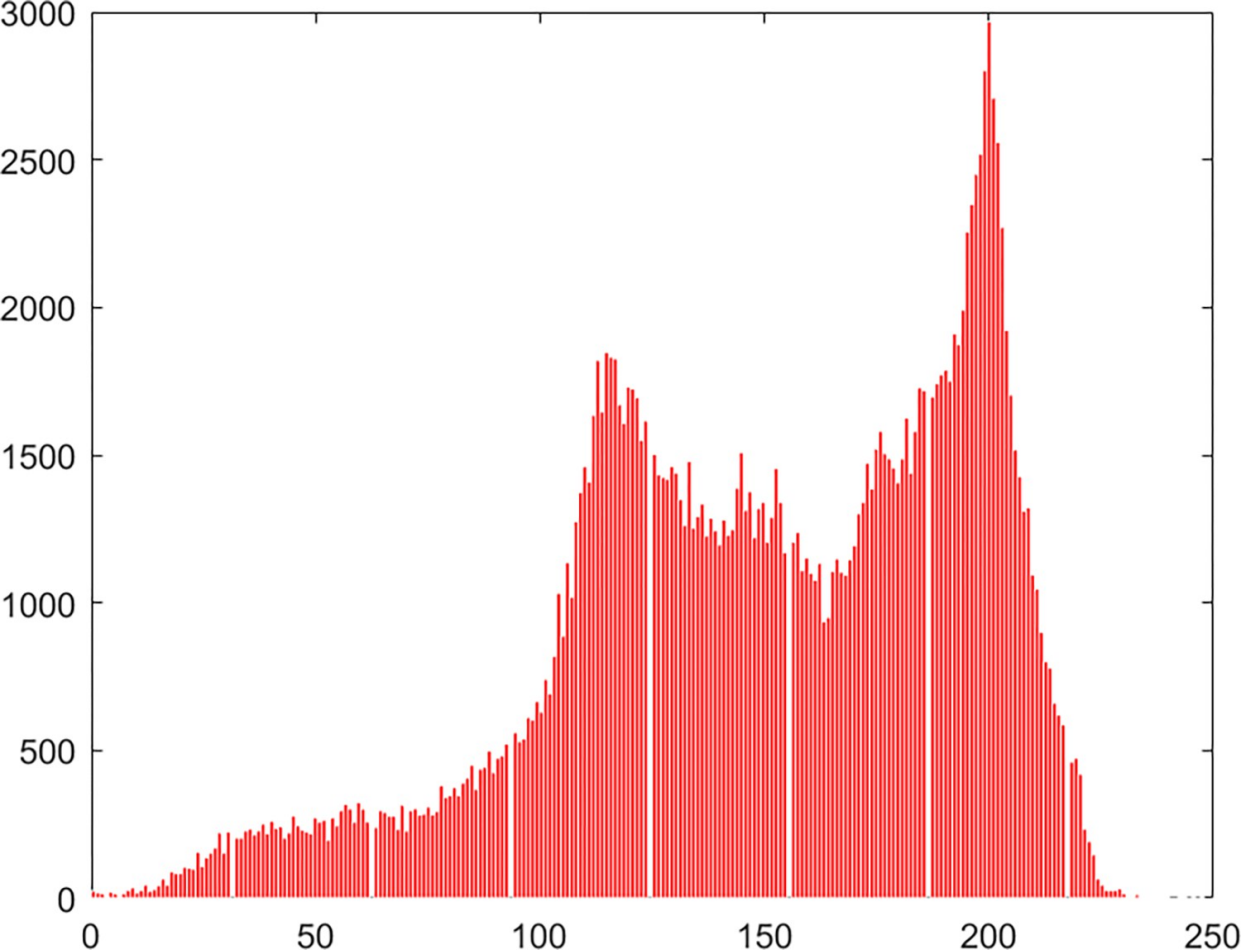
Red plain histogram.

**Fig 62:**
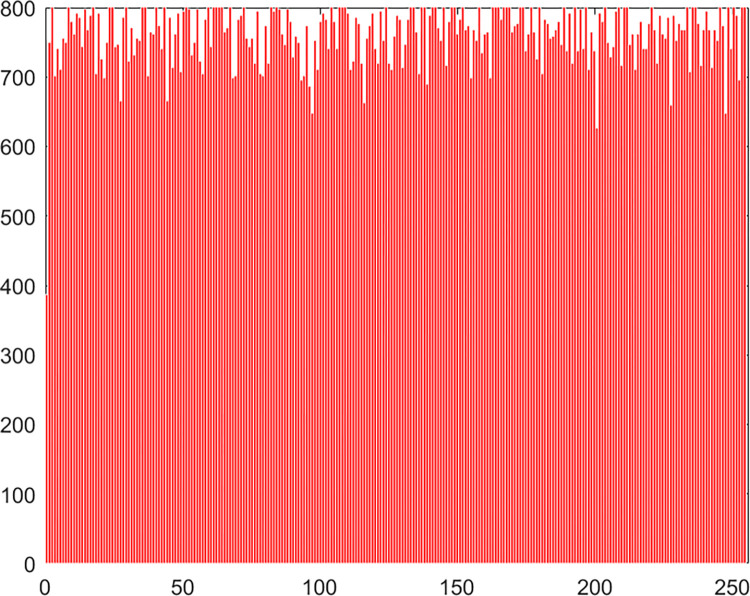
Red cipher histogram.

**Fig 63:**
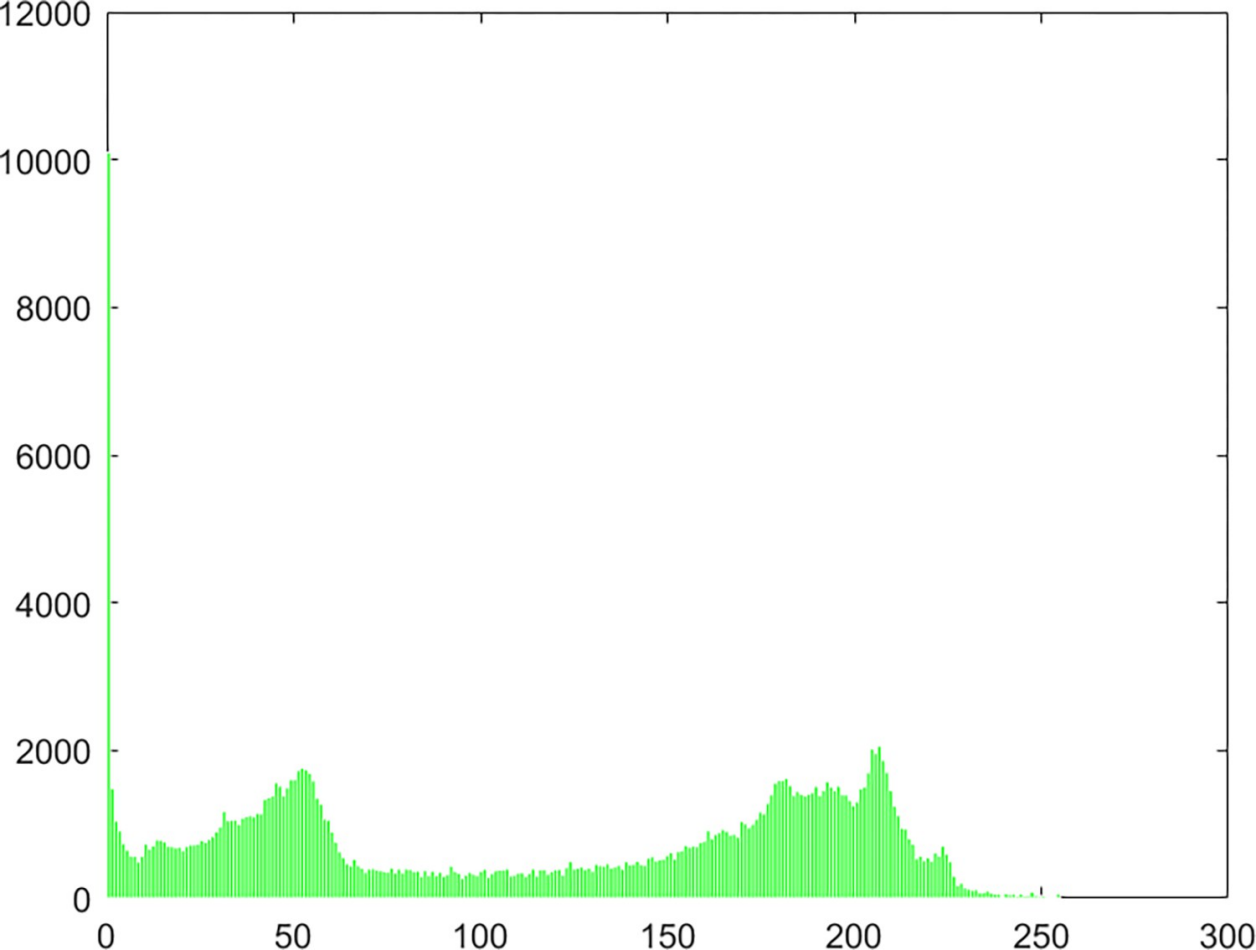
Green plain histogram.

**Fig 64:**
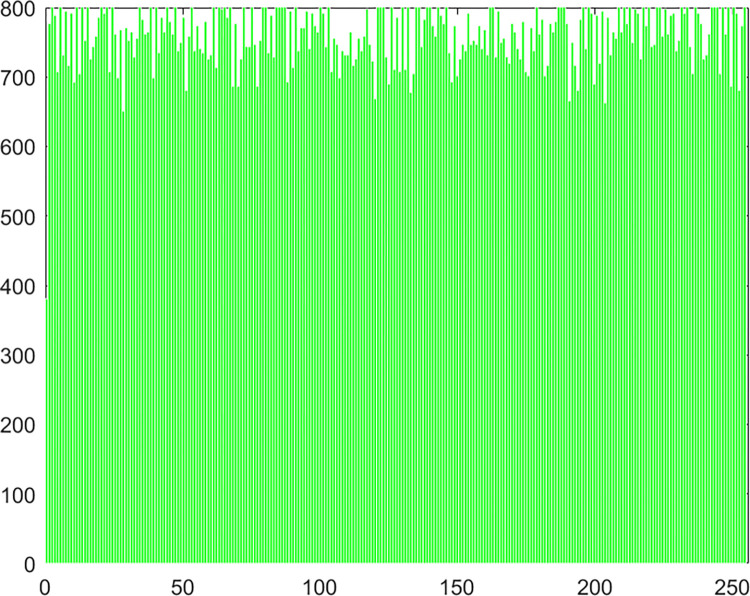
Green cipher histogram.

**Fig 65:**
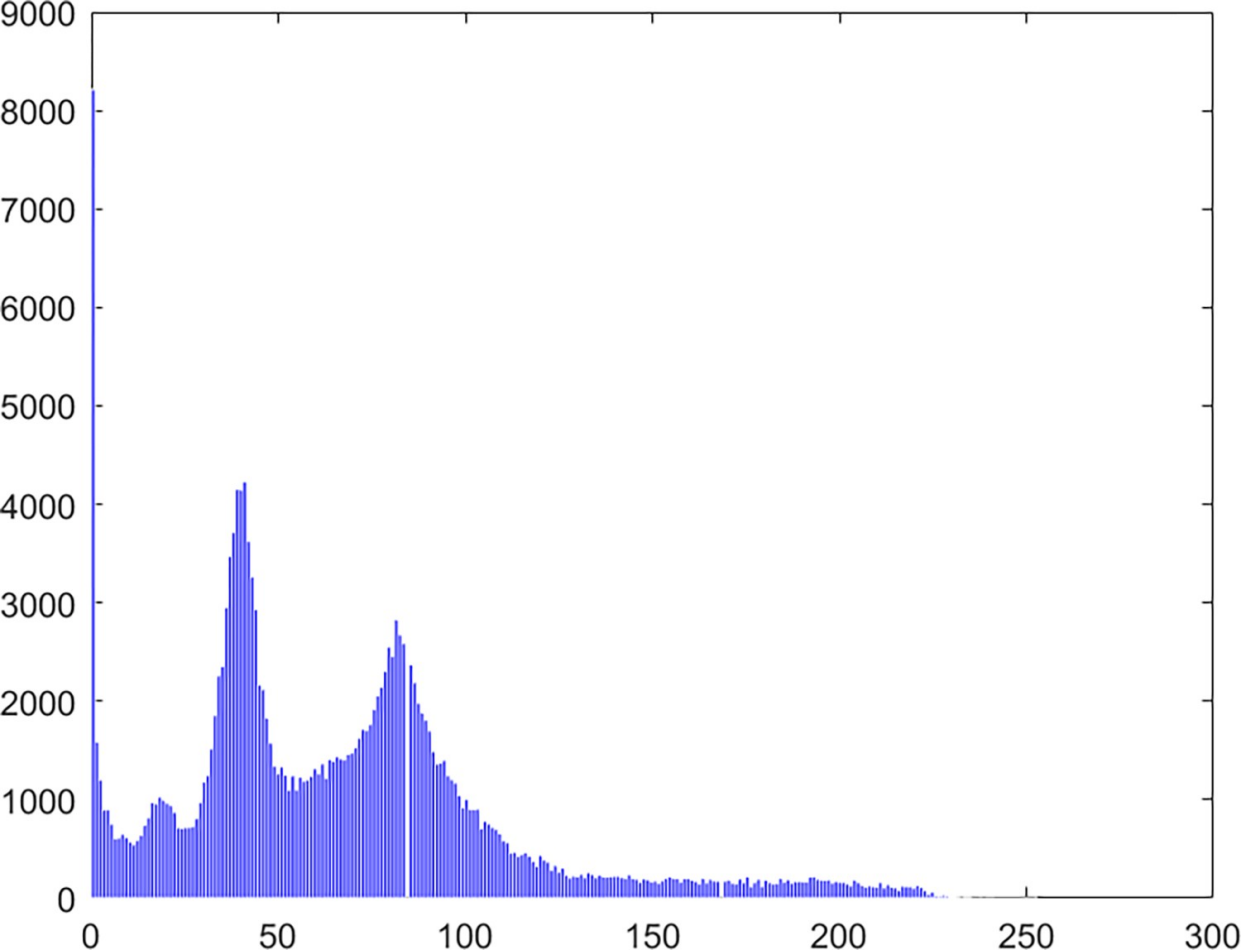
Blue plain histogram.

**Fig 66:**
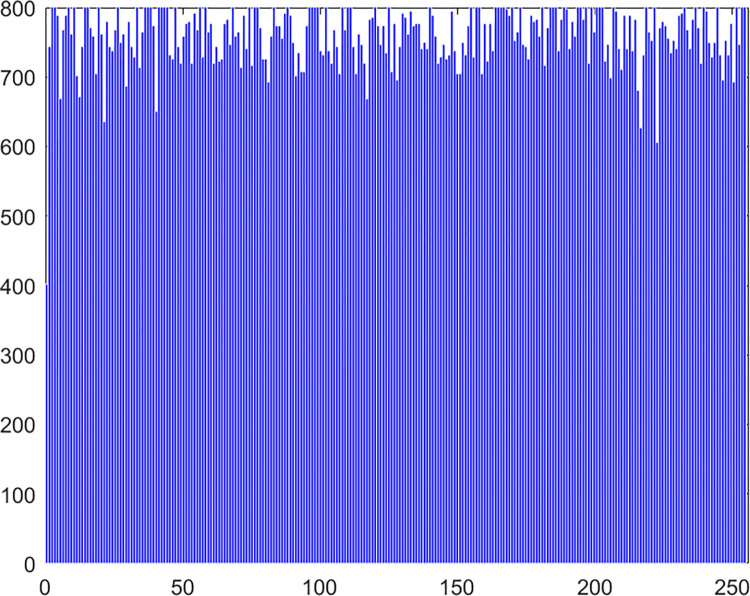
Blue cipher histogram.

Chaos Map Image Encryption Histogram is a tool used to visualize the distribution of pixels in an image. It shows the frequency distribution of pixel values for each color channel in the image. It helps in understanding the brightness and contrast distribution of an image. In chaos map image encryption, histograms can be used to analyze the quality of the encrypted and decrypted images and whether there are abnormal or unusual distributions. The histogram of the pepper image in this algorithm experiment is shown in Figs 61–66.

As can be seen from Figs 61–66, the value of each pixel in the cipher histogram encrypted by this algorithm is relatively balanced. This can meet encryption requirements.

#### 4.2.5 Information entropy

The information entropy of chaos map image encryption is an indicator used to measure the quality of image encryption. Information entropy is a concept that measures the uncertainty or confusion of information. In image encryption, information entropy can be used to evaluate the randomness and information protection capabilities of encrypted images. Theoretically, the closer to the theoretical value 8, the stronger the randomness. Information entropy is calculated through Eq (34).

$$H=-\sum_{i=0}^{L}p(i)\log_{2}p(i)$$ (34)

where *H* represents information entropy, *L* represents the number of grayscale levels of the image, and *p*(*i*) represents the probability of occurrence of grayscale value *i*.

Through the calculation of Equation (2.11), the information entropy of the plain and cipher images of the algorithm in this chapter is shown in Table 6. The information entropy value is accurate to four decimal places. The comparison between the average information entropy of this algorithm and other literature is shown in Table 7. It can be seen from the table that the information entropy of this algorithm is closer to the ideal value and can resist entropy attacks.

**Table 6:** Information entropy.

Image	Pepper	Plane	House	Baboon	Average
**Plain**	7.7139	7.7471	7.0686	7.7536	7.5721
**Cipher**	7.9995	7.9992	7.9993	7.9995	7.9994

**Table 7:** Information entropy comparison.

Algorithm	Proposed	Ref. [13]	Ref. [14]	Ref. [15]	Ref. [16]	Ref. [17]	Theoretical value
**Information entropy**	**7.9994**	7.9992	7.9992	7.9974	7.9937	7.9972	8

#### 4.2.6 Key sensitivity

The key sensitivity of chaos map image encryption refers to the sensitivity of the encryption process to small changes in the key. In a chaotic map, small changes in the key can lead to completely different encryption results. The greater the difference between the cipher, the better the performance of the encryption algorithm.

**Fig 67:**
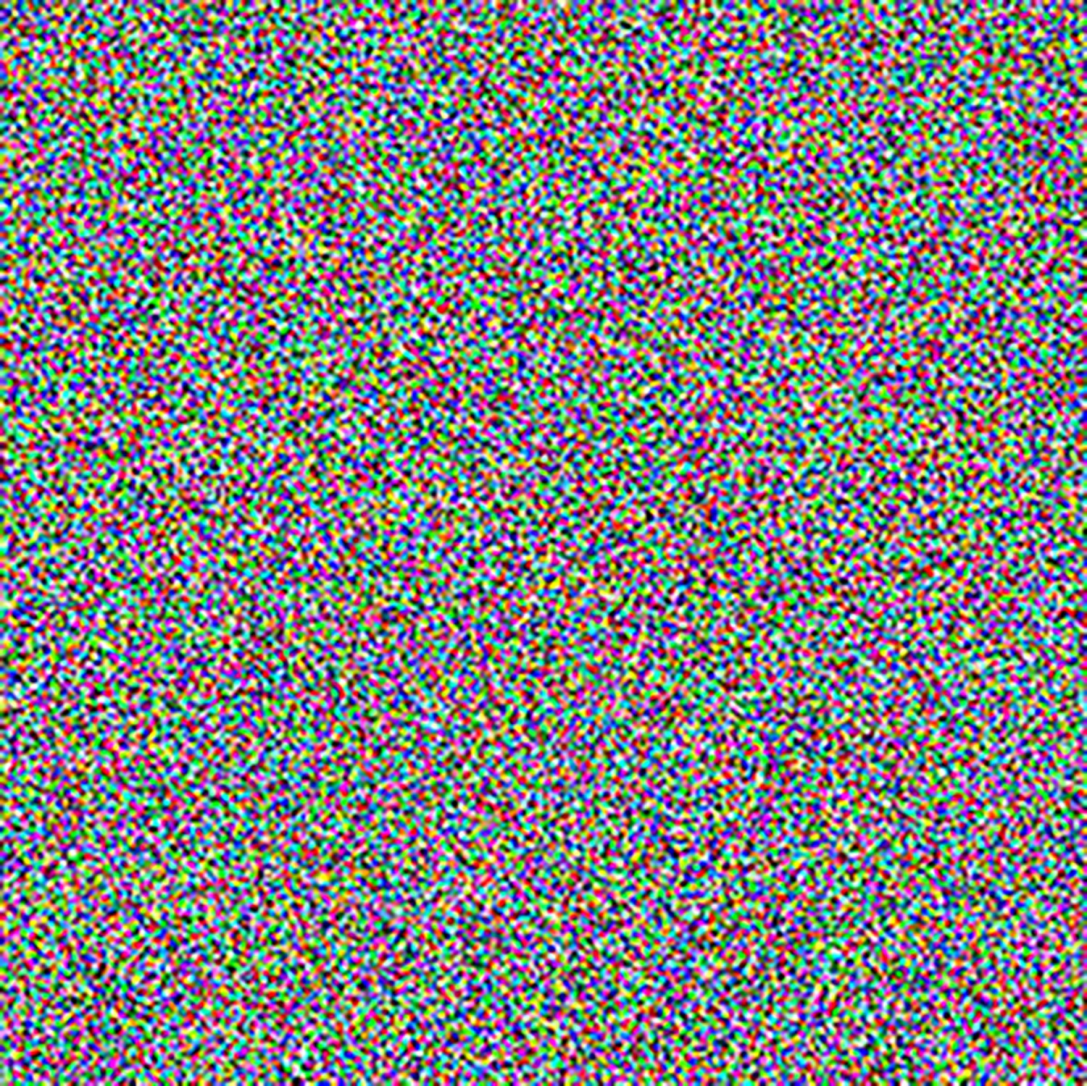
The first encrypted cipher.

**Fig 68:**
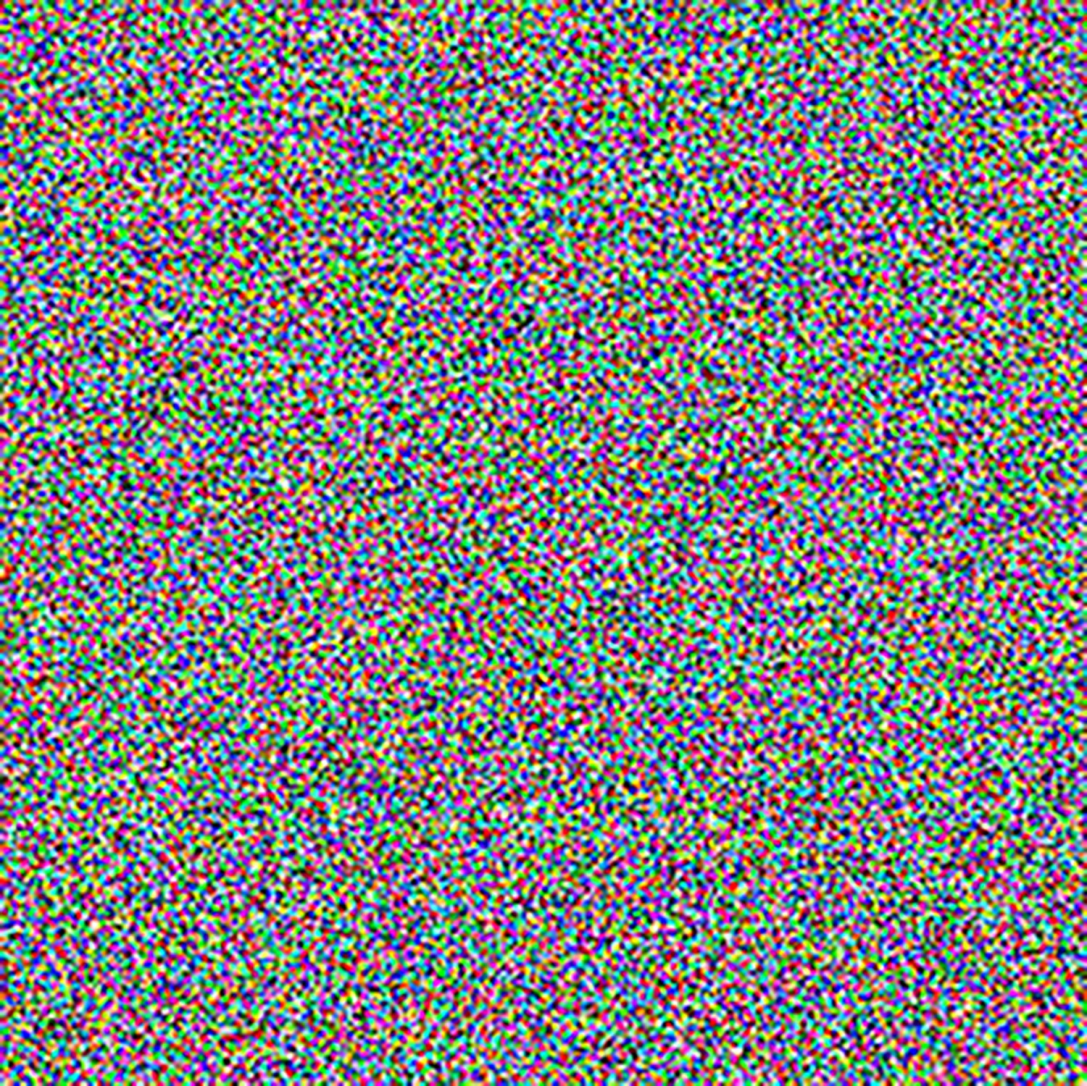
The cipher when the key changes by 10^−6^.

**Fig 69:**
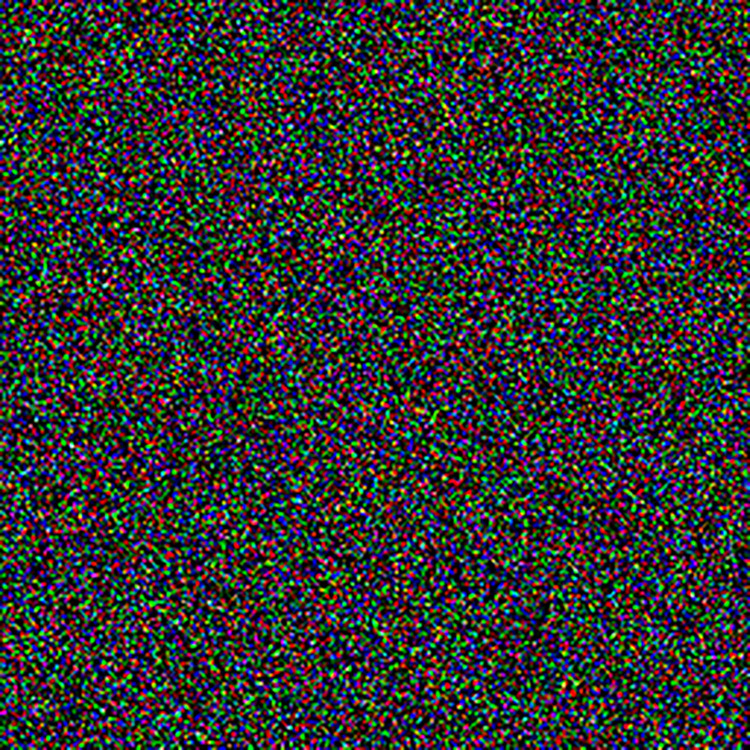
The difference between Fig 67 and Fig 68.

The detection of key sensitivity of this algorithm is shown in Figs 67–69. Fig 67 is the cipher encrypted for the first time. Fig 68 shows the cipher when the key changes by 10^−6^. Fig 69 shows the difference between the two ciphers. As you can see, the two pictures are quite different.

#### 4.2.7 Plain sensitivity

The plain sensitivity of chaotic map image encryption refers to the sensitivity to small changes in the plain image. During the encryption process, small changes in the plain image may lead to fundamental changes in the cipher image. The greater the difference between ciphers, the better the performance of the encryption algorithm, which can resist differential attacks or selected plain attacks. The plain sensitivity is calculated by Eqs (35)–(37).


$$NPCR(P_{1},P_{2})=\frac{1}{MN}\sum_{i=1}^{M}\sum_{j=1}^{N}\left|Sign(P_{1}(i,j)-P_{2}(i,j))\right|\times 100\%$$ (35)

$$Sign(a)=\{\begin{array}{ll} 1, & a>0\\ 0, & a=0\\ -1, & a<0 \end{array}$$ (36)

$$UACI(P_{1},P_{2})=\frac{1}{MN}\sum_{i=1}^{M}\sum_{j=1}^{N}\frac{\left|P_{1}(i,j)-P_{2}(i,j)\right|}{\sigma }\times 100\%$$ (37)

In Eq (35), *M* represents the length of the two images, and *N* represents the width of the two images. *NPCR* represents the ratio of the number of different pixels in two different images *P*_1_ and *P*_2_ to the number of all pixels. *Sign* () is a sign function, and its meaning is shown in Eq (36). The theoretical *NPCR* value of the two images is 99.6094%.

In Eq (37), *UACI* represents the average of the ratio of the difference to the maximum difference between the pixels at the corresponding positions of two different images *P*_1_ and *P*_2_. The theoretical value is 33.4635%. *σ* is the maximum difference between the two images.

The plain sensitivity experiment randomly changes the plain of each graph by 10^−8^ and iterates 100 times. Through the calculation of Eqs (35)–(37), the average experimental results are shown in Table 8. Accurate to four decimal places. The comparison of the average *NPCR* and *UACI* values of the algorithm experiments in this chapter with other literature is shown in Table 9. The algorithm in this chapter has achieved satisfactory performance, and *NPCR* and *UACI* are close to ideal values. Therefore, the encryption algorithm in this chapter is very sensitive to small changes in the plain image. It can effectively resist known plain attacks, chosen plain attacks and differential attacks.

**Table 8:** NPCR and UACI (%).

Algorithm	Pepper	Plane	House	Baboon	Average
***NPCR***	99.6174	99.6171	99.6184	99.6195	99.6172
***UACI***	33.4532	33.4652	33.4723	33.4681	33.4646

**Table 9:** NPCR and UACI comparison (%).

Algorithm	Proposed	Ref. [13]	Ref. [14]	Ref. [15]	Ref. [16]	Ref. [17]
**NPCR**	99.6172	99.6186	99.5693	**99.6142**	99.6299	99.6205
**UACI**	**33.4646**	33.4444	33.2981	33.4656	33.5027	33.5052

#### 4.2.8 Robustness

The robustness of chaos map image encryption refers to the ability of an encryption system to maintain its performance and protect image data in the face of various attacks and interferences. Robustness is one of the important indicators to measure the strength and reliability of encryption algorithms. This paper uses two methods, noise attack and loss of information, to test the performance of the image encryption algorithm.

**Fig 70:**
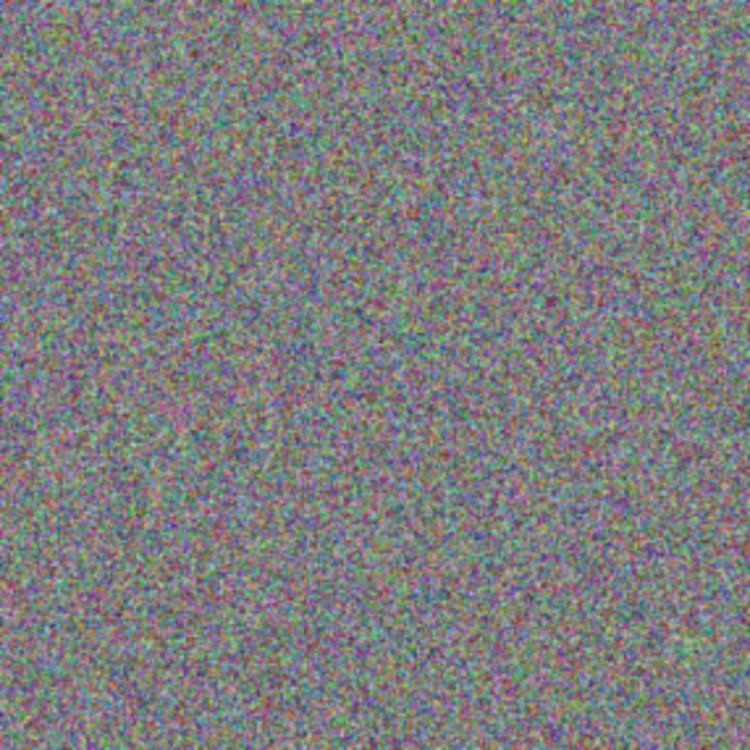
Add 1% noise cipher.

**Fig 71:**
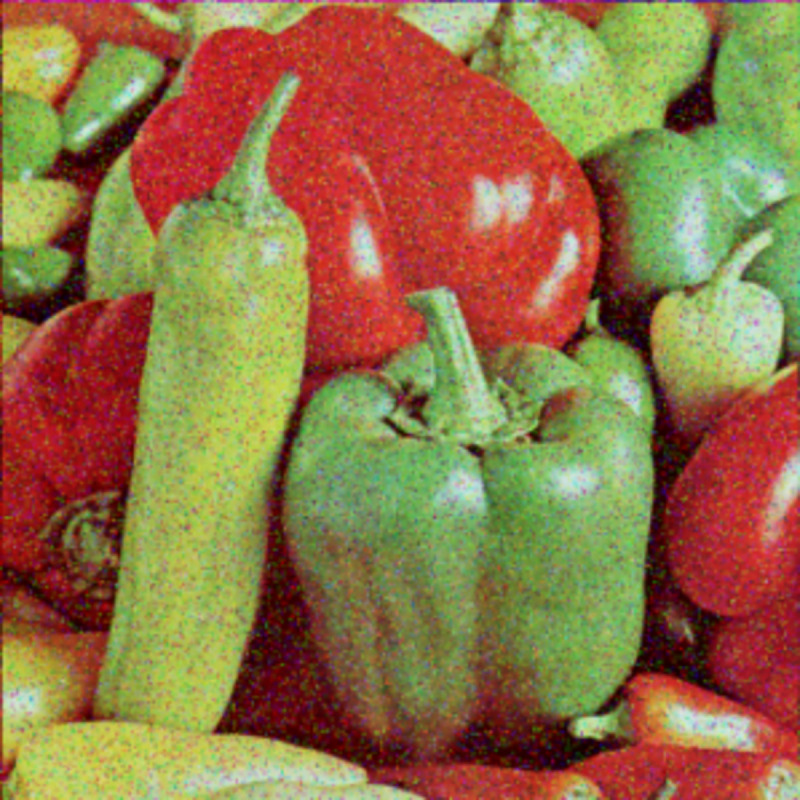
Fig 70 plain after decryption.

**Fig 72:**
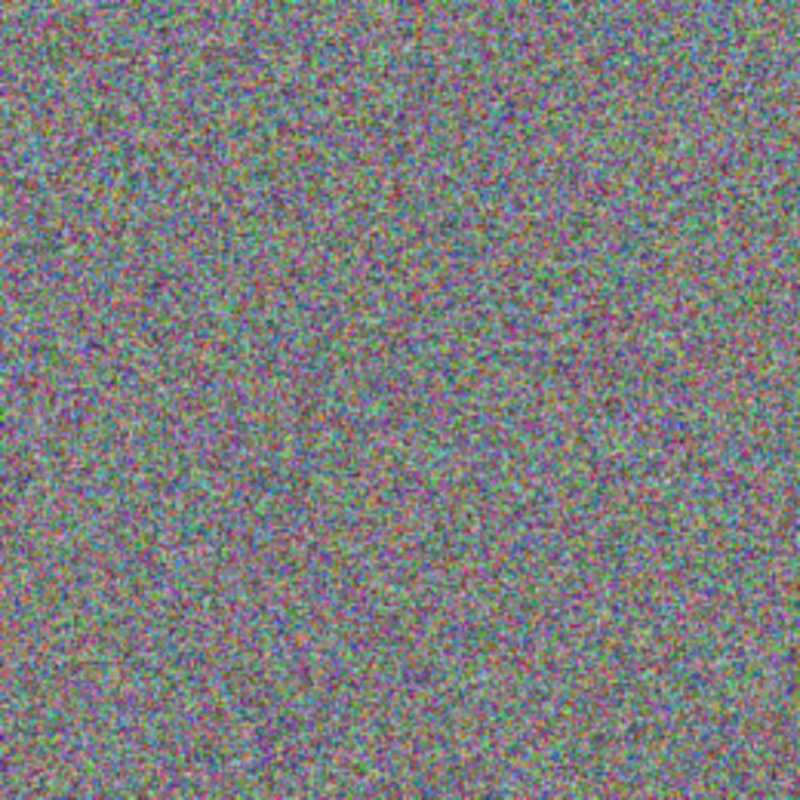
Add 3% noise cipher.

**Fig 73:**
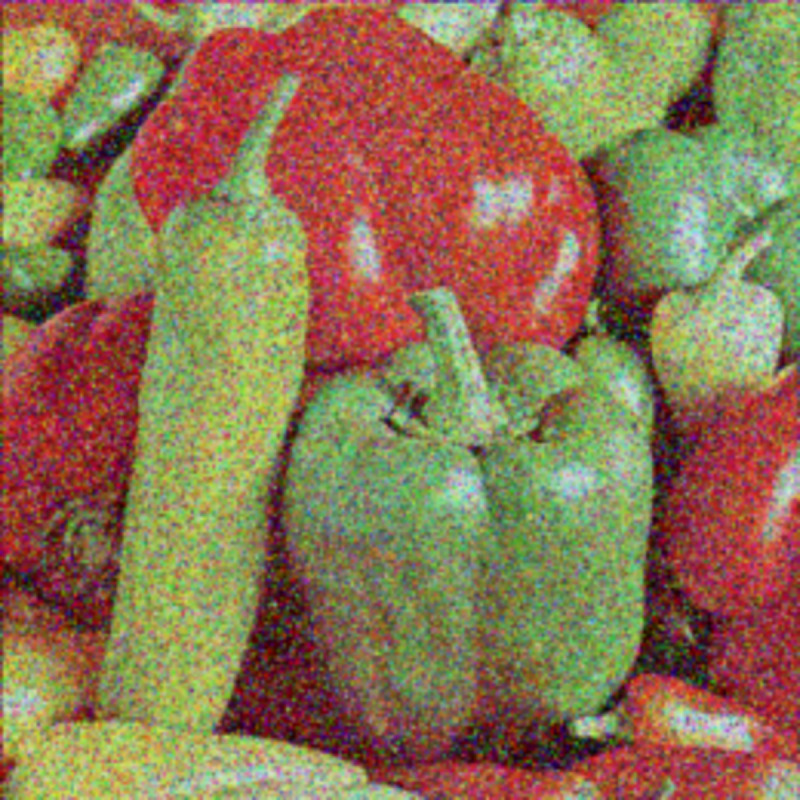
Fig 72 plain after decryption.

In order to evaluate the robustness of the algorithm in this chapter, 0.1% and 0.3% salt and pepper noise were added to the cipher image respectively. Then the noisy cipher image was decrypted. After decryption, the approximate original image information can still be restored. This shows that the algorithm is effective. Figs 70–73 show the result of adding noise to a cipher image and decrypted it.

**Fig 74:**
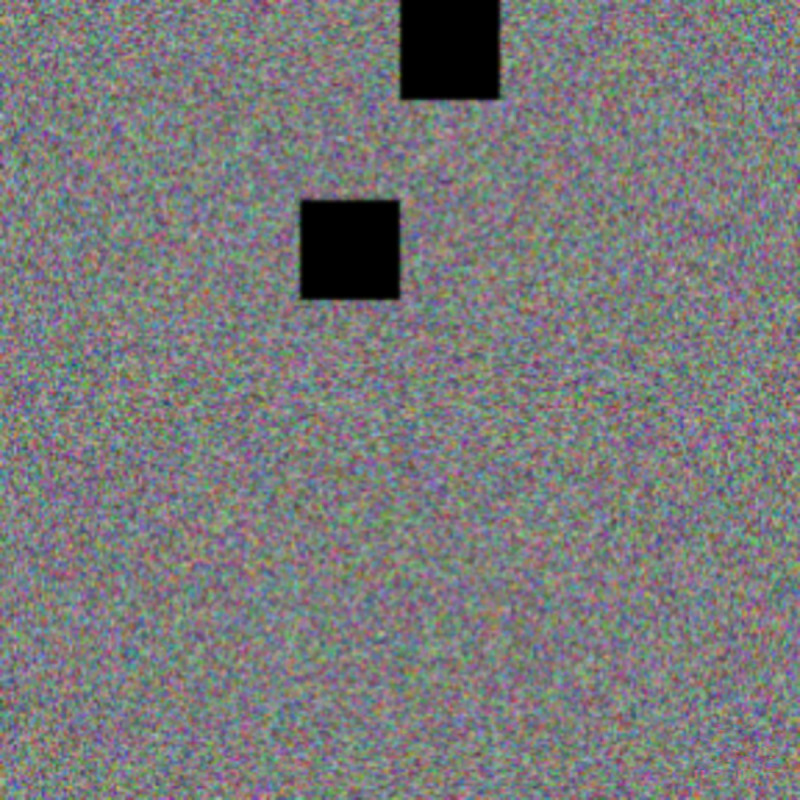
The cipher after some information is lost.

**Fig 75:**
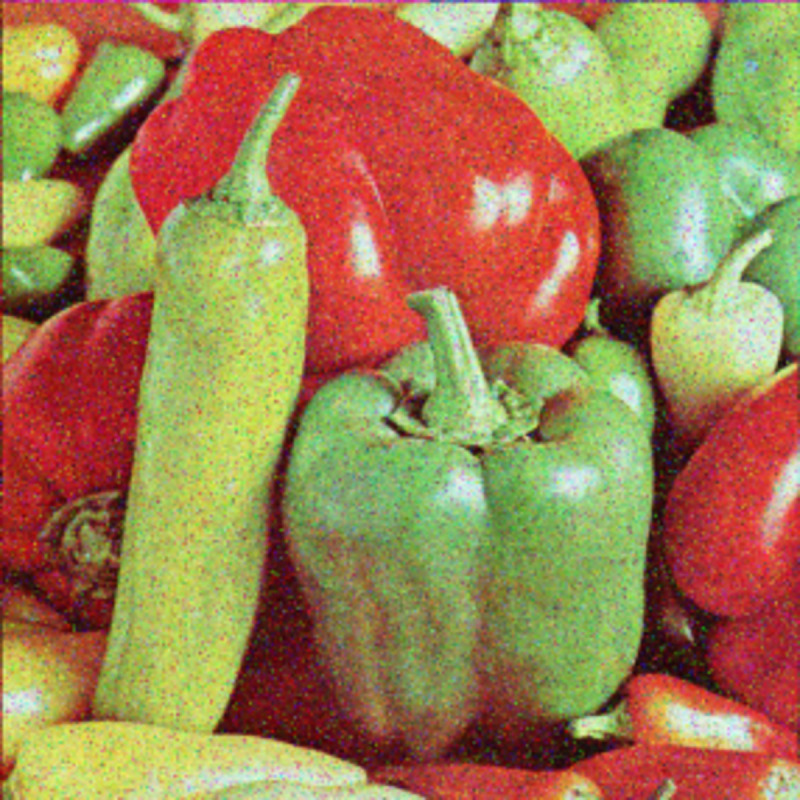
Fig 74 plain after decryption.

The loss of information experiment is shown in Figs 74, 75, taking the pepper image as an example. Fig 74 shows the cipher with part of the information lost. Fig 75 shows the decrypted plain of Fig 74. After losing some information, the basic information of the image can still be retained. Experiments show that the algorithm in this paper has good robustness and can resist certain noise attacks and information loss.

## 5 Conclusion

Image encryption algorithms based on chaos theory emerge in endlessly. Based on previous various chaotic image fast encryption algorithms, this paper proposes a color image sector fast encryption algorithm based on one-dimensional composite sinusoidal chaotic mapping. First, by combining four basic chaos maps in pairs and adding sine operations, six one-dimensional composite sinusoidal chaos maps (CSCM) are obtained. Second, select the two best chaotic mappings LCS and SCS, and verify the randomness of these two chaotic mappings through Lyapunov index and NIST SP 800–22 randomness tests. Third, the encryption process is unfolded according to the shape of a traditional Chinese fan, and the diffusion and scrambling of each pixel of the image are performed in parallel, greatly improving the encryption speed. When diffusing, changing the value of one pixel can affect the values of multiple subsequent pixels. When scrambling, each pixel changes position with the three pixels before it according to the chaotic sequence. Finally, multiple experiments have proven that this image encryption algorithm not only greatly improves the encryption and decryption speed, but also expands the key space, and can also meet the standards in terms of correlation coefficient, histogram, information entropy, key sensitivity, plain sensitivity, etc. The algorithm can also resist some common attacks and accidents, such as exhaustive attacks, differential attacks, entropy attack, noise attacks, information loss, diffusion or scrambling attack respectively, etc. The algorithm will continue to be optimized in the future. Strive to continue to improve the NPCR value and encryption speed of this algorithm.
